# Seasonal shifts and land-use impact: unveiling the gut microbiomes of bank voles (*Myodes glareolus*) and common voles (*Microtus arvalis*)

**DOI:** 10.1093/femsec/fiae159

**Published:** 2024-11-28

**Authors:** Lea Kauer, Christian Imholt, Jens Jacob, Christian Berens, Ralph Kühn

**Affiliations:** Molecular Zoology, Department of Zoology, TUM School of Life Sciences, Technical University of Munich, 85354 Freising, Germany; Julius Kühn-Institute, Federal Research Centre for Cultivated Plants, Institute for Epidemiology and Pathogen Diagnostics, Rodent Research, 48161 Münster, Germany; Julius Kühn-Institute, Federal Research Centre for Cultivated Plants, Institute for Epidemiology and Pathogen Diagnostics, Rodent Research, 48161 Münster, Germany; Friedrich-Loeffler-Institut, Institute of Molecular Pathogenesis, 07743 Jena, Germany; Molecular Zoology, Department of Zoology, TUM School of Life Sciences, Technical University of Munich, 85354 Freising, Germany; Department of Fish, Wildlife and Conservation Ecology, New Mexico State University, 8803 Las Cruces, NM, United States

**Keywords:** 16S, amplicon sequencing, bacterial microbiome, fungal microbiome, ITS, rodent microbiome

## Abstract

Gut microbial diversity influences the health and vitality of the host, yet it is itself affected by internal and external factors, including land-use. The impact of land-use practices on wild rodents’ gut microbiomes remains understudied, despite their abundance and potential as reservoirs for zoonotic pathogens. We examined the bacterial and fungal gut microbiomes of bank voles (*Myodes glareolus*) and common voles (*Microtus arvalis*) across grassland and forest habitats with varying land-use intensities and types. We collected rodents seasonally and used 16S rRNA and ITS amplicon sequencing for microbe identification. We found significant differences in alpha and beta diversities between the species, with *M. arvalis* exhibiting higher diversity. Seasonality emerged as a prominent factor influencing microbial diversity, with significant variations between sampling months. While land-use affects the gut microbiome, its impact is subordinate to seasonal variations. Differential abundance analysis underscores the dynamic nature of microbial composition, with seasonal changes playing a predominant role. Overall, our findings highlight the significant influence of seasonality on gut microbiome diversity and composition in wild rodents, reflecting dietary shifts associated with seasonal changes. Understanding the interplay between environmental factors and microbial communities in wild rodents enahnces our knowledge of ecosystem health and resilience, warranting further investigation.

## Teaser

While land-use has some effects on the compostion and diversity of the bacterial and fungal gut microbiomes of wild rodents, its impact is subordinate to the factor season.

## Introduction

The gut microbiome is increasingly being recognized for having a substantial impact on animal fitness, immune response, metabolism, and other physiological properties (Kohl and Dearing 2016, Rosshart et al. 2017, Suzuki 2017, Viney 2019). However, the microbiome itself is affected by environmental and host-specific factors (Benson et al. 2010). Previous research has demonstrated the impact of LUI and change on the microbiome across various habitats and their inhabitants within the agricultural landscape, including, but not limited to, soil (Idbella and Bonanomi 2023), birds (San Juan et al. 2020), amphibians (Barnes et al. 2021), insects (Nguyen and Rehan 2022), and mammals (Lavrinienko et al. 2018b). Rodents, the most abundant mammalian animal group, harbour a great variety of potentially pathogenic and zoonotic agents (Bordes et al. 2013, Han et al. 2015, Koskela et al. 2017), studies have predominantly focused on the influence of urbanization or environmental influences like radiation, metal pollution, or pathogens (Lavrinienko et al. 2018a, b, 2020, Brila 2024) on the microbiomes of selected rodent species, resulting in a knowledge gap in understanding the effects of agricultural and silvicultural land-use intensification (White and Razgour 2020).

Here, we investigated the bacterial and fungal gut microbial communities of two generalist wild rodent species, the bank vole (*Myodes glareolus*, Syn. *Clethrionomys glareolus*) and the common vole (*Microtus arvalis*), to discern differences in microbiome composition across varying land-use intensities within each species’ habitat. *M. glareolus* and *M. arvalis* belong to the most abundant rodent species in Europe (Mitchell-Jones et al. 1999, Wilson and Reeder 2005). Their population densities follow seasonal, annual, or multiannual cycles (Bujalska and Hansson 2000, Hansson et al. 2000, Jacob and Tkadlec 2010, Jacob et al. 2014). The two species differ in their diets and preferred habitats, with *M. glareolus* being omnivorous, consuming seeds, fruits, insects, invertebrates, fungi, and green vegetation (Gebczynska 1983, Hansson 1985b) in wooded and forested environments with dense understory cover (Mazurkiewicz 1994). Conversely, *M. arvalis* follow a herbivorous diet, predominantly feeding on grasses, seeds, and roots (Rinke 1990, Lüthi et al. 2010) in habitats characterized by undisturbed vegetation, such as meadows, grassland, set-aside land, and flower strips with cropped areas serving as secondary habitats (Jacob et al. 2014).

We used 16S rRNA gene and ITS amplicon sequencing to capture the bacterial and fungal gut microbiome of both bank voles and common voles caught on grassland and forest sites varying in land-use intensity (LUI).

## Materials and methods

### Sampling strategy

Rodents were caught on grassland and forest plots located in the Hainich-Dün region, comprising of the Hainich National Park and its surroundings in Thuringia, Germany. The plots are part of the long-term and large-scale DFG Biodiversity-Exploratories (DFG-BE) project (Fischer et al. 2010). To assess the LUI per plot, indices established for grassland (LUI) (23) and forest (silvicultural management index, SMI) (24) plots were calculated yearly. LUI in grasslands is quantified by mowing frequency, livestock grazing, and fertilization, while forestry intensity (SMI) is determined by tree species, stand age, and aboveground biomass (living and dead). Both indices range from 0 to 3.5, reflecting a gradient from low-intensity use (e.g. unfertilized sheep pastures or unmanaged forests) to high-intensity use (e.g. intensively fertilized cattle pastures or heavily managed conifer plantations). LUI and SMI are calculated for each plot based on questionnaires completed by land managers during interviews conducted by DFG-BE employees, with higher index values indicating greater LUI. Details on the calculation and the exploratories can be found in Fischer et al. (2010).

Rodents were caught three times in 2020 (June/July, September, and November) using metal snap traps. The different sampling months display different seasons of the year. Precipitation and average temperature differ greatly between the sampling months. In June, the average temperature is 16.2°C with an average precipitation of 66 mm; in September, the average temperature is 14.1°C with 57 mm precipitation, and in November, the average temperature is lowest with 4.9°C and 57 mm precipitation. We analysed bank voles caught on 15 forest plots and common voles caught on 15 grassland plots in 2020 (Imholt 2024).

The plots (Figure S1) were divided into three categories: low, medium, and high LUI, based on the continuous LUI and SMI values. Five plots from each intensity category were selected for both forest and grassland habitats, ensuring an even geographical distribution across the study area. The plots selected also represent different land-use types. In grassland, three types were analysed: pasture (only used as pasture, no mowing), meadow (not used as pasture, only mowed), and mowed pasture (plots used as pasture and mowed). Forest plots were categorized by the following four land-use types, according to the classification of Klarner et al. (2014): young-managed-beech, old-managed-beech, unmanaged-beech, and coniferous. Per plot, the microbiomes of approximately six animals were analysed (4–9 animals, mean 6.1 ± 0.8), yielding an even distribution of samples across sexes and sampling seasons. All procedures involving animal trapping were conducted according to relevant legislation and by permission of the Thuringian State Office of Consumer Protection (permit 22-2684-04-15-105/16). Details on the plots, their respective habitat, LUI and -type, and on the number and sex of the animals caught are provided in Table S1.

### DNA extraction and amplicon sequencing

DNA was extracted from feces derived from dissected guts using the Qiagen QIAamp DNA stool kit according to the IHMS protocol Q (Dore et al. 2020) with slight modifications (see Supplementary Information). We amplified the V4–5 region of the bacterial 16S rRNA gene and the ITS2 region of the fungal ITS gene. The V4–5 region was amplified using the primer pair 515fF-Y/926R (Parada et al. 2016) and the ITS2 region using the primer pair gITS7ngs (Tedersoo and Lindahl 2016) and ITS4ngs (White et al. 1990). Polymerase chain reaction (PCR) was performed using Q5^®^ High-Fidelity DNA Polymerase (New England Biolabs, Frankfurt/Main, Germany) and the following reaction conditions: 98°C for 30 s, followed by 30 cycles at 98°C for 10 s, at 56°C for 30 s, and at 72°C for 60 s, and then one cycle at 72°C for 2 min. A clean-up step was performed using the NucleoSpin^®^ Gel and PCR Clean-up kit (Macherey-Nagel, Düren, Germany). To be able to multiplex samples and distinguish multiple samples in one library, the samples were tagged with barcodes added to the 5′-end of each forward primer. We generated 16 robust barcodes using the R-package DNABarcodes version 1.32.0 (Buschmann and Bystrykh 2013) with the following parameters: barcode length = 9, dist = 5, metric = ‘seqlev’, and heuristic = ‘ashlock’.

Libraries were constructed according to the manufacturer’s instruction using the NEBNext Ultra II DNA Library Prep Kit for Illumina (New England Biolabs) and NEBNext Multiplex Oligos for Illumina 96 Unique Dual Index Primer Pairs Set1 (New England Biolabs). Paired-end (2 × 250 bp) sequencing was conducted by IMGM Laboratories (Martinsried, Germany) on a NovaSeq 6000 with the SP 500 v1.5 Kit (Illumina, Berlin, Germany) and the NovaSeq XP 2-Lane Kit v1.5 (Illumina). Raw sequence reads were submitted to the NCBI SRA database with the BioProject ID PRJNA1117218.

### Read data processing and statistical analysis

We used QIIME2, version 2023.2 (Bolyen et al. 2019), for the downstream analysis of the sequences generated. All analyses in R were conducted using version 4.3.2 (R Core Team 2022). The first steps, including demultiplexing, merging forward, and reverse paired-end reads were done identically for the 16S and ITS data in QIIME2.

For analysing the regions V4–5 of the bacterial 16S rRNA gene, we generated an amplicon sequence variants (ASV) table, using the standard DADA2 denoising pipeline (Callahan et al. 2016) including quality filtering and removal of chimeric sequences. For the analysis of the fungal *ITS2* region, we generated an operational taxonomic units (OTU) table (Nilsson et al. 2019), using open region clustering with a threshold of 97%. Taxonomic classification was conducted with self-trained taxonomic classifiers. We applied the feature-classifier fit-classifier-Naive-Bayes function (Pedregosa et al. 2011) in QIIME2 to build self-trained classifiers, trained on our region-specific primer sets. The classifiers were trained on preformated reference data (Abarenkov et al. 2022, Robeson et al. 2021) derived from the SILVA database (Quast et al. 2012) for 16S data and from the UNITE database (Abarenkov et al. 2023) for ITS data. ASVs identified as Archaea, Eukaryota, mitochondria, and Chloroplastida were removed from the 16S table as well as bacterial sequences not assigned to the domain level. Fungal sequences not assigned to the domain level were removed from the ITS OTU table. After taxonomic filtering, we applied prevalence filtering with a prevalence cutoff of 0.05 = 5% using the function phyloseq_filter_prevalence within R-package metagMisc version 0.5.0 (Mikryukov 2024). The Fast Tree method in QIIME2 was used to generate a rooted phylogenetic tree (Price et al. 2010). After quality control and rarefaction (V4–5: 15 000 reads and ITS2: 1565 reads per sample), the sample sets consisted of 146 samples for the V4–5 region and 133 samples for the *ITS2* region. In order to be able to distinguish between resident fungi, which are true inhabitants of the gut and other nonresident fungi, that only pass through the gut due to the host’s diet (Lavrinienko et al. 2021), we followed the filtering suggestions described in Watts et al. (2022): We used FUNGuild v.1.1 (Nguyen et al. 2016) to assign guilds or growth forms to the fungal OTUs. All OTUs, that were categorized as plant pathogens, epi- and endophytes, lichens, mycorrhizae, and wood saprophytes as well as OTUs with large fruiting bodies or growth forms were suspected to be nonresidential fungi and, therefore, excluded from further analysis. The remaining microfungi, yeasts, taxa associated with animals, and poorly known fungi were assumed to be resident fungi and therefore of interest for the analysis of the fungal microbiome.

We quantified microbial alpha and beta diversity for the bacterial and fungal microbiomes of bank voles and common voles. Alpha diversity was assessed in QIIME2 by four measurements [Pielou’s eveness, Faith’s phylogenetic diversity, number of features (ASVs or OTUs), and Shannon diversity] as was beta diversity (Bray–Curtis dissimilarity, Jaccard dissimilarity, and unweighted and weighted UniFrac).

The effect of host species on alpha and beta diversity was tested on the entire dataset. For further analyses, subset datasets based on habitat, respectively species, were used. This arises from the methodological fact that LUI quantification was calculated upon different variables within forest and grassland ecosystems (LUI on grassland and SMI on forest plots), thereby precluding direct comparison between LUI values of habitats.

Differences in alpha diversity among the two species were determined using a generalized linear mixed effects model with gamma distribution implemented in the R-package *lme4*, version 1.1.35.1 (Bates et al. 2014). For each model, the response variable was one of the four alpha diversity metrics, Plot was treated as random effect. The common vole alpha diversity was tested with season (June, September, and November), LUI and land-use type (pasture, meadow, and mowed pasture) as fixed variables. The bank vole alpha diversity was tested with season, LUI, and forest type based on the classification of Klarner et al. (2014) (young-managed-beech, old-managed-beech, unmanaged-beech, and coniferous) as fixed variables.

We analysed the effects of the same metadata variables on beta diversity by performing PERMANOVA using the function adonis in the R-package vegan version 2.6.4 (Dixon 2003) with 9999 permutations and pairwise PERMANOVA using the function pairwise.adonis2 in th R-package pairwiseAdonis, version 0.4 (Martinez Arbizu 2020). In order to control for the increased risk of type I errors associated with multiple testing, the Benjamini–Hochberg correction was applied to adjust *P*-values, ensuring that the false discovery rate was controlled at 5% (Benjamini and Hochberg 1995). Differential abundance analysis was conducted separately for the whole dataset (*M. arvalis* and *M. galreolus*) to distinguish bacterial and fungal families and gerena between species, as well as on the *M. arvalis* and *M. glareolus* dataset separately to asses the influence of season, LUI, and land-use type. Differential abundance analysis was performed at family level using the R-package ANCOM-BC version 2.4.0 (Lin and Peddada 2020) using a prevalence cutoff of 0.5.

## Results

A total of 14 329 085 paired-end reads were obtained after quality filtering and chimeric sequence removal of the bacterial dataset, with a median of 62 098 reads per sample. 2751 different bacterial taxa were detected. The fungal dataset contained 11 900 167 paired-end reads after quality filtering and chimera removal, with a median of 36 792 reads per sample. 1292 different fungal taxa were present. After classification with FUNGuild based on guild and growth morphology, 490 taxa were removed due to their likely status as nonresident taxa, leaving 802 taxa assumed to be resident fungal taxa. The following analysis of the fungal microbiome were conducted based on these 802 resident fungal taxa.

### Apha and beta diversity

#### Among Species

**Figure 1.**
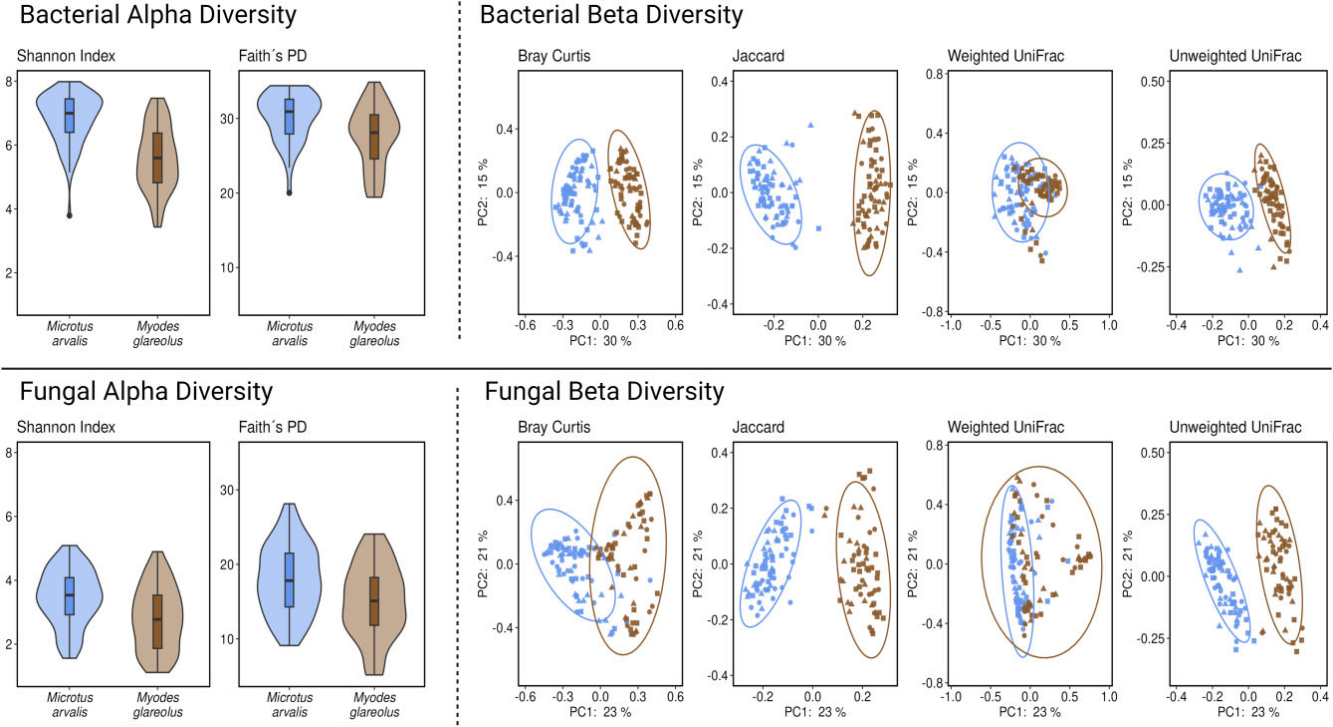
Bacterial and fungal alpha (Shannon Index and Faith’s PD) and beta diversities (Bray–Curtis, Jaccard, weighted UniFrac, and unweighted UniFrac) between the *M. arvalis* (left - blue) and *M. glareolus* (right - brown). Symbol shape in the beta diversity plots indicates LUI (triangle = high, square = medium, and circle = low).

Alpha and beta diversities of the bacterial and fungal gut microbiomes exhibited significantly lower diversity in *M. glareolus* than in *M. arvalis* based on all four alpha diversity measures (Shannon diversity, Pielou’s evenness, Faith’s PD, and number of features) and all four beta diversity measures (Bray–Curtis dissimilarity, Jaccard dissimilarity, unweighted, and weighted UniFrac distance) (Fig. 1, Tables S2 and S3). Overall, distinct alpha and beta diversity patterns were observed between the two species, with higher bacterial alpha diversity values determined for *M. arvalis*.

#### Within species

PERMANOVA analysis revealed that season (June, September, and November) exerted the highest influence on the bacterial and fungal microbiome compositions for both *M. glareolus* and *M. arvalis*, with highly significant *P*-values (< .001) and the highest *F*-values across all beta-diversity measures. LUI and land-use type (grassland: pasture, meadow, and mowed pasture; forest: young-managed-beech, old-managed-beech, unmanaged-beech, and coniferous) showed significant values only partially. However, their *F*-values were consistently lower compared to season (Table S4). Using the function betadisper, we tested if the dispersion is equivalent between groups. The results were inconsistent between the different groups as well as between the different beta diversity measures, indicating, that some of the observed differences in community composition in some of the beta diversity measures might be influenced by differences in variability within groups. Since our study design is balanced, differences in dispersion are less problematic but still need to be considered when interpreting the PERMANOVA results.

#### *Microtus arvalis*—grassland

For the common voles, we tested for differences in alpha and beta diversities based on the variables season, LUI, and land-use type. This revealed no significant differences in alpha diversity based on land-use type or intensity for both fungal and bacterial microbiomes. However, significant differences were observed in bacterial alpha diversity (Faith’s PD and number of features) between seasons, with the highest diversity observed in September and the lowest in June (Table S5).

Regarding beta diversity, significant differences were observed between seasons across all indices. While the beta diversity between high and medium LUI was not significant, it was significant between high and low, and medium and low intensities, except for weighted UniFrac. No significant differences were detected between mowed pastures and meadows in bacterial beta diversity, but significant differences were observed between mowed pasture and pasture, as well as between meadow and pasture, except for Bray–Curtis distance in the fungal microbiome (meadow–pasture) and for weighted UniFrac, in which no significant difference were detected for any comparison (Table S6).

#### *Myodes glareolus*—forest

For the bank vole dataset, we tested for differences in alpha and beta diversity values/data points based again on the variables season, LUI, and forest type. This revealed no significant influence of forest type on bacterial and fungal alpha diversities. However, significant differences were detected in fungal alpha diversity (Shannon diversity) between land-use intensities. Regarding seasonal variations, significant differences were observed between June and November in all alpha diversity parameters, except Pielou’s evenness. Bacterial alpha diversity was lowest in November and highest in September, while fungal alpha diversity was highest in November and lowest in June (Table S7).

Regarding beta diversity, significant differences were observed between seasons for all indices in both bacterial and fungal microbiomes. Additionally, LUI and forest type exerted significant influence within certain relationships on beta diversity, except for weighted UniFrac (see Table S8).

### Microbiome composition and differential abundance analysis

The bacterial gut microbiomes of bank voles and common voles were predominantly comprised by the three Phyla Bacillota 51.3% (*M. arvalis*: 47%, *M. glareolus*: 55.8%), Bacteroidota 19.2% (*M. arvalis*: 25.9%, *M. glareolus*: 11.3%), and Desulfobacterota 16.2% (*M. arvalis*: 11.2%, *M. glareolus*: 22.1%).

Similarly, the fungal gut microbiomes of common voles and bank voles were also comprised to about 99% by three Phyla; these were Ascomycota at 54% (*M. arvalis*: 59.6%, *M. glareolus*: 47.4%), Mucoromycota with 38.6% (*M. arvalis*: 34.8%, *M. glareolus*: 43.1%), and Basidiomycota to 7.3% (*M. arvalis*: 5.6%, *M. glareolus*: 9.4%).

**Figure 2.**
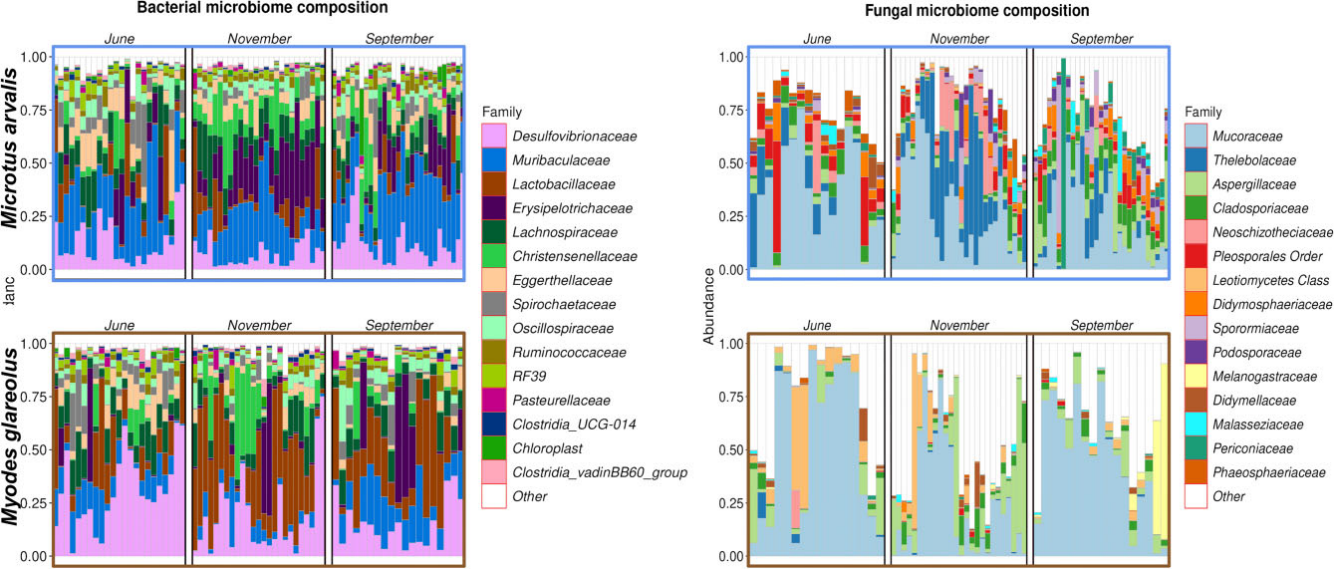
Taxonomic composition of the bacterial and fungal microbiomes of *M. arvalis* (top) and *M. glareolus* (bottom) on family level.

Within the gut microbiomes of common voles, the most abundant bacterial families were *Muribaculaceae* (23.8%), *Erysipelotrichaceae* (11.7%), *Desulfovibrionaceae* (11.2%), *Lachnospiraceae* (9.8%), and *Christensenellaceae* (7.9%). The most abundant fungal families were *Mucoraceae* (32.6%), *Thelebolaceae* (12.6%), *Cladosporiaceae* (7.3%), *Pleosporales Order* (6.9%), and *Aspergillaceae* (6.0%), see Fig. 2.

Within the gut microbiomes of bank voles, the most abundant bacterial families were *Desulfovibrionaceae* (22.1%), *Lactobacillaceae* (22.0%), *Muribaculaceae* (9.9%), *Lachnospiraceae* (9.5%), and *Erysipelotrichaceae* (6.4%). The most abundant fungal families were *Mucoraceae* (43.1%), *Aspergillaceae* (13.7%), *Leotiomycetes Class* (6.8%), *Cladosporiaceae* (4.6%), and *Saccharomycetales fam Incertae sedis* (3.1%), see Fig. 2.

The overall dataset comprised 33 bacterial and 43 fungal families identified in both species. Seventeen bacterial (55%) and 18 fungal families (42%) were found to be of significant differential abundance between *M. arvalis* and *M. glareolus* by ANCOM-BC analysis (Table S9 and Figure S2).

Further analysis within each host species showed 36 bacterial and 55 fungal families within the *M. arvalis* microbiomes (Table S10), and 32 bacterial and 46 fungal families within the *M. glareolus* microbiomes (Table S11). *Myodes glareolus* and *M. arvalis* share about 89% of the detected bacterial families and about 70% of the detected fungal families indicating a substantial overlap in bacterial and fungal families between the two species, albeit with differential abundance within each host species. Results of analysis at genus level are complementary and can be found in Tables S12–S14.

Both rodent species showed roughly the same percentage of bacterial and fungal families that were differentially abundant with respect to different land-use type, LUI, and season (bacterial families) (Table 1). In contrast, the percentages of differentially abundant fungal families differed considerably between the seasons in *M. glareolus* (15%) and *M. arvalis* (45%).

**Table 1.** Percentage of differentially abundant bacterial and fungal families between seasons, land-use intensities, and land-use types within *M. arvalis* and *M. glareolus*.

			Differentially abundant bacterial families *N* = 36			Differentially abundant fungal families *N* = 55		
			Within each pair of each category		Within each category	Within each pair of each category		Within each category
***M. arvalis***	June–November	Season	5	14%	25%	21	38%	45%
	June–September		4	11%		4	7%	
	High–low	LUI	1	3%	3%	4	7%	11%
	High–medium		0	0%		2	4%	
	MP—P	Land-use type	2	6%	6%	4	7%	13%
	MP—M		0	0%		3	5%	
			**Differentially abundant bacterial families *N* = 31**			**Differentially abundant fungal families *N* = 46**		
			**Within each pair of each category**		**Within each category**	**Within each pair of each category**		**Within each category**
***M. glareolus***	June–November	Season	4	13%	23%	1	2%	15%
	June–September		3	10%		6	13%	
	High–low	LUI	2	6%	10%	1	2%	7%
	High–medium		1	3%		2	4%	
	C—Old-MB	Land-use type	1	3%	3%	2	4%	15%
	C—Un-MB		0	0%		4	9%	
	C—Young-MB		0	0%		1	2%	

MP = mowed pasture, P = pasture, M = meadow, C = coniferous, Old-MB = old managed beech, Un-MB = unmanaged beech, and Young-MB = young managed beech.

Overall, ANCOM-BC analysis underlines the results of the diversity analysis, since we found more differentially abundant bacterial and fungal families between seasons than between land-use intensities and land-use types within both bank voles and common voles.

## Discussion

Detailed studies on how land-use, especially LUI, and season affect the microbiome, particularly the fungal microbiome of wild rodents, are scarce. In this study, we therefore examined the bacterial and fungal microbiomes of two rodent species, *M. arvalis* and *M. glareolus*, which are the dominant species in their respective grassland or forest habitats. We examined the differences in the bacterial and fungal microbiomes between these species and evaluated the impact of seasonality, LUI, and forest type on microbiome diversity and composition within each species.

### *Microtus arvalis* and *M. glareolus* have significantly different gut microbiomes

*Microtus arvalis* and *M. glareolus* exhibited significantly distinct gut microbiomes. Despite both belonging to the same subfamily (*Arvicoline*) of the *Cricetidae* family, their divergent dietary and habitat preferences likely account for the observed differences in microbiome compositions. *Microtus arvalis*, which mainly inhabit meadows and grassland, are folivorous and mostly feed on the aerial and underground vegetative part of plants (Butet and Delettre 2011). *Myodes glareolus* are omnivorous forest inhabitants and their dietary composition changes significantly between the seasons. Besides aerial and underground vegetative part of plants, they also substantially feed on invertebrates, seeds, fruits, and fungi (Butet and Delettre 2011).

High diversity of the microbiome of the herbivorous *M. arvalis* compared to the microbiome of the omnivorous *M. glareolus* is in line with the results of Ley et al. (2008) finding more diverse bacterial communities from carnivorous to omnivorous to herbivorous hosts. The same trend was found for fungal microbiomes, where in a study by Barelli et al. (2020), omnivorous primates showed a less diverse mycobiome than a leaf-eating specialist primate species.

Although our study focused on one species per habitat (*M. glareolus—*forest, *M. arvalis*—grassland), preventing definitive conclusions on species identity as the sole driver of microbiome differences, previous research suggests that species identity plays a significant role in microbiome composition. Even across different habitats, individuals from the same species tend to have more similar microbiota than individuals of different species from the same habitat (Knowles et al. 2019).

In our study, the bacterial microbiome was more diverse than the fungal microbiome in both *M. arvalis* and *M. glareolus* datasets, consistent with observations in other mammals (Parfrey et al. 2014, Nash et al. 2017). However, while bacterial and fungal diversities displayed similar significance patterns regarding the variables tested in the *M. arvalis* dataset, these patterns were not consistent across all measures in the *M. glareolus* dataset. This disparity may stem from fungi being a common part of the *M. glareolus* diet, but not in *M. arvalis*, potentially introducing noise into the analysis. Despite our efforts to filter out nonresident fungi, some may have remained, impacting the statistical significance of alpha and beta diversities measured in the *M. glareolus* dataset.

### Within *M. arvalis* and *M. glareolus*

The primary determinant of bacterial microbiome diversity and composition in both *M. glareolus* and *M. arvalis* was the sampling month (June/July, September, or November), representing seasonal variation. We observed significant differences in Faith’s PD index and all beta diversity parameters between seasons within the *M. arvalis* and *M. glareolus* datasets for both the bacterial and fungal microbiomes. These findings highlight that the substantial influence seasonal dietary shifts have on gut microbiomes (Hansson and Larsson 1978, Hoogenboom et al. 1984, Viro and Sulkava 1985, Hansson 1985a) is also observed for the bacterial and fungal gut microbiomes of *M. arvalis* and *M. glareolus*. Our findings align with previous studies demonstrating seasonal changes in rodent diets and their consequential effects on microbiome diversity and composition (Maurice et al. 2015, Marsh et al. 2022, Klure and Dearing 2023).

In contrast, the influence of LUI and type on alpha and beta diversity varied. Land-use type did not significantly affect alpha diversity in the *M. arvalis* or *M. glareolus* datasets for either bacteria or fungi. Only the Shannon index of the fungal dataset from *M. glareolus* showed a significant association with LUI.

Alpha diversity, while informative about species richness and evenness within a group, might not be sufficient to capture the complexity of ecological differences between groups. Beta diversity, on the other hand, provides insight into community composition, encompassing the identity and presence of taxa (Jaccard distance), their abundance (Bray–Curtis distance), and phylogenetic relationship (UniFrac distances), which makes beta diversity more sensitive to differences in species composition between groups. The different indices emphasize different features of compositional dataset. Jaccard and Unweighted UniFrac are based on presence or absence of taxa, whereas Bray–Curtis and weighted UniFrac incorporate abundance. UniFrac distances additionally consider the phylogenetic relationship between taxa. Within the *M. arvalis* and the *M. glareolus* datasets, the bacterial and fungal species composition within the tested variables (season, LUI, and land-use type) was relatively similar, suggesting that differences in abundance play a crucial role in distinguishing the microbioal communities. Consequently, Bray–Curtis and weighted UniFrac distances were more suitable to interpret our dataset compared to Jaccard and unweighted UniFrac distance. Weighted UniFrac adds more weight to the phylogenic relationships than to the abundances, it is consequently more unlikely to detect significant differences if the microbial community is dominated by a few highly abundant taxa that are closely related phylogenetically, or if the differences between communities are primarily driven by variations in the abundance of taxa. We observed significant differences in beta diversity between seasons across all indices within both the *M. arvalis* and *M. glareolus* dataset, while no clear trend was discernible for LUI or type. This lack of clear association may stem from the intricate interplay of factors contributing to ecosystem complexity within the study plots, potentially overshadowing the effects of land-use. Moreover, the robust influence of season, linked to dietary shifts, may mask the effects of other tested variables.

We found the most differentially abundant bacterial families between seasons followed by LUI and land-use type. For fungal families, the most differentially abundant families also occurred between seasons followed by land-use type and LUI. The amount of differentially abundant families was roughly the same between *M. arvalis* and *M. glareolus* except for the fungal composition between seasons, which changed from 45% in *M. arvalis* compared to 15% in *M. glareolus*. It is well-documented in the literature, that different bacterial and fungal taxa are linked to metabolic processes and the digestion of different dietary components (David et al. 2014, Precup and Vodnar 2019, Dahl et al. 2020, Mims et al. 2021, Tett et al. 2021, Banerjee et al. 2022). As a consequence, the substantial turnover in microbial composition throughout the year likely reflects the rodents’ seasonally varied diets. Nevertheless, the high proportion of differentially abundant fungal families within the *M. arvalis* dataset between the seasons is noteworthy and needs further investigation.

In summary, seasonal variation, primarily driven by dietary changes, exerted a significant influence on the diversity and composition of the bacterial and fungal microbiomes of *M. arvalis* and *M. glareolus*, while the impact of land-use type and intensity appeared subordinate in comparison.

## Supplementary Material

fiae159_Supplemental_File

## Data Availability

This work is partially based on data elaborated by the LaBiRo project of the Biodiversity Exploratories program (DFG Priority Program 1374). The datsets used have the IDs 31 360 and 31790. Raw sequence reads were submitted to the NCBI SRA database with the BioProject ID PRJNA1117218.
