# Supplementary material for: Seasonal shifts and land-use impact: unveiling the gut microbiomes of bank voles (Myodes glareolus) and common voles (Microtus arvalis)
Source: FEMS Microbiol Ecol. 2024 Nov 28;100(12):fiae159. doi: 10.1093/femsec/fiae159 (PMC11650868; doi:10.1093/femsec/fiae159)
Supplement: fiae159_Supplemental_File [file fiae159_supplemental_file.docx]

## Supplementary Information

**Bacterial and fungal DNA extraction from feces using the Qiagen QIAamp DNA stool kit**

according to: Dore (2020)

modified by: Lea Kauer

1. Homogenize 150 to 200 mg frozen feces with 1.0 mL ASL lysis buffer from the kit by vortexing for 2 min in a 2 mL tube containing 0.6 g of sterile zirconia beads, Ø 0.1mm [if the buffer shows a precipitate, heat to 70°C before use]

2. Incubate for 15 min at 95°C.

3. Cells are mechanically lysed by running the Bead Ruptor 12 for 6 min.

4. Samples are allowed to cool on ice for 2 min.

5. Samples are centrifuged at 16000 x g, 4°C, for 5 min.

6. Supernatant is transferred to a new 2 mL tube.

7. The pellet is mixed with 300 µL ASL lysis buffer from the kit, and steps 2-5 are repeated.

8. Supernatants are pooled in the new 2 mL tube.

9. Add 260 µl of 10 M ammonium acetate to each lysate tube, mix well, and incubate on ice for 5 min.

10. Centrifuge at 16000 x g, 4°C, for 10 min.

11. Transfer the supernatant to two 1.5 mL Eppendorf tubes, add one volume of isopropanol, mix well, and incubate on ice for 30 min.

12. Centrifuge at 16000 x g, 4°C, 15 min, remove the supernatant using aspiration, wash nucleic acid pellet with 70 % EtOH (0,5 mL) and dry the pellet under vacuum for 3 min.

13. Dissolve the nucleic acid pellet in 100 µL TE (Tris-EDTA) buffer overnight in a temperature-controlled shaker at 55°C and pool the two aliquots.

15. Add 15 µL proteinase K and 200 µL AL buffer to the supernatant, vortex for 15 s and incubate at 70°C for 10 min.

16. Add 200 µL of ethanol (96-100%) to the lysate and mix by vortexing.

17. Transfer to a QIAamp spin column and centrifuge at 16000 x g for 1min, at room temperature (RT).

18. Discard flow through, add 500 µL buffer AW1 (Qiagen) and centrifuge at 16000 x g for 1 min, at RT.

19. Discard flow through, add 500 µL buffer AW2 (Qiagen) and centrifuge at 16000 x g for 1 min, at RT.

20. Dry the column by centrifugation at RT for 1 min.

21. Add 200 µL Buffer AE (Qiagen), incubate for 1 min at RT.

22. Centrifuge for 1min at 16000 x g to elute DNA.

## Supplementary Tables

**Table S1:** Plot description and sample distribution per plot

| *Microtus arvalis* | | | | | | | | | |
| --- | --- | --- | --- | --- | --- | --- | --- | --- | --- |
| Plot ID | N June | N September | N November | N all | N Female | N Male | Land-use type | Land-use intensity numerical | Land-use intensity categorical |
| Heg01 | 2 | 2 | 2 | 6 | 4 | 2 | mowed pasture | 2,80 | high |
| Heg02 | 2 | 2 | 2 | 6 | 3 | 3 | mowed pasture | 2,61 | high |
| Heg03 | 2 | 2 | 2 | 6 | 4 | 2 | mowed pasture | 2,61 | high |
| Heg04 | 2 | 2 | 2 | 6 | 3 | 3 | meadow | 1,62 | medium |
| Heg05 | 2 | 2 | 2 | 6 | 3 | 3 | mowed pasture | 2,26 | medium |
| Heg07 | 2 | 2 | 2 | 6 | 3 | 3 | pasture | 3,47 | high |
| Heg09 | 2 | 2 | 2 | 6 | 3 | 3 | pasture | 0,83 | low |
| Heg11 | 2 | 2 | 2 | 6 | 3 | 3 | mowed pasture | 1,83 | medium |
| Heg20 | 2 | 2 | 2 | 6 | 3 | 3 | pasture | 0,83 | low |
| Heg26 | 2 | 2 | 2 | 6 | 2 | 4 | meadow | 2,17 | medium |
| Heg35 | 2 | 2 | 2 | 6 | 3 | 3 | mowed pasture | 2,64 | high |
| Heg37 | 2 | 2 | 2 | 6 | 2 | 4 | meadow | 1,21 | medium |
| Heg42 | 2 | 2 | 2 | 6 | 2 | 4 | pasture | 0,19 | low |
| Heg43 | 2 | 5 | 2 | 8 | 5 | 3 | pasture | 0,36 | low |
| Heg44 | 2 | 2 | 2 | 6 | 3 | 3 | pasture | 0,92 | low |
|  |  |  |  |  |  |  |  |  |  |
| *Myodes glareolus* | | | | | | | | | |
| Plot ID | N June | N September | N November | N all | N Female | N Male | Land-use type | Land-use intensity numerical | Land-use intensity categorical |
| Hew1 | 2 | 2 | 2 | 6 | 3 | 3 | conferious | 1,87 | high |
| Hew3 | 2 | 2 | 2 | 6 | 4 | 2 | conferious | 2,20 | high |
| Hew4 | 2 | 2 | 2 | 6 | 3 | 3 | young-managed-beech | 1,89 | high |
| Hew5 | 2 | 2 | 3 | 7 | 4 | 3 | young-managed-beech | 0,96 | medium |
| Hew6 | 2 | 2 | 2 | 6 | 3 | 3 | old-managed-beech | 0,75 | low |
| Hew7 | 2 | 2 | 2 | 6 | 3 | 3 | old-managed-beech | 0,69 | low |
| Hew11 | 1 | 1 | 2 | 4 | 3 | 3 | unmanaged-beech | 0,52 | low |
| Hew13 | 2 | 2 | 2 | 6 | 4 | 2 | conferious | 2,25 | high |
| Hew17 | 2 | 2 | 2 | 6 | 3 | 3 | young-managed-beech | 1,11 | medium |
| Hew20 | 2 | 2 | 2 | 6 | 3 | 3 | young-managed-beech | 0,99 | medium |
| Hew21 | 5 | 2 | 2 | 9 | 3 | 6 | old-managed-beech | 0,81 | medium |
| Hew22 | 2 | 2 | 2 | 6 | 3 | 3 | old-managed-beech | 0,64 | low |
| Hew35 | 3 | 0 | 3 | 6 | 3 | 3 | unmanaged-beech | 0,86 | medium |
| Hew40 | 2 | 2 | 2 | 6 | 4 | 2 | unmanaged-beech | 0,02 | low |
| Hew51 | 2 | 2 | 2 | 6 | 3 | 3 | conferious | 2,26 | high |

**Table S2:** Generalized linear mixed effects model results bacterial (V4-5) and fungal (ITS2) gut microbiota alpha-diversity within each species. Bold text indicates statistical significance.

| Bacterial Alpha Diversity | | | | |
| --- | --- | --- | --- | --- |
| **Diversity Index** | **Variable** | **Estimate** | **Std. Error** | ***p*** |
| Shannon | Intercept | 1.892 | 0.025 | **<0.001** |
|  | *Myodes glareolus* | -0.202 | 0.036 | **<0.001** |
| Pielou´s Evenness | Intercept | -0.235 | 0.021 | **<0.001** |
|  | *Myodes glareolus* | -0.132 | 0.031 | **<0.001** |
| Faith´s PD | Intercept | 3.262 | 0.020 | **<0.001** |
|  | *Myodes glareolus* | -0.162 | 0.028 | **<0.001** |
| Number of features | Intercept | 5.843 | 0.037 | **<0.001** |
|  | *Myodes glareolus* | -0.391 | 0.053 | **<0.001** |
|  |  |  |  |  |
| Fungal Alpha Diversity | | | | |
| **Diversity Index** | **Variable** | **Estimate** | **Std. Error** | ***p*** |
| Shannon | Intercept | 1.227 | 0.056 | **<0.001** |
|  | *Myodes glareolus* | -0.205 | 0.081 | **<0.001** |
| Pielou´s Evenness | Intercept | -0.586 | 0.044 | **<0.001** |
|  | *Myodes glareolus* | -0.163 | 0.064 | **0.022** |
| Faith´s PD | Intercept | 2.879 | 0.045 | **<0.001** |
|  | *Myodes glareolus* | -0.166 | 0.066 | **0.014** |
| Number of features | Intercept | 4.275 | 0.063 | **<0.001** |
|  | *Myodes glareolus* | -0.183 | 0.091 | **0.019** |

**Table S3:** PERMANOVA results for bacterial (V4-5) and fungal (ITS2) gut microbiota beta-diversity within each species. Bold text indicates statistical significance.

| Bacterial Beta Diversity | | | | | |
| --- | --- | --- | --- | --- | --- |
| **Dissimilarity Index** | **Variable** | **Sum of squares** | **R2** | ***F*** | ***p*** |
| Bray-Curtis | Species | 6.286 | 0.111 | 18.678 | **<0.001** |
| Jaccard |  | 6.640 | 0.117 | 19.272 | **<0.001** |
| Unweighted UniFrac |  | 2.332 | 0.124 | 21.164 | **<0.001** |
| Weighted UniFrac |  | 2.766 | 0.151 | 26.495 | **<0.001** |
|  |  |  |  |  |  |
| Fungal Beta Diversity | | | | | |
| **Dissimilarity Index** | **Variable** | **Sum of squares** | **R2** | ***F*** | **p** |
| Bray-Curtis | Species | 6.105 | 0.127 | 19.073 | **<0.001** |
| Jaccard |  | 4.272 | 0.091 | 13.098 | **<0.001** |
| Unweighted UniFrac |  | 3.297 | 0.099 | 14.442 | **<0.001** |
| Weighted UniFrac |  | 4.398 | 0.111 | 16.414 | **<0.001** |

**Table S4:** PERMANOVA results for bacterial (V4-5) and fungal (ITS2) gut microbiota beta-diversity in (A) *Microtus arvalis* and (B) *Myodes glareolus*; p-values corrected by Benjamin-Hochberg correction. Bold text indicates statistical significance.

| **A** | Bacterial Beta Diversity | |  |  |  |  |  | | | |  | **B** | Bacterial Beta Diversity | | |  | | |  |  |  | |
| --- | --- | --- | --- | --- | --- | --- | --- | --- | --- | --- | --- | --- | --- | --- | --- | --- | --- | --- | --- | --- | --- | --- |
|  | **Dissimilarity Index** | **Variable** | **Sum of squares** | **R2** | **F** | ***Corrected p*** | |  |  | **Dissimilarity Index** | | | | **Variable** | **Sum of squares** | | **R2** | **F** | | ***Corrected p*** | |  |
|  | Bray-Curtis | Season | 1.846 | 0.078 | 3.286 | **<0.001** | |  |  | Bray-Curtis | | | | Season | 1.5643 | | 0.068 | 2.4108 | | **<0.001** | |  |
|  |  | Land-use intensity | 0.374 | 0.016 | 1.330 | 0.083 | |  |  |  |  |  |  | Land-use intensity | 0.4412 | | 0.019 | 1.359 | | 0.069 | |  |
|  |  | Land-use type | 0.751 | 0.032 | 1.336 | 0.106 | |  |  |  |  |  |  | Forest type | 1.120 | | 0.049 | 1.151 | | **0.038** | |  |
|  | Jaccard | Season | 1.524 | 0.068 | 2.803 | **<0.001** | |  |  | Jaccard | | | | Season | 1.362 | | 0.068 | 2.372 | | **<0.001** | |  |
|  |  | Land-use intensity | 0.389 | 0.017 | 1.434 | 0.234 | |  |  |  |  |  |  | Land-use intensity | 0.307 | | 0.154 | 1.068 | | **0.006** | |  |
|  |  | Land-use type | 0.742 | 0.033 | 1.365 | **0.019** | |  |  |  |  |  |  | Forest type | 1.025 | | 0.051 | 1.191 | | **0.001** | |  |
|  | Unweighted UniFrac | Season | 0.613 | 0.081 | 3.414 | **<0.001** | |  |  | Unweighted UniFrac | | | | Season | 0.502 | | 0.077 | 2.714 | | **<0.001** | |  |
|  |  | Land-use intensity | 0.142 | 0.018 | 1.58 | 0.239 | |  |  |  |  |  |  | Land-use intensity | 0.104 | | 0.016 | 1.125 | | **0.019** | |  |
|  |  | Land-use type | 0.262 | 0.034 | 1.457 | 0.055 | |  |  |  |  |  |  | Forest type | 0.351 | | 0.054 | 1.263 | | **0.012** | |  |
|  | Weighted UniFrac | Season | 0.792 | 0.106 | 4.560 | **<0.001** | |  |  | Weighted UniFrac | | | | Season | 0.766 | | 0.125 | 4.599 | | **<0.001** | |  |
|  |  | Land-use intensity | 0.066 | 0.009 | 0.764 | 0.829 | |  |  |  |  |  |  | Land-use intensity | 0.053 | | 0.008 | 0.640 | | 0.585 | |  |
|  |  | Land-use type | 0.209 | 0.028 | 1.207 | 0.829 | |  |  |  |  |  |  | Forest type | 0.178 | | 0.029 | 0.713 | | 0.402 | |  |
|  |  |  |  |  |  |  | |  |  |  | | | |  |  | |  |  | |  | |  |
|  | Fungal Beta Diversity | |  |  |  |  | |  |  | Fungal Beta Diversity | | | | |  | |  |  | |  | |  |
|  | **Dissimilarity Index** | **Variable** | **Sum of squares** | **R2** | **F** | ***Corrected p*** | |  |  | **Dissimilarity Index** | | | | **Variable** | **Sum of squares** | | **R2** | **F** | | ***Corrected p*** | |  |
|  | Bray-Curtis | Season | 3.102 | 0.146 | 6.164 | **<0.001** | |  |  | Bray-Curtis | | | | Season | 2.624 | | 0.127 | 4.248 | | **<0.001** | |  |
|  |  | Land-use intensity | 0.368 | 0.017 | 1.464 | 0.125 | |  |  |  |  |  |  | Land-use intensity | 0.492 | | 0.024 | 1.593 | | 0.078 | |  |
|  |  | Land-use type | 0.823 | 0.039 | 1.635 | **0.045** | |  |  |  |  |  |  | Forest type | 1.468 | | 0.071 | 1.584 | | **0.032** | |  |
|  | Jaccard | Season | 1.673 | 0.073 | 2.893 | **<0.001** | |  |  | Jaccard | | | | Season | 1.614 | | 0.081 | 2.597 | | **<0.001** | |  |
|  |  | Land-use intensity | 0.642 | 0.023 | 2.221 | **<0.001** | |  |  |  |  |  |  | Land-use intensity | 0.395 | | 0.019 | 1.273 | | **0.047** | |  |
|  |  | Land-use type | 1.053 | 0.046 | 1.821 | **<0.001** | |  |  |  |  |  |  | Forest type | 1.327 | | 0.067 | 1.423 | | **<0.001** | |  |
|  | Unweighted UniFrac | Season | 1.261 | 0.078 | 3.129 | **<0.001** | |  |  | Unweighted UniFrac | | | | Season | 1.345 | | 0.095 | 3.088 | | **<0.001** | |  |
|  |  | Land-use intensity | 0.420 | 0.026 | 2.085 | **<0.001** | |  |  |  |  |  |  | Land-use intensity | 0.323 | | 0.023 | 1.482 | | **0.025** | |  |
|  |  | Land-use type | 0.813 | 0.05 | 2.018 | **<0.001** | |  |  |  |  |  |  | Forest type | 0.923 | | 0.065 | 1.413 | | **0.007** | |  |
|  | Weighted UniFrac | Season | 1.905 | 0.117 | 4.719 | **<0.001** | |  |  | Weighted UniFrac | | | | Season | 2.814 | | 0.148 | 5.022 | | **<0.001** | |  |
|  |  | Land-use intensity | 0.217 | 0.013 | 1.075 | 0.350 | |  |  |  |  |  |  | Land-use intensity | 0.557 | | 0.029 | 1.989 | | 0.070 | |  |
|  |  | Land-use type | 0.513 | 0.032 | 1.271 | 0.298 | |  |  |  |  |  |  | Forest type | 1.381 | | 0.072 | 1.644 | | 0.070 | |  |

**Table S5:** Generalized linear mixed effects model results for (A) bacterial (V4-5) and (B) fungal (ITS2) gut microbiota alpha-diversity in *Microtus arvalis*; p-values corrected by Benjamin-Hochberg correction. Bold text indicates statistical significance.

| **A** | Season | |  |  |  |  | | | |  | **B** | Season | | |  | |  | |  | |  |
| --- | --- | --- | --- | --- | --- | --- | --- | --- | --- | --- | --- | --- | --- | --- | --- | --- | --- | --- | --- | --- | --- |
|  | **Diversity Index** | **Variable** | **Estimate** | **Std. Error** | ***Corrected p*** | |  |  | **Diversity Index** | | | | **Variable** | **Estimate** | | **Std. Error** | | ***Corrected p*** | |  |  |
|  | Shannon | Intercept | 1.899 | 0.025 | **<0.001** | |  |  | Shannon | | | | Intercept | 1.216 | | 0.066 | | **<0.001** | |  |  |
|  |  | November | 0.007 | 0.032 | 0.819 | |  |  |  |  |  |  | November | -0.059 | | 0.081 | | 0.463 | |  |  |
|  |  | September | 0.058 | 0.031 | 0.106 | |  |  |  |  |  |  | September | 0.102 | | 0.081 | | 0.309 | |  |  |
|  | Pielou´s Evenness | Intercept | -0.237 | 0.023 | **<0.001** | |  |  | Pielou´s Evenness | | | | Intercept | -0.586 | | 0.055 | | **<0.001** | |  |  |
|  |  | November | -0.025 | 0.026 | 0.346 | |  |  |  |  |  |  | November | -0.058 | | 0.068 | | 0.391 | |  |  |
|  |  | September | 0.031 | 0.026 | 0.336 | |  |  |  |  |  |  | September | 0.072 | | 0.067 | | 0.391 | |  |  |
|  | Faith´s PD | Intercept | 3.416 | 0.028 | **<0.001** | |  |  | Faith´s PD | | | | Intercept | 2.837 | | 0.069 | | **<0.001** | |  |  |
|  |  | November | 0.131 | 0.036 | **<0.001** | |  |  |  |  |  |  | November | -0.044 | | 0.079 | | 0.958 | |  |  |
|  |  | September | 0.119 | 0.035 | **0.001** | |  |  |  |  |  |  | September | 0.103 | | 0.079 | | 0.288 | |  |  |
|  | Number of features | Intercept | 6.146 | 0.058 | **<0.001** | |  |  | Number of features | | | | Intercept | 4.218 | | 0.091 | | **<0.001** | |  |  |
|  |  | November | 0.193 | 0.075 | **0.016** | |  |  |  |  |  |  | November | -0.018 | | 0.101 | | 0.853 | |  |  |
|  |  | September | 0.166 | 0.074 | **0.024** | |  |  |  |  |  |  | September | 0.128 | | 0.100 | | 0.299 | |  |  |
|  |  |  |  |  |  | |  |  |  | | | |  |  | |  | |  | |  |  |
|  | Land-use intensity | |  |  |  | |  |  | Land-use intensity | | | | |  | |  | |  | |  |  |
|  | **Diversity Index** | **Variable** | **Estimate** | **Std. Error** | ***Corrected p*** | |  |  | **Diversity Index** | | | | **Variable** | **Estimate** | | **Std. Error** | | ***Corrected p*** | |  |  |
|  | Shannon | Intercept | 1.957 | 0.028 | **<0.001** | |  |  | Shannon | | | | Intercept | 1.249 | | 0.068 | | **<0.001** | |  |  |
|  |  | Land-use intensity | -0.019 | 0.014 | 0.164 | |  |  |  |  |  |  | Land-use intensity | -0.009 | | 0.033 | | 0.780 | |  |  |
|  | Pielou´s Evenness | Intercept | -0.248 | 0.031 | **<0.001** | |  |  | Pielou´s Evenness | | | | Intercept | -0.560 | | 0.058 | | **<0.001** | |  |  |
|  |  | Land-use intensity | -0.015 | 0.015 | 0.316 | |  |  |  |  |  |  | Land-use intensity | -0.012 | | 0.029 | | 0.676 | |  |  |
|  | Faith´s PD | Intercept | 3.537 | 0.065 | **<0.001** | |  |  | Faith´s PD | | | | Intercept | 2.879 | | 0.080 | | **<0.001** | |  |  |
|  |  | Land-use intensity | -0.018 | 0.017 | 0.297 | |  |  |  |  |  |  | Land-use intensity | -0.001 | | 0.040 | | 0.971 | |  |  |
|  | Number of features | Intercept | 6.316 | 0.072 | **<0.001** | |  |  | Number of features | | | | Intercept | 4.279 | | 0.108 | | **<0.001** | |  |  |
|  |  | Land-use intensity | -0.023 | 0.036 | 0.516 | |  |  |  |  |  |  | Land-use intensity | -0.009 | | 0.054 | | 0.860 | |  |  |
|  |  |  |  |  |  | |  |  |  | | | |  |  | |  | |  | |  |  |
|  | Land-use type | |  |  |  | |  |  | Land-use type | | | | |  | |  | |  | |  |  |
|  | **Diversity Index** | **Variable** | **Estimate** | **Std. Error** | ***Corrected p*** | |  |  | **Diversity Index** | | | | **Variable** | **Estimate** | | **Std. Error** | | ***Corrected p*** | |  |  |
|  | Shannon | Intercept | 1.915 | 0.025 | **<0.001** | |  |  | Shannon | | | | Intercept | 1.212 | | 0.053 | | **<0.001** | |  |  |
|  |  | Pasture | 0.014 | 0.034 | 0.764 | |  |  |  |  |  |  | Pasture | 0.081 | | 0.069 | | 0.372 | |  |  |
|  |  | Meadow | 0.013 | 0.044 | 0.764 | |  |  |  |  |  |  | Meadow | -0.077 | | 0.088 | | 0.385 | |  |  |
|  | Pielou´s Evenness | Intercept | -0.286 | 0.025 | **<0.001** | |  |  | Pielou´s Evenness | | | | Intercept | -0.596 | | 0.044 | | **<0.001** | |  |  |
|  |  | Pasture | 0.019 | 0.035 | 0.597 | |  |  |  |  |  |  | Pasture | 0.057 | | 0.059 | | 0.495 | |  |  |
|  |  | Meadow | 0.015 | 0.044 | 0.728 | |  |  |  |  |  |  | Meadow | -0.049 | | 0.074 | | 0.503 | |  |  |
|  | Faith´s PD | Intercept | 3.495 | 0.030 | **<0.001** | |  |  | Faith´s PD | | | | Intercept | 2.850 | | 0.057 | | **<0.001** | |  |  |
|  |  | Pasture | 0.018 | 0.042 | 0.665 | |  |  |  |  |  |  | Pasture | 0.089 | | 0.076 | | 0.356 | |  |  |
|  |  | Meadow | 0.016 | 0.053 | 0.766 | |  |  |  |  |  |  | Meadow | -0.057 | | 0.095 | | 0.551 | |  |  |
|  | Number of features | Intercept | 6.295 | 0.059 | **<0.001** | |  |  | Number of features | | | | Intercept | 4.238 | | 0.068 | | **<0.001** | |  |  |
|  |  | Pasture | -0.029 | 0.081 | 0.714 | |  |  |  |  |  |  | Pasture | 0.129 | | 0.088 | | 0.215 | |  |  |
|  |  | Meadow | -0.039 | 0.104 | 0.715 | |  |  |  |  |  |  | Meadow | -0.125 | | 0.109 | | 0.256 | |  |  |

**Table S6:** Pairwise PERMANOVA results for (A) bacterial (V4-5) and (B) fungal (ITS2) gut microbiota beta-diversity in *Microtus arvalis*; p-values corrected by Benjamin-Hochberg correction. Bold text indicates statistical significance.

| **A** | Season | |  |  |  |  |  | | | |  | **B** | Season | | |  | | |  | |  |  | |  |
| --- | --- | --- | --- | --- | --- | --- | --- | --- | --- | --- | --- | --- | --- | --- | --- | --- | --- | --- | --- | --- | --- | --- | --- | --- |
|  | **Dissimilarity Index** | **Variable** | **Sum of squares** | **R2** | ***F*** | ***Corrected p*** | |  |  | **Dissimilarity Index** | | | | **Variable** | **Sum of squares** | | **R2** | ***F*** | | ***Corrected p*** | | |  |  |
|  | Bray-Curtis | June - September | 0.629 | 0.038 | 2.048 | **0.002** | |  |  | Bray-Curtis | | | | June - September | 0.808 | | 0.067 | 3.114 | | **0.001** | | |  |  |
|  |  | June - November | 1.350 | 0.091 | 4.813 | **0.001** | |  |  |  |  |  |  | June - November | 1.175 | | 0.095 | 4.565 | | **0.001** | | |  |  |
|  |  | September - November | 0.780 | 0.052 | 2.877 | **0.001** | |  |  |  |  |  |  | September - November | 2.436 | | 0.148 | 9.396 | | **0.001** | | |  |  |
|  | Jaccard | June - September | 0.637 | 0.042 | 2.229 | **0.001** | |  |  | Jaccard | | | | June - September | 0.583 | | 0.432 | 1.942 | | **0.001** | | |  |  |
|  |  | June - November | 1.039 | 0.072 | 3.731 | **0.001** | |  |  |  |  |  |  | June - November | 0.977 | | 0.069 | 3.230 | | **0.001** | | |  |  |
|  |  | September - November | 0.674 | 0.045 | 2.517 | **0.001** | |  |  |  |  |  |  | September - November | 0.948 | | 0.054 | 3.105 | | **0.001** | | |  |  |
|  | Unweighted UniFrac | June - September | 0.320 | 0.062 | 3.401 | **0.001** | |  |  | Unweighted UniFrac | | | | June - September | 0.444 | | 0.047 | 2.133 | | **0.002** | | |  |  |
|  |  | June - November | 0.425 | 0.086 | 4.505 | **0.001** | |  |  |  |  |  |  | June - November | 0.813 | | 0.084 | 3.920 | | **0.002** | | |  |  |
|  |  | September - November | 0.216 | 0.045 | 2.485 | **0.001** | |  |  |  |  |  |  | September - November | 0.650 | | 0.052 | 2.976 | | **0.002** | | |  |  |
|  | Weighted UniFrac | June - September | 0.201 | 0..39 | 2.091 | 0.058 | |  |  | Weighted UniFrac | | | | June - September | 0.781 | | 0.084 | 3.928 | | **0.003** | | |  |  |
|  |  | June - November | 0.451 | 0.096 | 5.118 | **0.003** | |  |  |  |  |  |  | June - November | 0.087 | | 0.083 | 3.887 | | **0.006** | | |  |  |
|  |  | September - November | 0.497 | 0.106 | 6.285 | **0.003** | |  |  |  |  |  |  | September - November | 1.177 | | 0.096 | 5.715 | | **0.006** | | |  |  |
|  |  |  |  |  |  |  | |  |  |  | | | |  |  | |  |  | |  | | |  |  |
|  | Land-use intensity | |  |  |  |  | |  |  | Land-use intensity | | | | |  | |  |  | |  | | |  |  |
|  | **Dissimilarity Index** | **Variable** | **Sum of squares** | **R2** | ***F*** | ***Corrected p*** | |  |  | **Dissimilarity Index** | | | | **Variable** | **Sum of squares** | | **R2** | ***F*** | | ***Corrected p*** | | |  |  |
|  | Bray-Curtis | high - medium | 0.325 | 0.022 | 1.097 | 0.245 | |  |  | Bray-Curtis | | | | high - medium | 0.326 | | 0.025 | 1.105 | | 0.338 | | |  |  |
|  |  | high - low | 0.536 | 0.031 | 1.784 | **0.018** | |  |  |  |  |  |  | high - low | 0.395 | | 0.029 | 1.431 | | 0.219 | | |  |  |
|  |  | medium - low | 0.466 | 0.029 | 1.492 | **0.041** | |  |  |  |  |  |  | medium - low | 0.548 | | 0.036 | 1.817 | | 0.108 | | |  |  |
|  | Jaccard | high - medium | 0.350 | 0.025 | 1.219 | 0.500 | |  |  | Jaccard | | | | high - medium | 0.426 | | 0.031 | 1.380 | | **0.017** | | |  |  |
|  |  | high - low | 0.509 | 0.031 | 1.764 | **0.002** | |  |  |  |  |  |  | high - low | 0.792 | | 0.051 | 2.584 | | **0.002** | | |  |  |
|  |  | medium - low | 0.463 | 0.032 | 1.648 | **0.002** | |  |  |  |  |  |  | medium - low | 0.654 | | 0.041 | 2.099 | | **0.002** | | |  |  |
|  | Unweighted UniFrac | high - medium | 0.109 | 0.022 | 1.112 | 0.231 | |  |  | Unweighted UniFrac | | | | high - medium | 0.305 | | 0.032 | 1.407 | | **0.040** | | |  |  |
|  |  | high - low | 0.168 | 0.030 | 1.714 | **0.027** | |  |  |  |  |  |  | high - low | 0.532 | | 0.049 | 2.481 | | **0.002** | | |  |  |
|  |  | medium - low | 0.139 | 0.030 | 1.502 | **0.036** | |  |  |  |  |  |  | medium - low | 0.461 | | 0.041 | 2.086 | | **0.002** | | |  |  |
|  | Weighted UniFrac | high - medium | 0.032 | 0.006 | 0.308 | 0.961 | |  |  | Weighted UniFrac | | | | high - medium | 0.198 | | 0.019 | 0.865 | | 0.518 | | |  |  |
|  |  | high - low | 0.145 | 0.026 | 1.502 | 0.284 | |  |  |  |  |  |  | high - low | 0.272 | | 0.027 | 1.332 | | 0.300 | | |  |  |
|  |  | medium - low | 0.123 | 0.028 | 1.432 | 0.284 | |  |  |  |  |  |  | medium - low | 0.330 | | 0.028 | 1.391 | | 0.300 | | |  |  |
|  |  |  |  |  |  |  | |  |  |  | | | |  |  | |  |  | |  | | |  |  |
|  | Land-use Type | |  |  |  |  | |  |  | Land-use Type | | | | |  | |  |  | |  | | |  |  |
|  | **Dissimilarity Index** | **Variable** | **Sum of squares** | **R2** | ***F*** | ***Corrected p*** | |  |  | **Dissimilarity Index** | | | | **Variable** | **Sum of squares** | | **R2** | ***F*** | | ***Corrected p*** | | |  |  |
|  | Bray-Curtis | Mowed pasture - meadow | 0.378 | 0.029 | 1.289 | 0.097 | |  |  | Bray-Curtis | | | | Mowed pasture - meadow | 0.283 | | 0.025 | 0.958 | | 0.446 | | |  |  |
|  |  | Mowed pasture - pasture | 0.446 | 0.023 | 1.476 | **0.046** | |  |  |  |  |  |  | Mowed pasture - pasture | 0.615 | | 0.036 | 2.155 | | **0.045** | | |  |  |
|  |  | Meadow - pasture | 0.447 | 0.031 | 1.496 | **0.046** | |  |  |  |  |  |  | Meadow - pasture | 0.369 | | 0.027 | 1.286 | | 0.336 | | |  |  |
|  | Jaccard | Mowed pasture - meadow | 0.332 | 0.027 | 1.175 | 0.074 | |  |  | Jaccard | | | | Mowed pasture - meadow | 0.442 | | 0.037 | 1.439 | | **0.012** | | |  |  |
|  |  | Mowed pasture - pasture | 0.479 | 0.026 | 1.667 | **0.003** | |  |  |  |  |  |  | Mowed pasture - pasture | 0.707 | | 0.038 | 2.279 | | **0.002** | | |  |  |
|  |  | Meadow - pasture | 0.450 | 0.032 | 1.566 | **0.005** | |  |  |  |  |  |  | Meadow - pasture | 0.600 | | 0.040 | 1.925 | | **0.002** | | |  |  |
|  | Unweighted UniFrac | Mowed pasture - meadow | 0.119 | 0.023 | 1.261 | 0.099 | |  |  | Unweighted UniFrac | | | | Mowed pasture - meadow | 0.340 | | 0.041 | 1.566 | | **0.016** | | |  |  |
|  |  | Mowed pasture - pasture | 0.169 | 0.027 | 1.748 | **0.018** | |  |  |  |  |  |  | Mowed pasture - pasture | 0.575 | | 0.045 | 2.674 | | **0.002** | | |  |  |
|  |  | Meadow - pasture | 0.148 | 0.032 | 1.536 | **0.030** | |  |  |  |  |  |  | Meadow - pasture | 0.4114 | | 0.039 | 1.890 | | **0.002** | | |  |  |
|  | Weighted UniFrac | Mowed pasture - meadow | 0.074 | 0.018 | 0.780 | 0.562 | |  |  | Weighted UniFrac | | | | Mowed pasture - meadow | 0.186 | | 0.021 | 0.785 | | 0.584 | | |  |  |
|  |  | Mowed pasture - pasture | 0.121 | 0.019 | 1.278 | 0.366 | |  |  |  |  |  |  | Mowed pasture - pasture | 0.372 | | 0.029 | 1.700 | | 0.240 | | |  |  |
|  |  | Meadow - pasture | 0.157 | 0.034 | 1.674 | 0.348 | |  |  |  |  |  |  | Meadow - pasture | 0.204 | | 0.019 | 0.932 | | 0.548 | | |  |  |

**Table S7:** Generalized linear mixed effects model results for (A) bacterial (V4-5) and (B) fungal (ITS2) gut microbiota alpha-diversity in *Myodes glareolus*; p-values corrected by Benjamin-Hochberg correction. Bold text indicates statistical significance.

| **A** | Season | |  |  |  |  | | | |  | **B** | Season | | |  | |  | |  | |  |
| --- | --- | --- | --- | --- | --- | --- | --- | --- | --- | --- | --- | --- | --- | --- | --- | --- | --- | --- | --- | --- | --- |
|  | **Diversity Index** | **Variable** | **Estimate** | **Std. Error** | ***Corrected p*** | |  |  | **Diversity Index** | | | | **Variable** | **Estimate** | | **Std. Error** | | ***Corrected p*** | |  |  |
|  | Shannon | Intercept | 1.766 | 0.043 | **<0.001** | |  |  | Shannon | | | | Intercept | 0.990 | | 0.102 | | **<0.001** | |  |  |
|  |  | November | -0.131 | 0.044 | **0.005** | |  |  |  |  |  |  | November | 0.266 | | 0.108 | | 0.169 | |  |  |
|  |  | September | 0.009 | 0.045 | 0.851 | |  |  |  |  |  |  | September | 0.139 | | 0.120 | | 0.532 | |  |  |
|  | Pielou´s Evenness | Intercept | -0.407 | 0.038 | **<0.001** | |  |  | Pielou´s Evenness | | | | Intercept | -0.657 | | 0.075 | | **<0-001** | |  |  |
|  |  | November | -0.061 | 0.038 | 0.170 | |  |  |  |  |  |  | November | 0.114 | | 0.080 | | 0.835 | |  |  |
|  |  | September | 0.026 | 0.041 | 0.532 | |  |  |  |  |  |  | September | 0.031 | | 0.089 | | 0.835 | |  |  |
|  | Faith´s PD | Intercept | 3.413 | 0.034 | **<0.001** | |  |  | Faith´s PD | | | | Intercept | 2.443 | | 0.098 | | **<0.001** | |  |  |
|  |  | November | -0.145 | 0.046 | **0.002** | |  |  |  |  |  |  | November | 0.422 | | 0.102 | | **<0.001** | |  |  |
|  |  | September | -0.031 | 0.049 | 0.531 | |  |  |  |  |  |  | September | 0.242 | | 0.113 | | **0.033** | |  |  |
|  | Number of features | Intercept | 6.099 | 0.068 | **<0.001** | |  |  | Number of features | | | | Intercept | 3.785 | | 0.008 | | **<0.001** | |  |  |
|  |  | November | -0.374 | 0.093 | **<0.001** | |  |  |  |  |  |  | November | 0.445 | | 0.008 | | **<0.001** | |  |  |
|  |  | September | -0.085 | 0.101 | 0.397 | |  |  |  |  |  |  | September | 0.305 | | 0.008 | | **<0.001** | |  |  |
|  |  |  |  |  |  | |  |  |  | | | |  |  | |  | |  | |  |  |
|  | Land-use intensity | |  |  |  | |  |  | Land-use intensity | | | | |  | |  | |  | |  |  |
|  | **Diversity Index** | **Variable** | **Estimate** | **Std. Error** | ***Corrected p*** | |  |  | **Diversity Index** | | | | **Variable** | **Estimate** | | **Std. Error** | | ***Corrected p*** | |  |  |
|  | Shannon | Intercept | 1.682 | 0.070 | **<0.001** | |  |  | Shannon | | | | Intercept | 1.117 | | 0.014 | | **<0.001** | |  |  |
|  |  | Land-use intensity | 0.033 | 0.052 | 0.520 | |  |  |  |  |  |  | Land-use intensity | 0.032 | | 0.014 | | **0.029** | |  |  |
|  | Pielou´s Evenness | Intercept | -0.439 | 0.064 | **<0.001** | |  |  | Pielou´s Evenness | | | | Intercept | -0.609 | | 0.080 | | **<0.001** | |  |  |
|  |  | Land-use intensity | 0.015 | 0.046 | 0.740 | |  |  |  |  |  |  | Land-use intensity | 0.013 | | 0.060 | | 0.583 | |  |  |
|  | Faith´s PD | Intercept | 3.306 | 0.047 | **<0.001** | |  |  | Faith´s PD | | | | Intercept | 2.603 | | 0.117 | | **<0.001** | |  |  |
|  |  | Land-use intensity | 0.039 | 0.036 | 0.269 | |  |  |  |  |  |  | Land-use intensity | 0.092 | | 0.088 | | 0.296 | |  |  |
|  | Number of features | Intercept | 5.869 | 0.084 | **<0.001** | |  |  | Number of features | | | | Intercept | 3.978 | | 0.152 | | **<0.001** | |  |  |
|  |  | Land-use intensity | 0.069 | 0.064 | 0.272 | |  |  |  |  |  |  | Land-use intensity | 0.090 | | 0.114 | | 0.427 | |  |  |
|  |  |  |  |  |  | |  |  |  | | | |  |  | |  | |  | |  |  |
|  | Forest type | |  |  |  | |  |  | Forest type | | | | |  | |  | |  | |  |  |
|  | **Diversity Index** | **Variable** | **Estimate** | **Std. Error** | ***Corrected p*** | |  |  | **Diversity Index** | | | | **Variable** | **Estimate** | | **Std. Error** | | ***Corrected p*** | |  |  |
|  | Shannon | Intercept | 1.762 | 0.068 | **<0.001** | |  |  | Shannon | | | | Intercept | 1.143 | | 0.132 | | **<0.001** | |  |  |
|  |  | Old-managed beech | -0.021 | 0.094 | 0.825 | |  |  |  |  |  |  | Old-managed beech | -0.093 | | 0.181 | | 0.965 | |  |  |
|  |  | Unmanaged beech | -0.086 | 0.102 | 0.628 | |  |  |  |  |  |  | Unmanaged beech | 0.056 | | 0.199 | | 0.967 | |  |  |
|  |  | Young-managed beech | -0.068 | 0.094 | 0.628 | |  |  |  |  |  |  | Young-managed beech | 0.088 | | 0.181 | | 0.965 | |  |  |
|  | Pielou´s Evenness | Intercept | -0.399 | 0.061 | **<0.001** | |  |  | Pielou´s Evenness | | | | Intercept | -0.612 | | 0.095 | | **<0.001** | |  |  |
|  |  | Old-managed beech | 0.001 | 0.085 | 0.987 | |  |  |  |  |  |  | Old-managed beech | -0.065 | | 0.132 | | 0.884 | |  |  |
|  |  | Unmanaged beech | -0.054 | 0.092 | 0.801 | |  |  |  |  |  |  | Unmanaged beech | 0.071 | | 0.144 | | 0.884 | |  |  |
|  |  | Young-managed beech | -0.044 | 0.085 | 0.600 | |  |  |  |  |  |  | Young-managed beech | 0.071 | | 0.131 | | 0.884 | |  |  |
|  | Faith´s PD | Intercept | 3.402 | 0.047 | **<0.001** | |  |  | Faith´s PD | | | | Intercept | 2.741 | | 0.116 | | **<0.001** | |  |  |
|  |  | Old-managed beech | -0.048 | 0.063 | 0.444 | |  |  |  |  |  |  | Old-managed beech | -0.129 | | 0.156 | | 0.725 | |  |  |
|  |  | Unmanaged beech  Young-managed beech | -0.096  -0.065 | 0.069  0.064 | 0.328  0.413 | |  |  |  |  |  |  | Unmanaged beech  Young-managed beech | -0.062  0.055 | | 0.173  0.156 | | 0.725 0.725 | |  |  |
|  |  |  |  |  |  |  |  |  |  |  |  |  |  |  |  |  |  |  |  |  |  |
|  | Number of features | Intercept | 6.039 | 0.092 | **<0.001** | |  |  | Number of features | | | | Intercept | 4.084 | | 0.144 | | **<0.001** | |  |  |
|  |  | Old-managed beech | -0.084 | 0.119 | 0.482 | |  |  |  |  |  |  | Old-managed beech | -0.111 | | 0.193 | | 0.749 | |  |  |
|  |  | Unmanaged beech | -0.154 | 0.134 | 0.386 | |  |  |  |  |  |  | Unmanaged beech | -0.065 | | 0.214 | | 0.759 | |  |  |
|  |  | Young-managed beech | -0.131 | 0.124 | 0.386 | |  |  |  |  |  |  | Young-managed beech | 0.144 | | 0.194 | | 0.749 | |  |  |

**Table S8:** Pairwise PERMANOVA results for (A) bacterial (V4-5) and (B) fungal (ITS2) gut microbiota beta-diversity within *Myodes glareolus*; p-values corrected by Benjamin-Hochberg correction. Bold text indicates statistical significance.

| **A** | Season | |  |  |  |  |  | | | |  | **B** | Season | | |  | |  |  | | |  | |  | |
| --- | --- | --- | --- | --- | --- | --- | --- | --- | --- | --- | --- | --- | --- | --- | --- | --- | --- | --- | --- | --- | --- | --- | --- | --- | --- |
|  | **Dissimilarity Index** | **Variable** | **Sum of squares** | **R2** | ***F*** | ***Corrected p*** | |  |  | **Dissimilarity Index** | | | | **Variable** | **Sum of squares** | | **R2** | ***F*** | | ***Corrected p*** | | |  |  |  |
|  | Bray-Curtis | June - September | 0.769 | 0.055 | 2.323 | **0.003** | |  |  | Bray-Curtis | | | | June - September | 1.574 | | 0.148 | 5.600 | | **0.002** | | |  |  |  |
|  |  | June - November | 1.073 | 0.065 | 3.186 | **0.003** | |  |  |  |  |  |  | June - November | 1.409 | | 0.0917 | 4.141 | | **0.002** | | |  |  |  |
|  |  | September - November | 0.603 | 0.043 | 1.866 | **0.006** | |  |  |  |  |  |  | September - November | 0.771 | | 0.054 | 2.347 | | **0.012** | | |  |  |  |
|  | Jaccard | June - September | 0.585 | 0.050 | 2.128 | **0.001** | |  |  | Jaccard | | | | June - September | 0.715 | | 0.064 | 2.215 | | **0.001** | | |  |  |  |
|  |  | June - November | 0.833 | 0.058 | 2.819 | **0.001** | |  |  |  |  |  |  | June - November | 0.810 | | 0.056 | 2.466 | | **0.001** | | |  |  |  |
|  |  | September - November | 0.634 | 0.048 | 2.119 | **0.001** | |  |  |  |  |  |  | September - November | 0.803 | | 0.059 | 2.572 | | **0.001** | | |  |  |  |
|  | Unweighted UniFrac | June - September | 0.192 | 0.053 | 2.234 | **0.002** | |  |  | Unweighted UniFrac | | | | June - September | 0.525 | | 0.067 | 2.305 | | **0.001** | | |  |  |  |
|  |  | June - November | 0.377 | 0.077 | 3.843 | **0.002** | |  |  |  |  |  |  | June - November | 0.683 | | 0.067 | 2.977 | | **0.001** | | |  |  |  |
|  |  | September - November | 0.203 | 0.047 | 2.082 | **0.002** | |  |  |  |  |  |  | September - November | 0.691 | | 0.071 | 3.144 | | **0.001** | | |  |  |  |
|  | Weighted UniFrac | June - September | 0.36 | 0.095 | 4.201 | **0.001** | |  |  | Weighted UniFrac | | | | June - September | 1.334 | | 0.097 | 4.427 | | **0.008** | | |  |  |  |
|  |  | June - November | 0.476 | 0.115 | 6.000 | **0.001** | |  |  |  |  |  |  | June - November | 1.574 | | 0.157 | 5.960 | | **0.003** | | |  |  |  |
|  |  | September - November | 0.415 | 0.106 | 4.967 | **0.001** | |  |  |  |  |  |  | September - November | 1.027 | | 0.078 | 3.482 | | **0.010** | | |  |  |  |
|  |  |  |  |  |  |  | |  |  |  | | | |  |  | |  |  | |  | | |  |  |  |
|  | Land-use intensity | |  |  |  |  | |  |  | Land-use intensity | | | | |  | |  |  | |  | | |  |  |  |
|  | **Dissimilarity Index** | **Variable** | **Sum of squares** | **R2** | ***F*** | ***Corrected p*** | |  |  | **Dissimilarity Index** | | | | **Variable** | **Sum of squares** | | **R2** | ***F*** | | ***Corrected p*** | | |  |  |  |
|  | Bray-Curtis | high - medium | 0.559 | 0.035 | 1.615 | **0.015** | |  |  | Bray-Curtis | | | | high - medium | 0.305 | | 0.020 | 0.861 | | 0.822 | | |  |  |  |
|  |  | high - low | 0.559 | 0.043 | 1.654 | **0.017** | |  |  |  |  |  |  | high - low | 0.313 | | 0.027 | 0.900 | | 0.822 | | |  |  |  |
|  |  | medium - low | 0.348 | 0.021 | 1.029 | 0.405 | |  |  |  |  |  |  | medium - low | 0.232 | | 0.015 | 0.657 | | 0.822 | | |  |  |  |
|  | Jaccard | high - medium | 0.433 | 0.031 | 1.429 | **0.015** | |  |  | Jaccard | | | | high - medium | 0.548 | | 0.038 | 1.646 | | **0.006** | | |  |  |  |
|  |  | high - low | 0.361 | 0.033 | 1.246 | **0.032** | |  |  |  |  |  |  | high - low | 0.406 | | 0.036 | 1.227 | | 0.110 | | |  |  |  |
|  |  | medium - low | 0.392 | 0.027 | 1.289 | **0.032** | |  |  |  |  |  |  | medium - low | 0.328 | | 0.023 | 0.980 | | 0.504 | | |  |  |  |
|  | Unweighted UniFrac | high - medium | 0.16 | 0.036 | 1.624 | **0.027** | |  |  | Unweighted UniFrac | | | | high - medium | 0.404 | | 0.040 | 1.709 | | **0.021** | | |  |  |  |
|  |  | high - low | 0.145 | 0.039 | 1.512 | **0.027** | |  |  |  |  |  |  | high - low | 0.318 | | 0.040 | 1.356 | | **0.038** | | |  |  |  |
|  |  | medium - low | 0.139 | 0.029 | 1.419 | **0.040** | |  |  |  |  |  |  | medium - low | 0.221 | | 0.022 | 0.924 | | 0.578 | | |  |  |  |
|  | Weighted UniFrac | high - medium | 0.147 | 0.033 | 1.509 | 0.206 | |  |  | Weighted UniFrac | | | | high - medium | 0.140 | | 0.010 | 0.420 | | 0.891 | | |  |  |  |
|  |  | high - low | 0.143 | 0.046 | 1.815 | 0.183 | |  |  |  |  |  |  | high - low | 0.170 | | 0.016 | 0.528 | | 0.891 | | |  |  |  |
|  |  | medium - low | 0.101 | 0.022 | 1.054 | 0.365 | |  |  |  |  |  |  | medium - low | 0.233 | | 0.017 | 0.720 | | 0.891 | | |  |  |  |
|  |  |  |  |  |  |  | |  |  |  | | | |  |  | |  |  | |  |  | | | |  |
|  | Forest Type | |  |  |  |  | |  |  | Forest Type | | | | |  | |  |  | |  |  | | | |  |
|  | **Dissimilarity Index** | **Variable** | **Sum of squares** | **R2** | ***F*** | ***Corrected p*** | |  |  | **Dissimilarity Index** | | | | **Variable** | **Sum of squares** | | **R2** | ***F*** | | ***Corrected p*** | | |  |  |  |
|  | Bray-Curtis | Coniferous - Young-managed-beech | 0.404 | 0.035 | 1.145 | 0.323 | |  |  | Bray-Curtis | | | | Coniferous - Young-managed-beech | 0.386 | | 0.036 | 10.868 | | 0.486 | | |  |  |  |
|  |  | Coniferous - Old-managed-beech | 0.542 | 0.045 | 1.604 | **0.048** | |  |  |  |  |  |  | Coniferous - Old-managed-beech | 0.416 | | 0.040 | 12.418 | | 0.434 | | |  |  |  |
|  |  | Coniferous - Unmanaged-beech | 0.565 | 0.059 | 1.653 | **0.048** | |  |  |  |  |  |  | Coniferous - Unmanaged-beech | 0.318 | | 0.037 | 00.048.884 | | 0.508 | | |  |  |  |
|  |  | Young-managed-beech - Old-managed-beech | 0.351 | 0.027 | 1.029 | 0.382 | |  |  |  |  |  |  | Young-managed-beech - Old-managed-beech | 0.536 | | 0.046 | 15.80.38293 | | 0.434 | | |  |  |  |
|  |  | Young-managed-beech - Old-managed-beech | 0.368 | 0.035 | 1.069 | 0.369 | |  |  |  |  |  |  | Young-managed-beech - Old-managed-beech | 0.359 | | 0.037 | 0.9970.369 | | 0.508 | | |  |  |  |
|  |  | Old-managed-beech - Unmanaged-beech | 0.391 | 0.035 | 1.185 | 0.323 | |  |  |  |  |  |  | Old-managed-beech - Unmanaged-beech | 0.444 | | 0.046 | 1.3160.323 | | 0.434 | | |  |  |  |
|  | Jaccard | Coniferous - Young-managed-beech | 0.360 | 0.037 | 1.207 | 0.094 | |  |  | Jaccard | | | | Coniferous - Young-managed-beech | 0.610 | | 0.062 | 19.021 | | **0.006** | | |  |  |  |
|  |  | Coniferous - Old-managed-beech | 0.367 | 0.035 | 1.230 | 0.094 | |  |  |  |  |  |  | Coniferous - Old-managed-beech | 0.492 | | 0.046 | 1.452 | | **0.012** | | |  |  |  |
|  |  | Coniferous - Unmanaged-beech | 0.368 | 0.046 | 1.250 | 0.094 | |  |  |  |  |  |  | Coniferous - Unmanaged-beech | 0.481 | | 0.060 | 14.791 | | **0.012** | | |  |  |  |
|  |  | Young-managed-beech - Old-managed-beech | 0.334 | 0.028 | 1.099 | 0.186 | |  |  |  |  |  |  | Young-managed-beech - Old-managed-beech | 0.443 | | 0.039 | 1.335 | | **0.049** | | |  |  |  |
|  |  | Young-managed-beech - Old-managed-beech | 0.339 | 0.037 | 1.112 | 0.186 | |  |  |  |  |  |  | Young-managed-beech - Old-managed-beech | 0.418 | | 0.048 | 13.164 | | **0.038** | | |  |  |  |
|  |  | Old-managed-beech - Unmanaged-beech | 0.359 | 0.036 | 1.191 | 0.143 | |  |  |  |  |  |  | Old-managed-beech - Unmanaged-beech | 0.405 | | 0.042 | 11.973 | | 0.112 | | |  |  |  |
|  | Unweighted UniFrac | Coniferous - Young-managed-beech | 0.118 | 0.038 | 1.224 | 0.156 | |  |  | Unweighted UniFrac | | | | Coniferous - Young-managed-beech | 0.404 | | 0.057 | 17.646 | | **0.036** | | |  |  |  |
|  |  | Coniferous - Old-managed-beech | 0.136 | 0.039 | 1.390 | 0.116 | |  |  |  |  |  |  | Coniferous - Old-managed-beech | 0.355 | | 0.047 | 14.681 | | 0.260 | | |  |  |  |
|  |  | Coniferous - Unmanaged-beech | 0.155 | 0.056 | 1.556 | 0.116 | |  |  |  |  |  |  | Coniferous - Unmanaged-beech | 0.325 | | 0.058 | 14.143 | | 0.074 | | |  |  |  |
|  |  | Young-managed-beech - Old-managed-beech | 0.113 | 0.031 | 1.116 | 0.192 | |  |  |  |  |  |  | Young-managed-beech - Old-managed-beech | 0.343 | | 0.042 | 14.405 | | 0.074 | | |  |  |  |
|  |  | Young-managed-beech - Old-managed-beech | 0.118 | 0.040 | 1.212 | 0.156 | |  |  |  |  |  |  | Young-managed-beech - Old-managed-beech | 0.261 | | 0.042 | 11.481 | | 0.260 | | |  |  |  |
|  |  | Old-managed-beech - Unmanaged-beech | 0.138 | 0.042 | 1.396 | 0.116 | |  |  |  |  |  |  | Old-managed-beech - Unmanaged-beech | 0.256 | | 0.038 | 1.061 | | 0.342 | | |  |  |  |
|  | Weighted UniFrac | Coniferous - Young-managed-beech | 0.129 | 0.040 | 1.293 | 0.411 | |  |  | Weighted UniFrac | | | | Coniferous - Young-managed-beech | 0.272 | | 0.028 | 0.824 | | 0.851 | | |  |  |  |
|  |  | Coniferous - Old-managed-beech | 0.146 | 0.046 | 1.646 | 0.411 | |  |  |  |  |  |  | Coniferous - Old-managed-beech | 0.189 | | 0.019 | 0.574 | | 0.851 | | |  |  |  |
|  |  | Coniferous - Unmanaged-beech | 0.106 | 0.048 | 1.319 | 0.411 | |  |  |  |  |  |  | Coniferous - Unmanaged-beech | 0.151 | | 0.019 | 0.435 | | 0.857 | | |  |  |  |
|  |  | Young-managed-beech - Old-managed-beech | 0.067 | 0.017 | 0.669 | 0.780 | |  |  |  |  |  |  | Young-managed-beech - Old-managed-beech | 0.532 | | 0.050 | 17.242 | | 0.762 | | |  |  |  |
|  |  | Young-managed-beech - Old-managed-beech | 0.117 | 0.039 | 1.194 | 0.411 | |  |  |  |  |  |  | Young-managed-beech - Old-managed-beech | 0.224 | | 0.026 | 0.705 | | 0.851 | | |  |  |  |
|  |  | Old-managed-beech - Unmanaged-beech | 0.046 | 0.016 | 0.538 | 0.839 | |  |  |  |  |  |  | Old-managed-beech - Unmanaged-beech | 0.353 | | 0.039 | 11.086 | | 0.851 | | |  |  |  |

**Table S9:** ANCOM-BC results for differentially abundant (A) bacterial (V4-5) and (B) fungal (ITS2) gut microbiota families between the two species. Positive Log fold change values indicate higher abundance compared to the reference, negative values indicate a lower abundance. TRUE indicates a significant difference in abundance between the species, FALSE indicates no significant difference. Bold text indicates statistical significance.

|  | Family | **Intercept** | **Log fold change** | ***M.arv* - *M. gla*** | **Log fold change** |
| --- | --- | --- | --- | --- | --- |
| **A** | **Pasteurellaceae** | FALSE | -0.05 | TRUE | 1.69 |
|  | Rs-E47_termite_group | TRUE | 0.45 | FALSE | -0.02 |
|  | **Rikenellaceae** | TRUE | -0.43 | TRUE | 1.03 |
|  | **Prevotellaceae** | FALSE | -0.24 | TRUE | -2.15 |
|  | Muribaculaceae | TRUE | 4.55 | FALSE | -0.07 |
|  | **Marinifilaceae** | TRUE | -0.52 | TRUE | 1.85 |
|  | **Burkholderiales Order** | TRUE | -2.89 | TRUE | 2.41 |
|  | Helicobacteraceae | TRUE | -3.31 | FALSE | 0.75 |
|  | **Desulfovibrionaceae** | TRUE | 3.60 | TRUE | 1.79 |
|  | Cyanobacteriia Class | FALSE | 0.28 | FALSE | 0.07 |
|  | **Coriobacteriales Order** | FALSE | -0.04 | TRUE | -2.42 |
|  | **Eggerthellaceae** | TRUE | 3.33 | TRUE | 0 |
|  | Spirochaetaceae | TRUE | 2.10 | FALSE | 0.59 |
|  | Propionibacteriaceae | TRUE | -3.17 | FALSE | 0.17 |
|  | **RF39** | TRUE | 1.94 | TRUE | 1.17 |
|  | Erysipelotrichaceae | TRUE | 2.87 | FALSE | -0.21 |
|  | Streptococcaceae | FALSE | 0.17 | FALSE | -0.15 |
|  | Staphylococcaceae | TRUE | -2.29 | FALSE | 0.74 |
|  | **Peptococcaceae** | TRUE | -3.46 | TRUE | 1.25 |
|  | **Lachnospiraceae** | TRUE | 3.63 | TRUE | 1.07 |
|  | **Anaerovoracaceae** | TRUE | -0.79 | TRUE | 0.73 |
|  | Christensenellaceae | TRUE | 3.04 | FALSE | -0.04 |
|  | **Clostridia_UCG-014** | TRUE | 0.79 | TRUE | 1.54 |
|  | **Clostridia_vadinBB60_group** | TRUE | 0.70 | TRUE | 1.34 |
|  | **Ruminococcaceae** | TRUE | 2.21 | TRUE | 0.74 |
|  | [Eubacterium]_coprostanoligenes_group | TRUE | -2.17 | FALSE | 0.46 |
|  | UCG-010 | TRUE | -2.40 | FALSE | -0.23 |
|  | **Oscillospiraceae** | TRUE | 2.79 | TRUE | 1.09 |
|  | Butyricicoccaceae | TRUE | -1.88 | FALSE | 0.31 |
|  | Monoglobaceae | TRUE | -1.58 | FALSE | 0.10 |
|  | **Coriobacteriales_Incertae_Sedis** | TRUE | 0.27 | TRUE | -1.77 |
|  | **Lactobacillaceae** | TRUE | 2.34 | TRUE | 2.59 |
|  | Rhizobiaceae | TRUE | -1.05 | FALSE | 0.74 |
|  |  |  |  |  |  |
| **B** | **Fungal Family** | **Intercept** | **Log fold change** | ***M.arv* - *M. gla*** | **Log fold change** |
|  | **Agaricomycetes Class** | true | -1.52 | true | 0.98 |
|  | Tricholomataceae | false | -0.38 | false | 0.88 |
|  | Psathyrellaceae | true | 1.18 | false | 0.47 |
|  | Strophariaceae | true | -0.67 | false | 0.86 |
|  | Filobasidiaceae | false | -0.14 | false | -0.56 |
|  | **Hypocreales_fam_Incertae_sedis** | true | 1.15 | true | -1.4 |
|  | Sporidiobolaceae | false | -0.26 | false | -0.32 |
|  | Cordycipitaceae | false | -0.13 | false | 0.5 |
|  | Bulleribasidiaceae | true | 2.38 | false | -0.82 |
|  | Mucoraceae | true | 6 | false | 0.19 |
|  | Ascomycota Phylum | false | 0.51 | false | 0.44 |
|  | Malasseziaceae | true | 2 | false | -1 |
|  | **Cystobasidiomycetes_fam_Incertae_sedis** | true | 0.8 | true | -1.38 |
|  | Saccharomycetales_fam_Incertae_sedis | true | 1.66 | false | 0.2 |
|  | Microascaceae | true | 2.39 | false | -0.37 |
|  | **Herpotrichiellaceae** | false | 0.09 | true | 1.08 |
|  | Cyphellophoraceae | true | 1.08 | false | -0.31 |
|  | Sordariales Order | false | 0.43 | false | 0.3 |
|  | Sordariomycetes Class | false | -0.28 | false | -0.06 |
|  | **Leotiomycetes Class** | true | 0.68 | true | 2.91 |
|  | **Pleosporales Order** | true | 4.16 | true | -2.71 |
|  | **Sporormiaceae** | true | 2.84 | true | -2.55 |
|  | Dothideomycetes Class | true | -0.52 | false | 0.15 |
|  | **Saccotheciaceae** | false | 0.16 | true | 1.85 |
|  | **Pleosporales_fam_Incertae_sedis** | true | 1.2 | true | -2.08 |
|  | Aspergillaceae | true | 4.15 | false | 0.84 |
|  | Helotiaceae | false | -0.34 | false | 0.47 |
|  | **Phaeosphaeriaceae** | true | 1.39 | true | -2.04 |
|  | Pleosporaceae | true | -0.65 | false | 0.15 |
|  | Didymellaceae | true | 1.24 | false | -0.32 |
|  | **Helotiales Order** | true | -1.02 | true | 1.36 |
|  | **Thelebolaceae** | true | 4.33 | true | -2.53 |
|  | **Pseudeurotiaceae** | true | -1.43 | true | 1.94 |
|  | Cladosporiaceae | true | 4.41 | false | -0.72 |
|  | **Clavicipitaceae** | true | -0.99 | true | 1.57 |
|  | **Podosporaceae** | true | 2.29 | true | -2.9 |
|  | **Stachybotryaceae** | true | 1.4 | true | -2.13 |
|  | Nectriaceae | true | 2.7 | false | 0.05 |
|  | **Hypocreaceae** | false | -0.43 | true | 2.55 |
|  | Bionectriaceae | false | 0.37 | false | 0.46 |
|  | Myrmecridiaceae | false | -0.37 | false | -0.33 |
|  | Chaetomiaceae | false | -0.3 | false | 0.01 |
|  | **Neoschizotheciaceae** | true | 3.3 | true | -3.1 |

**Table S10:** ANCOM-BC results for differentially abundant (A) bacterial (V4-5) and (B) fungal (ITS2) gut microbiota in *Microtus arvalis* between land-use intensities and sesons. Positive Log fold change values indicate higher abundance compared to the reference, negative values indicate a lower abundance. TRUE indicates a significant difference in abundance between the species, FALSE indicates no significant difference. Bold text indicates statistical significance.

|  | Family | **Season** | | | | | | **Land-use intensity** | | | | | | **Land-use type** | | | | | |
| --- | --- | --- | --- | --- | --- | --- | --- | --- | --- | --- | --- | --- | --- | --- | --- | --- | --- | --- | --- |
|  |  | **Inter-cept** | **LFC** | **Jun - Nov** | **LFC** | **Jun - Sep** | **LFC** | **Inter-cept** | **LFC** | **high - low** | **LFC** | **high - medium** | **LFC** | **Inter-cept** | **LFC** | **MP - P** | **LFC** | **MP - M** | **LFC** |
| **A** | **Pasteurellaceae** | FALSE | -1.24 | FALSE | 1.43 | TRUE | 2.1 | FALSE | 0.68 | FALSE | -0.76 | FALSE | 0.64 | FALSE | -0.09 | FALSE | -0.09 | FALSE | 1.07 |
|  | Gammaproteobacteria | TRUE | -3.59 | FALSE | 0.93 | FALSE | 0.99 | TRUE | -2.3 | FALSE | -0.24 | FALSE | 0.12 | TRUE | -2.82 | FALSE | -0.19 | FALSE | 0.17 |
|  | Rs-E47 termite group | TRUE | 0.65 | FALSE | -0.34 | FALSE | -0.06 | TRUE | 1.27 | FALSE | -0.22 | FALSE | -0.36 | TRUE | 0.63 | FALSE | -0.15 | FALSE | -0.01 |
|  | Rikenellaceae | FALSE | -0.15 | FALSE | -0.29 | FALSE | -0.36 | FALSE | -0.09 | FALSE | 0.25 | FALSE | 0.69 | FALSE | -0.43 | FALSE | -0.06 | FALSE | 0.77 |
|  | Prevotellaceae | FALSE | -0.73 | FALSE | 1.2 | FALSE | 0.41 | FALSE | 0.61 | FALSE | -0.46 | FALSE | -0.2 | FALSE | 0.4 | FALSE | -0.83 | FALSE | -0.92 |
|  | **Muribaculaceae** | TRUE | 4.62 | FALSE | 0 | FALSE | -0.04 | TRUE | 5.4 | FALSE | -0.57 | FALSE | -0.06 | TRUE | 4.89 | TRUE | -0.56 | FALSE | 0.07 |
|  | Marinifilaceae | FALSE | -0.16 | FALSE | -0.41 | FALSE | -0.44 | FALSE | -0.05 | FALSE | 0.06 | FALSE | 0.5 | FALSE | -0.47 | FALSE | -0.01 | FALSE | 0.39 |
|  | Burkholderiales | TRUE | -2.35 | FALSE | -0.43 | FALSE | -0.93 | TRUE | -2.4 | FALSE | 0.62 | FALSE | -0.34 | TRUE | -3.1 | FALSE | 0.7 | FALSE | 0.08 |
|  | Helicobacteraceae | TRUE | -2.75 | FALSE | -0.73 | FALSE | -0.7 | TRUE | -1.82 | FALSE | -1.21 | FALSE | -1.5 | TRUE | -3.02 | FALSE | -0.14 | FALSE | -0.64 |
|  | Desulfovibrionaceae | TRUE | 3.89 | FALSE | -0.42 | FALSE | -0.27 | TRUE | 4.28 | FALSE | -0.04 | FALSE | -0.14 | TRUE | 3.75 | FALSE | 0 | FALSE | -0.25 |
|  | **Cyanobacteriia** | FALSE | 0.18 | FALSE | -0.09 | FALSE | 0.51 | FALSE | 0.62 | FALSE | 0.68 | FALSE | 0.15 | FALSE | -0.13 | TRUE | 1.12 | FALSE | 0.18 |
|  | **Gastranaerophilales** | TRUE | -3.96 | TRUE | 2.53 | TRUE | 2.27 | TRUE | -2.21 | FALSE | 0.57 | FALSE | 0.99 | TRUE | -2.5 | FALSE | 0.11 | FALSE | 1.2 |
|  | Coriobacteriales | FALSE | 0.01 | FALSE | 0.21 | FALSE | -0.16 | TRUE | 0.48 | FALSE | 0.18 | FALSE | 0.16 | FALSE | 0 | FALSE | 0.07 | FALSE | 0.23 |
|  | Eggerthellaceae | TRUE | 3.63 | FALSE | -0.2 | FALSE | -0.47 | TRUE | 4.01 | FALSE | 0.04 | FALSE | -0.24 | TRUE | 3.43 | FALSE | 0.08 | FALSE | -0.15 |
|  | Spirochaetaceae | TRUE | 2.67 | FALSE | -0.72 | FALSE | -0.73 | TRUE | 2.86 | FALSE | -0.24 | FALSE | -0.12 | TRUE | 2.46 | FALSE | -0.41 | FALSE | -0.37 |
|  | Propionibacteriaceae | TRUE | -3.07 | FALSE | -0.2 | FALSE | 0.06 | TRUE | -2.45 | FALSE | -0.28 | FALSE | 0.04 | TRUE | -3.36 | FALSE | 0.28 | FALSE | 1.03 |
|  | RF39 | TRUE | 2.12 | FALSE | -0.12 | FALSE | -0.22 | TRUE | 2.57 | FALSE | -0.05 | FALSE | 0.06 | TRUE | 1.88 | FALSE | 0.15 | FALSE | 0.57 |
|  | **Erysipelotrichaceae** | TRUE | 1.7 | TRUE | 2.85 | FALSE | 0.79 | TRUE | 3.89 | FALSE | -0.94 | FALSE | -0.17 | TRUE | 3.33 | FALSE | -0.72 | FALSE | -0.14 |
|  | Streptococcaceae | FALSE | -0.13 | FALSE | 0.55 | FALSE | 0.49 | TRUE | 1.03 | FALSE | -0.53 | FALSE | -0.12 | FALSE | 0.15 | FALSE | 0.12 | FALSE | 0.46 |
|  | Staphylococcaceae | TRUE | -2.2 | FALSE | -0.56 | FALSE | 0.4 | TRUE | -1.32 | FALSE | -0.47 | FALSE | -0.63 | TRUE | -2.1 | FALSE | -0.24 | FALSE | 0.13 |
|  | Peptococcaceae | TRUE | -3.04 | FALSE | -0.42 | FALSE | -0.61 | TRUE | -2.95 | FALSE | 0.36 | FALSE | -0.04 | TRUE | -3.67 | FALSE | 0.7 | FALSE | 0.07 |
|  | **Lachnospiraceae** | TRUE | 3.86 | FALSE | 0.17 | TRUE | -0.62 | TRUE | 4.24 | FALSE | -0.05 | FALSE | 0.14 | TRUE | 3.83 | FALSE | -0.18 | FALSE | -0.03 |
|  | Anaerovoracaceae | TRUE | -0.72 | FALSE | 0.68 | FALSE | -0.64 | FALSE | -0.36 | FALSE | 0.21 | FALSE | 0.45 | TRUE | -0.91 | FALSE | 0.21 | FALSE | 0.8 |
|  | **Christensenellaceae** | TRUE | 2.42 | TRUE | 1.59 | FALSE | 0.42 | TRUE | 4.27 | FALSE | -1.05 | FALSE | -0.78 | TRUE | 3.43 | FALSE | -0.49 | FALSE | -0.37 |
|  | Clostridia UCG-014 | TRUE | 0.86 | FALSE | 0.19 | FALSE | -0.18 | TRUE | 1.31 | FALSE | 0.18 | FALSE | 0.15 | TRUE | 0.78 | FALSE | 0.11 | FALSE | 0.4 |
|  | Clostridia vadinBB60 group | TRUE | 0.75 | FALSE | 0.39 | FALSE | -0.32 | TRUE | 1.41 | FALSE | -0.22 | FALSE | -0.02 | TRUE | 0.85 | FALSE | -0.11 | FALSE | 0.02 |
|  | Ruminococcaceae | TRUE | 2.21 | FALSE | 0.3 | FALSE | -0.09 | TRUE | 2.91 | FALSE | -0.19 | FALSE | 0.01 | TRUE | 2.41 | FALSE | -0.13 | FALSE | -0.12 |
|  | [Eubacterium] coprostanoligenes group | TRUE | -2.21 | FALSE | 0.62 | FALSE | -0.28 | TRUE | -1.56 | FALSE | -0.13 | FALSE | 0.21 | TRUE | -2.24 | FALSE | 0.02 | FALSE | 0.95 |
|  | UCG-010 | TRUE | -2.89 | FALSE | 1.17 | FALSE | 0.45 | TRUE | -1.49 | FALSE | -0.72 | FALSE | -0.05 | TRUE | -2.17 | FALSE | -0.54 | FALSE | 0.72 |
|  | **Oscillospiraceae** | TRUE | 3.1 | FALSE | -0.09 | TRUE | -0.61 | TRUE | 3.37 | FALSE | 0.11 | FALSE | 0.03 | TRUE | 2.84 | FALSE | 0.14 | FALSE | -0.03 |
|  | Butyricicoccaceae | TRUE | -1.56 | FALSE | -0.62 | FALSE | -0.16 | TRUE | -1.33 | FALSE | 0.27 | FALSE | -0.06 | TRUE | -2.12 | FALSE | 0.65 | FALSE | 0.37 |
|  | Monoglobaceae | TRUE | -1.69 | FALSE | 0.39 | FALSE | 0.12 | TRUE | -1.23 | FALSE | 0.37 | FALSE | 0.53 | TRUE | -1.65 | FALSE | 0.24 | FALSE | 0.44 |
|  | Coriobacteriales Incertae Sedis | FALSE | -0.02 | FALSE | 0.61 | FALSE | 0.39 | TRUE | 0.98 | FALSE | -0.09 | FALSE | -0.19 | TRUE | 0.51 | FALSE | -0.21 | FALSE | -0.22 |
|  | Lactobacillaceae | TRUE | 2.27 | FALSE | 0.74 | FALSE | -0.31 | TRUE | 3.38 | FALSE | -1.1 | FALSE | -0.03 | TRUE | 2.62 | FALSE | -0.65 | FALSE | 0.71 |
|  | Enterococcaceae | TRUE | -2.52 | FALSE | -0.48 | FALSE | 0.5 | TRUE | -1.73 | FALSE | -0.4 | FALSE | -0.17 | TRUE | -2.77 | FALSE | 0.16 | FALSE | 1.4 |
|  | **Rhizobiaceae** | FALSE | 0.21 | TRUE | -2.63 | FALSE | -0.92 | FALSE | -0.07 | FALSE | -0.39 | FALSE | -0.77 | FALSE | -0.68 | FALSE | -0.13 | FALSE | -1.14 |
|  |  |  |  |  |  |  |  |  |  |  |  |  |  |  |  |  |  |  |  |
| **B** |  |  |  |  |  |  |  |  |  |  |  |  |  |  |  |  |  |  |  |
|  | Agaricomycetes Class | TRUE | -0.9 | FALSE | -0.04 | FALSE | -0.97 | TRUE | -1.02 | FALSE | 0.24 | FALSE | -0.32 | TRUE | -1.49 | FALSE | 0.39 | FALSE | -0.23 |
|  | **Tricholomataceae** | FALSE | -1.22 | TRUE | 3.8 | FALSE | -0.48 | FALSE | 0.37 | FALSE | -0.11 | FALSE | -0.11 | FALSE | -0.1 | FALSE | -0.03 | FALSE | 0.53 |
|  | **Psathyrellaceae** | FALSE | 1.07 | TRUE | 1.79 | FALSE | -0.01 | TRUE | 2.43 | FALSE | -0.29 | FALSE | -0.96 | TRUE | 1.91 | FALSE | -0.13 | FALSE | -0.79 |
|  | **Strophariaceae** | FALSE | -0.57 | TRUE | 1.46 | FALSE | -0.86 | FALSE | 0.3 | FALSE | -0.68 | FALSE | -0.36 | FALSE | -0.49 | FALSE | 0.05 | FALSE | 0.35 |
|  | Filobasidiaceae | TRUE | 1.36 | FALSE | -1.56 | FALSE | -1.33 | FALSE | 0.56 | FALSE | 0.09 | FALSE | -0.24 | FALSE | -0.08 | FALSE | 0.42 | FALSE | 0.4 |
|  | Cunninghamellaceae | FALSE | -0.93 | FALSE | 0.02 | FALSE | -0.4 | FALSE | -0.45 | FALSE | -0.46 | FALSE | -0.55 | TRUE | -0.96 | FALSE | -0.32 | FALSE | -0.05 |
|  | Hypocreales_fam_Incertae_sedis | TRUE | 1.36 | FALSE | 0.7 | FALSE | 0.22 | TRUE | 1.92 | FALSE | -0.14 | FALSE | 0.36 | TRUE | 1.68 | FALSE | -0.28 | FALSE | 0.59 |
|  | Rhynchogastremataceae | FALSE | -0.03 | FALSE | -0.55 | FALSE | -1.49 | FALSE | -0.14 | FALSE | -0.55 | FALSE | -0.64 | TRUE | -1.13 | FALSE | 0.47 | FALSE | 0.16 |
|  | Sporidiobolaceae | FALSE | 0.63 | FALSE | -0.75 | FALSE | -0.57 | FALSE | 0.73 | FALSE | -0.36 | FALSE | -0.64 | FALSE | -0.12 | FALSE | 0.2 | FALSE | 0.51 |
|  | **Cordycipitaceae** | TRUE | -1.51 | TRUE | 2.31 | TRUE | 2.29 | FALSE | 0.38 | FALSE | 0.64 | FALSE | -0.3 | FALSE | -0.36 | FALSE | 0.72 | FALSE | 1.24 |
|  | **Leucosporidiaceae** | FALSE | 0.79 | TRUE | -1.54 | TRUE | -2.08 | FALSE | -0.31 | FALSE | 0.11 | FALSE | -0.23 | FALSE | -0.33 | FALSE | -0.38 | FALSE | -0.77 |
|  | Bulleribasidiaceae | TRUE | 3.73 | FALSE | -0.26 | FALSE | -1.56 | TRUE | 3.34 | FALSE | -0.26 | FALSE | 0.17 | TRUE | 2.99 | FALSE | -0.27 | FALSE | 0.6 |
|  | Mucoraceae | TRUE | 6.98 | FALSE | 0.94 | FALSE | -1.24 | TRUE | 7.48 | FALSE | -0.61 | FALSE | -0.37 | TRUE | 6.71 | FALSE | -0.16 | FALSE | 0.87 |
|  | **Ascomycota Phylum** | FALSE | 0.08 | TRUE | 2.27 | FALSE | 0.17 | TRUE | 1.89 | FALSE | -0.72 | FALSE | -1.08 | TRUE | 1.89 | FALSE | -1.32 | TRUE | -1.63 |
|  | Malasseziaceae | TRUE | 2.31 | FALSE | 0.34 | FALSE | 0.23 | TRUE | 2.41 | FALSE | 0.31 | FALSE | 0.84 | TRUE | 2.1 | FALSE | 0.19 | FALSE | 1.54 |
|  | Kondoaceae | FALSE | -0.57 | FALSE | 0.89 | FALSE | -0.31 | FALSE | 0.01 | FALSE | -0.34 | FALSE | 0.12 | FALSE | -0.63 | FALSE | 0.04 | FALSE | 1.1 |
|  | Cystobasidiomycetes_fam_Incertae_sedis | FALSE | 0.73 | FALSE | 1.28 | FALSE | 0.25 | TRUE | 1.67 | FALSE | -0.16 | FALSE | -0.08 | TRUE | 1.03 | FALSE | 0.16 | FALSE | 0.81 |
|  | **Saccharomycetales_fam_Incertae_sedis** | FALSE | 1.26 | TRUE | 1.87 | FALSE | 0.84 | TRUE | 1.79 | FALSE | 0.79 | TRUE | 1.53 | TRUE | 1.97 | FALSE | -0.01 | TRUE | 1.51 |
|  | Debaryomycetaceae | FALSE | -0.29 | FALSE | 0.92 | FALSE | 0.06 | FALSE | 0.89 | FALSE | -1.26 | FALSE | -0.12 | FALSE | 0.32 | FALSE | -0.72 | FALSE | 0.32 |
|  | **Cryptococcaceae** | FALSE | 1.21 | FALSE | -1.43 | TRUE | -2.13 | FALSE | 1.11 | FALSE | -1.4 | FALSE | -1.5 | FALSE | 0.44 | FALSE | -0.75 | FALSE | -1.6 |
|  | Microascaceae | TRUE | 2.96 | FALSE | 0.84 | FALSE | -0.59 | TRUE | 3.6 | FALSE | -0.24 | FALSE | -0.6 | TRUE | 3.26 | FALSE | -0.5 | FALSE | -0.11 |
|  | Gymnoascaceae | FALSE | -1.02 | FALSE | 0.91 | FALSE | -0.13 | FALSE | -0.63 | FALSE | 0.33 | FALSE | 0.15 | FALSE | -0.78 | FALSE | -0.23 | FALSE | 0.59 |
|  | Herpotrichiellaceae | FALSE | 0.72 | FALSE | 0.36 | FALSE | -0.84 | FALSE | 0.47 | FALSE | 0.75 | FALSE | 0.11 | FALSE | 0.22 | FALSE | 0.66 | FALSE | -0.29 |
|  | **Cyphellophoraceae** | TRUE | 2.02 | FALSE | -0.21 | FALSE | -0.76 | TRUE | 2.52 | FALSE | -0.71 | FALSE | -1.04 | TRUE | 2.5 | TRUE | -1.25 | FALSE | -1.63 |
|  | Sordariales Order | FALSE | 0.5 | FALSE | 1.54 | FALSE | -0.34 | TRUE | 1.28 | FALSE | 0.22 | FALSE | -0.47 | FALSE | 0.02 | FALSE | 1.31 | FALSE | 1.38 |
|  | Sordariomycetes Class | FALSE | -0.23 | FALSE | 0.98 | FALSE | -0.04 | FALSE | 0.47 | FALSE | 0.12 | FALSE | -0.42 | FALSE | 0.17 | FALSE | -0.15 | FALSE | -0.17 |
|  | **Pyronemataceae** | FALSE | -0.93 | TRUE | 4.38 | FALSE | -1.23 | FALSE | 0.67 | FALSE | -0.3 | FALSE | -0.11 | FALSE | 0.78 | FALSE | -1.11 | FALSE | -0.31 |
|  | **Leotiomycetes Class** | TRUE | 0.88 | TRUE | 1.95 | FALSE | -1.08 | FALSE | 1.18 | FALSE | 0.49 | FALSE | 0.3 | TRUE | 1.29 | FALSE | -0.37 | FALSE | 0.17 |
|  | **Pleosporales Order** | TRUE | 5.7 | FALSE | -0.21 | TRUE | -1.56 | TRUE | 5.75 | FALSE | -0.78 | FALSE | -0.55 | TRUE | 5.05 | FALSE | -0.25 | FALSE | 0.12 |
|  | **Sporormiaceae** | FALSE | 1.54 | TRUE | 3.33 | FALSE | 2.22 | TRUE | 4.35 | FALSE | 0.48 | TRUE | -1.93 | TRUE | 3.25 | FALSE | 1.16 | FALSE | -0.92 |
|  | Dothideomycetes Class | FALSE | -0.15 | FALSE | -0.05 | FALSE | 0.06 | FALSE | -0.07 | FALSE | 0.24 | FALSE | 0.32 | FALSE | -0.1 | FALSE | -0.34 | FALSE | 0.36 |
|  | **Saccotheciaceae** | TRUE | 1.77 | FALSE | -1.4 | FALSE | -1.68 | FALSE | -0.18 | TRUE | 1.94 | FALSE | 0.88 | FALSE | -0.41 | FALSE | 1.44 | FALSE | 1.39 |
|  | **Pleosporales_fam_Incertae_sedis** | FALSE | 0.62 | TRUE | 2.56 | FALSE | 0.56 | TRUE | 2.2 | FALSE | -0.13 | FALSE | -0.24 | TRUE | 1.44 | FALSE | 0.3 | FALSE | 0.91 |
|  | **Aspergillaceae** | TRUE | 4.06 | TRUE | 1.69 | FALSE | 1.05 | TRUE | 5.2 | FALSE | 0.23 | FALSE | 0.31 | TRUE | 4.7 | FALSE | 0.31 | TRUE | 1.13 |
|  | Helotiaceae | FALSE | -0.02 | FALSE | 1.31 | FALSE | -1.18 | FALSE | 0.69 | FALSE | 0.02 | FALSE | -1.35 | FALSE | 0.17 | FALSE | 0.04 | FALSE | -1.18 |
|  | **Phaeosphaeriaceae** | TRUE | 2.9 | TRUE | -1.32 | FALSE | -1.14 | TRUE | 1.57 | FALSE | 1.11 | FALSE | 0.69 | TRUE | 1.45 | FALSE | 0.5 | FALSE | 1.17 |
|  | Pleosporaceae | FALSE | -0.1 | FALSE | -0.32 | FALSE | -0.2 | FALSE | -0.27 | FALSE | 0.9 | FALSE | -0.39 | FALSE | -0.75 | FALSE | 0.79 | FALSE | 0.11 |
|  | Didymellaceae | TRUE | 1.8 | FALSE | 0.25 | FALSE | -0.33 | TRUE | 2.56 | FALSE | -0.44 | FALSE | -1.13 | TRUE | 1.82 | FALSE | -0.06 | FALSE | -0.4 |
|  | **Helotiales Order** | TRUE | -1.63 | TRUE | 2.35 | FALSE | 0.01 | FALSE | -0.5 | FALSE | 0.72 | FALSE | -0.81 | FALSE | -0.97 | FALSE | 0.61 | FALSE | -0.54 |
|  | **Thelebolaceae** | TRUE | 4.08 | TRUE | 3.2 | FALSE | 0.12 | TRUE | 5.78 | FALSE | 0.07 | FALSE | -0.64 | TRUE | 4.64 | FALSE | 0.72 | FALSE | 1.67 |
|  | Saccharomycetaceae | FALSE | 0.15 | FALSE | 0.27 | FALSE | 0.34 | FALSE | 1.81 | FALSE | -2.24 | FALSE | -0.88 | FALSE | 1.22 | FALSE | -1.55 | FALSE | -0.78 |
|  | Cladosporiaceae | TRUE | 5.34 | FALSE | 0.07 | FALSE | -0.02 | TRUE | 5.61 | FALSE | -0.1 | FALSE | 0.19 | TRUE | 5.39 | FALSE | -0.35 | FALSE | 0.45 |
|  | Clavicipitaceae | TRUE | -1.34 | FALSE | 1.07 | FALSE | 0.58 | FALSE | -0.58 | FALSE | 0.77 | FALSE | -0.53 | TRUE | -1.29 | FALSE | 0.88 | FALSE | 0.62 |
|  | **Podosporaceae** | TRUE | 2.06 | FALSE | 1.31 | FALSE | 1.1 | TRUE | 2.94 | FALSE | 1.57 | FALSE | -0.92 | TRUE | 1.97 | TRUE | 2.11 | FALSE | -0.12 |
|  | **Stachybotryaceae** | TRUE | 3.14 | TRUE | -2.49 | FALSE | -0.62 | TRUE | 2.21 | FALSE | 0.34 | FALSE | -0.38 | TRUE | 1.91 | FALSE | 0.32 | FALSE | -0.8 |
|  | **Plectosphaerellaceae** | TRUE | -1.52 | TRUE | 1.87 | FALSE | -0.36 | FALSE | -0.83 | FALSE | 0.1 | FALSE | 0.36 | FALSE | -0.91 | FALSE | -0.49 | FALSE | 0.74 |
|  | Nectriaceae | TRUE | 3.29 | FALSE | 0.51 | FALSE | 0.02 | TRUE | 3.74 | FALSE | -0.1 | FALSE | 0.22 | TRUE | 3.26 | FALSE | 0.03 | FALSE | 0.89 |
|  | **Hypocreaceae** | FALSE | -0.87 | TRUE | 1.63 | FALSE | 0.44 | FALSE | -0.14 | TRUE | 1.44 | FALSE | -0.74 | TRUE | -0.95 | TRUE | 1.7 | FALSE | 0.1 |
|  | Bionectriaceae | FALSE | 0.09 | FALSE | 1.47 | FALSE | 0.5 | TRUE | 1.32 | FALSE | -0.37 | FALSE | -0.2 | FALSE | 0.67 | FALSE | 0.23 | FALSE | 0.09 |
|  | **Myrmecridiaceae** | FALSE | 0.73 | FALSE | -0.4 | FALSE | -1.44 | FALSE | -0.22 | TRUE | 1.61 | FALSE | -0.39 | TRUE | -0.9 | TRUE | 1.94 | FALSE | -0.3 |
|  | **Lasiosphaeriaceae** | TRUE | -1.59 | TRUE | 2 | FALSE | 0.33 | FALSE | -0.03 | FALSE | -0.24 | FALSE | -1 | TRUE | -1.48 | FALSE | 1.44 | FALSE | 0.25 |
|  | **Chaetomiaceae** | FALSE | -0.09 | FALSE | 0.19 | FALSE | 0.19 | FALSE | -1.03 | TRUE | 2.56 | FALSE | 1.12 | FALSE | -1.03 | FALSE | 1.65 | FALSE | 1.36 |
|  | **Neoschizotheciaceae** | TRUE | 3.18 | TRUE | 2.64 | FALSE | -0.13 | TRUE | 3.97 | FALSE | 1.32 | FALSE | -0.29 | TRUE | 3.56 | FALSE | 0.74 | FALSE | 0.86 |
|  | Sordariaceae | FALSE | -0.05 | FALSE | -0.26 | FALSE | 0.45 | FALSE | 0.26 | FALSE | 0.57 | FALSE | -0.6 | FALSE | -0.41 | FALSE | 0.7 | FALSE | 0.3 |
|  | **Chaetosphaeriaceae** | TRUE | 1.52 | TRUE | -2.49 | FALSE | -0.88 | FALSE | 0.4 | FALSE | 0.83 | FALSE | -0.72 | FALSE | -0.39 | FALSE | 1.52 | FALSE | -0.79 |

**Table S11:** ANCOM-BC results for differentially abundant (A) bacterial (V4-5) and (B) fungal (ITS2) gut microbiota in *Myodes glareolus* between land-use intensities and sesons. Positive Log fold change values indicate higher abundance compared to the reference, negative values indicate a lower abundance. TRUE indicates a significant difference in abundance between the species, FALSE indicates no significant difference. Bold text indicates statistical significance.

|  | Family | **Season** | | | | | | **Land-use intensity** | | | | | | **Land-use type** | | | | | | |  | |  | |
| --- | --- | --- | --- | --- | --- | --- | --- | --- | --- | --- | --- | --- | --- | --- | --- | --- | --- | --- | --- | --- | --- | --- | --- | --- |
|  |  | **Inter-cept** | **LFC** | **Jun - Nov** | **LFC** | **Jun - Sep** | **LFC** | **Inter-cept** | **LFC** | **high - low** | **LFC** | **high - medium** | **LFC** | **Inter-cept** | **LFC** | **C - Old-MB** | **LFC** | **C - Un-MB** | **LFC** | **C - Young-MB** | | **LFC** | |  |
| **A** | Pasteurellaceae | TRUE | 1.44 | FALSE | 1.29 | FALSE | 1.12 | TRUE | 1.55 | FALSE | 0.47 | FALSE | 0.66 | TRUE | 1.04 | FALSE | 1.13 | FALSE | -0.5 | FALSE | | 1.06 | |  |
|  | Rs-E47 termite group | FALSE | 0.96 | FALSE | -0.28 | FALSE | 0.62 | FALSE | 0.8 | FALSE | -0.8 | FALSE | 0.52 | FALSE | 0.56 | FALSE | -0.13 | FALSE | -1.3 | FALSE | | 0.4 | |  |
|  | Rikenellaceae | TRUE | 1.56 | FALSE | -0.63 | FALSE | -0.43 | TRUE | 1.25 | FALSE | -0.62 | FALSE | -0.3 | TRUE | 0.95 | FALSE | -0.53 | FALSE | -0.75 | FALSE | | -0.38 | |  |
|  | Prevotellaceae | TRUE | -2.22 | FALSE | 0.48 | FALSE | 0.92 | TRUE | -1.85 | FALSE | -0.88 | FALSE | 0.18 | TRUE | -2.19 | FALSE | -0.6 | FALSE | -1.22 | FALSE | | 0.6 | |  |
|  | Muribaculaceae | TRUE | 5.16 | FALSE | -0.58 | FALSE | 0.47 | TRUE | 4.82 | FALSE | -0.49 | FALSE | 0.35 | TRUE | 4.37 | FALSE | -0.21 | FALSE | -0.13 | FALSE | | 0.49 | |  |
|  | Marinifilaceae | TRUE | 1.66 | FALSE | 0.33 | FALSE | 0.54 | TRUE | 1.86 | FALSE | -0.31 | FALSE | -0.23 | TRUE | 1.46 | FALSE | -0.11 | FALSE | -0.46 | FALSE | | -0.25 | |  |
|  | Oxalobacteraceae | TRUE | -2.01 | FALSE | 1.12 | FALSE | 0.16 | TRUE | -2.04 | FALSE | 0.6 | FALSE | 0.09 | TRUE | -2.29 | FALSE | 0.31 | FALSE | 0.16 | FALSE | | -0.19 | |  |
|  | **Burkholderiales** | FALSE | -0.98 | FALSE | 1.58 | TRUE | 1.84 | FALSE | -0.2 | FALSE | 0.35 | FALSE | -0.13 | FALSE | -0.63 | FALSE | 0.33 | FALSE | -0.21 | FALSE | | 0.11 | |  |
|  | **Xanthobacteraceae** | TRUE | -2.42 | TRUE | 1.63 | FALSE | 0.45 | TRUE | -2.3 | FALSE | 0.35 | FALSE | 0.57 | TRUE | -2.8 | FALSE | 0.63 | FALSE | -0.07 | FALSE | | 0.97 | |  |
|  | **Helicobacteraceae** | TRUE | -2.28 | FALSE | 0.48 | FALSE | 0.5 | TRUE | -2.86 | FALSE | 0.64 | FALSE | 1.03 | TRUE | -3.16 | FALSE | 1.06 | FALSE | 0.63 | FALSE | | 0.31 | |  |
|  | Desulfovibrionaceae | TRUE | 6.38 | FALSE | -0.38 | FALSE | -0.87 | TRUE | 5.87 | FALSE | -0.53 | FALSE | 0.04 | TRUE | 5.47 | FALSE | -0.34 | FALSE | 0.14 | FALSE | | -0.23 | |  |
|  | Cyanobacteriia | TRUE | 1.25 | FALSE | -0.25 | FALSE | -0.7 | TRUE | 1.2 | FALSE | -0.97 | FALSE | -0.51 | FALSE | 0.78 | FALSE | -0.25 | FALSE | -1.6 | FALSE | | -0.37 | |  |
|  | **Eggerthellaceae** | TRUE | 4.72 | FALSE | -0.14 | TRUE | -0.98 | TRUE | 4.33 | FALSE | -0.3 | FALSE | -0.28 | TRUE | 3.95 | FALSE | -0.15 | FALSE | -0.42 | FALSE | | -0.37 | |  |
|  | Spirochaetaceae | TRUE | 4.11 | FALSE | -1.61 | FALSE | -0.72 | TRUE | 3.5 | FALSE | -1.87 | FALSE | 0.29 | TRUE | 3.15 | FALSE | -1.58 | FALSE | -0.52 | FALSE | | 0.33 | |  |
|  | RF39 | TRUE | 3.81 | FALSE | -0.09 | FALSE | -0.22 | TRUE | 3.47 | FALSE | 0.18 | FALSE | -0.2 | TRUE | 3 | FALSE | 0.1 | FALSE | 0.15 | FALSE | | -0.05 | |  |
|  | Erysipelotrichaceae | TRUE | 2.66 | FALSE | 1.05 | FALSE | 0.76 | TRUE | 2.81 | FALSE | 0.1 | FALSE | 0.39 | TRUE | 2.66 | FALSE | -0.05 | FALSE | -0.45 | FALSE | | 0.17 | |  |
|  | Streptococcaceae | FALSE | 0.14 | FALSE | 1 | FALSE | 0.38 | FALSE | 0.4 | FALSE | -0.18 | FALSE | 0.03 | FALSE | 0.07 | FALSE | -0.2 | FALSE | -0.33 | FALSE | | 0.08 | |  |
|  | **Staphylococcaceae** | TRUE | -1.1 | FALSE | -0.04 | FALSE | 0.57 | FALSE | -0.27 | FALSE | -1.51 | FALSE | -1.14 | FALSE | -0.72 | TRUE | -1.64 | FALSE | -1.07 | FALSE | | -0.66 | |  |
|  | Peptococcaceae | TRUE | -1.83 | FALSE | 0.63 | FALSE | -0.02 | TRUE | -1.98 | FALSE | 0.1 | FALSE | 0.2 | TRUE | -2.6 | FALSE | 0.15 | FALSE | 0.72 | FALSE | | 0.57 | |  |
|  | Lachnospiraceae | TRUE | 5.36 | FALSE | 0.01 | FALSE | -0.21 | TRUE | 5.02 | FALSE | -0.06 | FALSE | 0.08 | TRUE | 4.62 | FALSE | -0.01 | FALSE | 0.1 | FALSE | | 0.01 | |  |
|  | Anaerovoracaceae | FALSE | 0.01 | FALSE | 0.51 | FALSE | 1.2 | FALSE | 0.45 | FALSE | 0 | FALSE | -0.42 | FALSE | -0.09 | FALSE | -0.19 | FALSE | -0.29 | FALSE | | 0.34 | |  |
|  | **Christensenellaceae** | TRUE | 2.67 | TRUE | 2.18 | FALSE | 0.41 | TRUE | 3.19 | FALSE | -0.15 | FALSE | 0.45 | TRUE | 2.95 | FALSE | -0.61 | FALSE | 0.25 | FALSE | | 0.47 | |  |
|  | Clostridia UCG-014 | TRUE | 2.73 | FALSE | 0.6 | FALSE | -0.06 | TRUE | 2.65 | FALSE | 0.14 | FALSE | -0.06 | TRUE | 2.22 | FALSE | 0.26 | FALSE | 0.14 | FALSE | | -0.22 | |  |
|  | Clostridia vadinBB60 group | TRUE | 2.69 | FALSE | 0.07 | FALSE | -0.24 | TRUE | 2.42 | FALSE | 0.04 | FALSE | -0.15 | TRUE | 2.21 | FALSE | -0.12 | FALSE | -0.31 | FALSE | | -0.51 | |  |
|  | Ruminococcaceae | TRUE | 3.5 | FALSE | 0.09 | FALSE | 0.09 | TRUE | 3.34 | FALSE | 0.03 | FALSE | -0.14 | TRUE | 3.08 | FALSE | -0.29 | FALSE | 0.06 | FALSE | | -0.4 | |  |
|  | [Eubacterium] coprostanoligenes group | TRUE | -0.96 | FALSE | -0.36 | FALSE | -0.02 | TRUE | -1.38 | FALSE | 0.2 | FALSE | -0.15 | TRUE | -1.91 | FALSE | 0.12 | FALSE | 0.2 | FALSE | | 0.22 | |  |
|  | Oscillospiraceae | TRUE | 4.35 | FALSE | 0.29 | FALSE | 0.09 | TRUE | 4.19 | FALSE | 0.12 | FALSE | -0.03 | TRUE | 3.75 | FALSE | 0.16 | FALSE | 0.02 | FALSE | | 0.05 | |  |
|  | **Butyricicoccaceae** | FALSE | -0.27 | FALSE | -0.89 | FALSE | -1.29 | FALSE | -0.05 | TRUE | -1.91 | TRUE | -1.39 | FALSE | -0.64 | FALSE | -1.43 | FALSE | -1.65 | FALSE | | -0.82 | |  |
|  | Monoglobaceae | TRUE | -1.18 | FALSE | 0.1 | FALSE | 0.94 | TRUE | -1.31 | FALSE | 0.28 | FALSE | 0.19 | TRUE | -1.81 | FALSE | 0.26 | FALSE | 0.14 | FALSE | | 0.59 | |  |
|  | Coriobacteriales Incertae Sedis | FALSE | 0.16 | TRUE | -1.53 | TRUE | -1.72 | FALSE | -0.2 | TRUE | -2.41 | FALSE | -0.51 | FALSE | -0.61 | FALSE | -1.73 | FALSE | -1.61 | FALSE | | -0.37 | |  |
|  | **Lactobacillaceae** | TRUE | 4.5 | TRUE | 1.95 | FALSE | 1.09 | TRUE | 4.28 | FALSE | 1.09 | FALSE | 1.56 | TRUE | 3.78 | FALSE | 1.44 | FALSE | 1.52 | FALSE | | 1.29 | |  |
|  | **Rhizobiaceae** | TRUE | 1.56 | TRUE | -2.24 | FALSE | -1.51 | FALSE | -0.05 | FALSE | -0.24 | FALSE | 0.35 | FALSE | -0.55 | FALSE | -0.11 | FALSE | 0.64 | FALSE | | 0.32 | |  |
|  |  |  |  |  |  |  |  |  |  |  |  |  |  |  |  |  |  |  |  |  | |  | |  |
| **B** |  |  |  |  |  |  |  |  |  |  |  |  |  |  |  |  |  |  |  |  | |  | |  |
|  | Agaricomycetes Class | FALSE | -0.7 | FALSE | 0.05 | FALSE | 0.05 | TRUE | -1.67 | FALSE | 0.05 | FALSE | 0.5 | TRUE | -1.73 | FALSE | 0.46 | FALSE | 0.06 | FALSE | | 0.98 | |  |
|  | **Tricholomataceae** | FALSE | -0.39 | TRUE | 2.83 | FALSE | -0.83 | FALSE | 0.08 | FALSE | -0.27 | FALSE | -0.36 | FALSE | 0.41 | FALSE | 0.14 | FALSE | -1.19 | FALSE | | -0.87 | |  |
|  | Psathyrellaceae | TRUE | 1.71 | FALSE | 0.36 | FALSE | -0.13 | FALSE | 0.75 | FALSE | 0.21 | FALSE | 0.55 | FALSE | 0.21 | FALSE | 0.86 | FALSE | 0.75 | FALSE | | 2.04 | |  |
|  | Strophariaceae | FALSE | -0.35 | FALSE | 1.65 | FALSE | -0.63 | FALSE | -0.65 | FALSE | 0.5 | FALSE | -0.17 | FALSE | -0.39 | FALSE | 0.37 | FALSE | -0.41 | FALSE | | -0.3 | |  |
|  | Filobasidiaceae | FALSE | -0.33 | FALSE | -0.4 | FALSE | -1.17 | TRUE | -1.58 | FALSE | -0.4 | FALSE | 0.18 | TRUE | -1.5 | FALSE | -0.02 | FALSE | -0.2 | FALSE | | 0.24 | |  |
|  | Omphalotaceae | FALSE | -0.48 | FALSE | 0.81 | FALSE | -0.74 | TRUE | -1.5 | FALSE | 0.61 | FALSE | 0.48 | TRUE | -1.37 | FALSE | 0.35 | FALSE | 1.51 | FALSE | | 0.04 | |  |
|  | Hypocreales_fam_Incertae_sedis | FALSE | -0.15 | FALSE | -0.34 | FALSE | -0.15 | TRUE | -1.51 | FALSE | 0.7 | FALSE | 0.42 | TRUE | -1.54 | FALSE | 0.22 | FALSE | 0.27 | FALSE | | 1.55 | |  |
|  | Sporidiobolaceae | FALSE | -0.02 | FALSE | -0.89 | FALSE | -0.92 | FALSE | -0.86 | FALSE | -1.19 | FALSE | -0.56 | FALSE | -0.72 | FALSE | -0.88 | FALSE | -1.42 | FALSE | | -0.19 | |  |
|  | Cordycipitaceae | FALSE | -0.02 | FALSE | 0.23 | FALSE | 0.85 | FALSE | -0.61 | FALSE | 0.01 | FALSE | 0.37 | FALSE | -0.28 | FALSE | -0.01 | FALSE | -0.2 | FALSE | | 0.04 | |  |
|  | Bulleribasidiaceae | TRUE | 2.26 | FALSE | 0.12 | FALSE | -1.95 | FALSE | 1.33 | FALSE | -1.01 | FALSE | -0.13 | FALSE | 1.21 | FALSE | -0.57 | FALSE | 0.3 | FALSE | | 0.09 | |  |
|  | Mucoraceae | TRUE | 7.75 | FALSE | -1.88 | FALSE | -0.34 | TRUE | 6.12 | FALSE | 0.09 | FALSE | -0.23 | TRUE | 6.38 | FALSE | 0.32 | FALSE | -0.62 | FALSE | | -0.6 | |  |
|  | **Ascomycota Phylum** | FALSE | 0.67 | FALSE | 0.74 | FALSE | 0.19 | FALSE | 1.14 | FALSE | -0.96 | FALSE | -1.32 | FALSE | 1.35 | FALSE | -1.18 | TRUE | -1.95 | FALSE | | -0.71 | |  |
|  | Malasseziaceae | TRUE | 1.05 | FALSE | 0.27 | FALSE | 0.57 | FALSE | 0.72 | FALSE | -0.41 | FALSE | -0.11 | FALSE | 0.95 | FALSE | -0.19 | FALSE | -0.24 | FALSE | | -0.51 | |  |
|  | Erythrobasidiaceae | FALSE | 0.02 | FALSE | 1.02 | FALSE | -1.17 | FALSE | -0.1 | FALSE | -0.8 | FALSE | -0.73 | FALSE | -0.01 | FALSE | -0.79 | FALSE | -0.43 | FALSE | | -0.62 | |  |
|  | **Cystobasidiaceae** | FALSE | 0.3 | FALSE | -0.49 | TRUE | -1.72 | TRUE | -1.33 | FALSE | -0.11 | FALSE | 0.38 | FALSE | -1.23 | FALSE | 0.36 | FALSE | 0.54 | FALSE | | -0.11 | |  |
|  | Saccharomycetales_fam_Incertae_sedis | TRUE | 1.19 | FALSE | 1.47 | FALSE | 0.67 | FALSE | 1.27 | FALSE | -0.14 | FALSE | 0.06 | FALSE | 1.61 | FALSE | -0.49 | FALSE | 0.19 | FALSE | | -0.34 | |  |
|  | Cystobasidiomycetes_fam_Incertae_sedis | FALSE | -0.21 | FALSE | -0.37 | FALSE | -1.13 | TRUE | -1.04 | FALSE | -0.69 | FALSE | -0.53 | TRUE | -1.25 | FALSE | -0.17 | FALSE | -0.27 | FALSE | | 0.08 | |  |
|  | Microascaceae | TRUE | 2.02 | FALSE | -0.13 | FALSE | 0.99 | TRUE | 1.37 | FALSE | 0.07 | FALSE | 0.17 | FALSE | 1.17 | FALSE | -0.1 | FALSE | 0.75 | FALSE | | 1.16 | |  |
|  | Trichosporonaceae | FALSE | 0.1 | FALSE | -0.06 | FALSE | -0.48 | FALSE | -0.79 | FALSE | -0.11 | FALSE | -0.04 | FALSE | -0.92 | FALSE | -0.2 | FALSE | 0.5 | FALSE | | 0.66 | |  |
|  | Herpotrichiellaceae | TRUE | 1.37 | FALSE | -0.04 | FALSE | -0.32 | FALSE | 0.69 | FALSE | -0.46 | FALSE | -0.17 | FALSE | 0.54 | FALSE | -0.2 | FALSE | -0.48 | FALSE | | 0.82 | |  |
|  | Capnodiales_fam_Incertae_sedis | FALSE | 0.13 | FALSE | -0.38 | FALSE | -1.06 | FALSE | -0.89 | FALSE | -0.58 | FALSE | -0.14 | FALSE | -0.64 | FALSE | -0.41 | FALSE | -0.88 | FALSE | | -0.18 | |  |
|  | **Chaetothyriales Order** | FALSE | 0.61 | FALSE | -0.09 | FALSE | -0.96 | FALSE | 0.58 | FALSE | -1.5 | FALSE | -1.43 | FALSE | 1.02 | TRUE | -1.65 | TRUE | -2.51 | FALSE | | -1.37 | |  |
|  | **Cyphellophoraceae** | TRUE | 1.39 | FALSE | -0.12 | TRUE | -1.73 | FALSE | 0.58 | FALSE | -0.72 | FALSE | -0.69 | FALSE | 0.74 | FALSE | -0.87 | FALSE | -0.5 | FALSE | | -0.59 | |  |
|  | Sordariales Order | FALSE | 0.35 | FALSE | 0.24 | FALSE | 1.24 | FALSE | -0.49 | FALSE | 1.1 | FALSE | 0.48 | FALSE | -0.66 | FALSE | 0.52 | FALSE | 2.09 | FALSE | | 1 | |  |
|  | Sordariomycetes Calss | FALSE | -0.41 | FALSE | -0.04 | FALSE | -0.14 | TRUE | -1.22 | FALSE | -0.31 | FALSE | 0.14 | TRUE | -1.36 | FALSE | -0.16 | FALSE | 0.31 | FALSE | | 0.86 | |  |
|  | Pleosporales Order | TRUE | 1.58 | FALSE | 0.59 | FALSE | -0.82 | FALSE | 0.87 | FALSE | 0.19 | FALSE | -0.22 | FALSE | 0.93 | FALSE | -0.13 | FALSE | -0.08 | FALSE | | 0.31 | |  |
|  | Sporormiaceae | FALSE | 0.15 | FALSE | 0.01 | FALSE | 0.25 | FALSE | -0.28 | FALSE | -0.13 | FALSE | -0.52 | FALSE | -0.43 | FALSE | 0.08 | FALSE | -0.48 | FALSE | | 0.3 | |  |
|  | **Dothideomycetes Class** | FALSE | -0.31 | FALSE | -0.01 | FALSE | -0.54 | TRUE | -0.49 | TRUE | -1.31 | TRUE | -0.84 | TRUE | -0.49 | FALSE | -0.85 | TRUE | -1.59 | FALSE | | -0.2 | |  |
|  | **Saccotheciaceae** | TRUE | 3.53 | FALSE | -0.88 | TRUE | -3.11 | FALSE | 1.5 | FALSE | -0.99 | FALSE | 0.57 | FALSE | 1.13 | FALSE | -0.13 | FALSE | 0.85 | FALSE | | 1.25 | |  |
|  | **Nigrogranaceae** | FALSE | 0.18 | FALSE | 0.4 | FALSE | -0.67 | TRUE | -1.63 | FALSE | 1.01 | FALSE | 1.67 | TRUE | -1.71 | FALSE | 0.82 | FALSE | 1.22 | TRUE | | 2.68 | |  |
|  | **Arthrodermataceae** | FALSE | 0.1 | FALSE | -0.19 | TRUE | 1.45 | FALSE | 0.01 | FALSE | -0.8 | FALSE | -0.28 | FALSE | -0.18 | FALSE | -0.33 | FALSE | -0.09 | FALSE | | 0.34 | |  |
|  | Aspergillaceae | TRUE | 5.23 | FALSE | 0.33 | FALSE | 0.14 | TRUE | 4.78 | FALSE | 0.32 | FALSE | -0.52 | TRUE | 4.91 | FALSE | -0.45 | FALSE | -0.14 | FALSE | | 0.11 | |  |
|  | Helotiaceae | FALSE | -0.06 | FALSE | 0.72 | FALSE | -0.31 | FALSE | 0.15 | FALSE | -0.57 | FALSE | -1.33 | FALSE | 0.12 | FALSE | -0.88 | FALSE | -0.88 | FALSE | | -0.54 | |  |
|  | Didymellaceae | TRUE | 1.19 | FALSE | -0.29 | FALSE | -0.23 | FALSE | 0.7 | FALSE | -0.81 | FALSE | -0.56 | FALSE | 0.62 | FALSE | -0.95 | FALSE | -0.25 | FALSE | | 0.29 | |  |
|  | Helotiales Order | FALSE | 0.31 | FALSE | -0.09 | FALSE | 0.06 | FALSE | 0.26 | FALSE | -1.26 | FALSE | -0.9 | FALSE | 0.2 | FALSE | -0.98 | FALSE | -1.24 | FALSE | | -0.07 | |  |
|  | Thelebolaceae | TRUE | 2.82 | FALSE | -0.81 | FALSE | -1.56 | FALSE | 1.19 | FALSE | 0.08 | FALSE | 0.07 | FALSE | 0.86 | FALSE | 0.89 | FALSE | -0.17 | FALSE | | 0.98 | |  |
|  | **Leotiomycetes Class** | TRUE | 5.65 | FALSE | -1.94 | TRUE | -2.5 | TRUE | 2.89 | FALSE | 0.02 | FALSE | 0.91 | TRUE | 2.73 | FALSE | 0.7 | FALSE | 0.18 | FALSE | | 1.6 | |  |
|  | Pseudeurotiaceae | FALSE | 0.43 | FALSE | 0.21 | FALSE | 0.13 | FALSE | -0.4 | FALSE | 0.15 | FALSE | 0.33 | FALSE | -0.36 | FALSE | -0.15 | FALSE | 0.33 | FALSE | | 0.95 | |  |
|  | Cladosporiaceae | TRUE | 3.79 | FALSE | 0.58 | FALSE | 0.38 | TRUE | 4.26 | FALSE | -1.26 | FALSE | -1.16 | TRUE | 4.12 | FALSE | -1.2 | FALSE | -1.22 | FALSE | | -0.02 | |  |
|  | Clavicipitaceae | TRUE | 0.68 | FALSE | -0.31 | FALSE | 0.09 | FALSE | -0.33 | FALSE | 0.12 | FALSE | 0.2 | FALSE | -0.41 | FALSE | 0.07 | FALSE | 0.36 | FALSE | | 0.88 | |  |
|  | Podosporaceae | FALSE | -0.49 | FALSE | -0.19 | FALSE | -0.66 | TRUE | -1.61 | FALSE | 0.44 | FALSE | -0.14 | TRUE | -1.74 | FALSE | 0.43 | FALSE | 0.34 | FALSE | | 0.51 | |  |
|  | Cephalothecaceae | FALSE | 0.21 | FALSE | -0.16 | FALSE | 0.4 | FALSE | -0.21 | FALSE | -0.32 | FALSE | -0.51 | FALSE | -0.51 | FALSE | -0.54 | FALSE | 0.04 | FALSE | | 0.99 | |  |
|  | **Hypocreaceae** | TRUE | 1.84 | FALSE | 0.14 | TRUE | 1.74 | FALSE | 1.19 | FALSE | 0.26 | FALSE | 0.82 | FALSE | 1.15 | FALSE | 0.29 | FALSE | -0.04 | FALSE | | 1.86 | |  |
|  | **Bionectriaceae** | FALSE | 0.89 | FALSE | -0.17 | FALSE | 0.42 | TRUE | 1.26 | FALSE | -1.3 | TRUE | -1.65 | TRUE | 1.49 | TRUE | -1.99 | TRUE | -2.28 | FALSE | | -0.54 | |  |
|  | Nectriaceae | TRUE | 2.59 | FALSE | 0.62 | FALSE | 0.08 | TRUE | 2.15 | FALSE | 0.11 | FALSE | -0.16 | TRUE | 2.2 | FALSE | -0.32 | FALSE | 0.48 | FALSE | | 0.23 | |  |
|  | Neoschizotheciaceae | FALSE | 0.55 | FALSE | -0.23 | FALSE | -1.04 | FALSE | -0.43 | FALSE | -0.16 | FALSE | -0.35 | FALSE | -0.18 | FALSE | -0.29 | FALSE | -0.56 | FALSE | | -0.38 | |  |

| **Table S12:** ANCOM-BC results for differentially abundant (A) bacterial (V4-5) and (B) fungal (ITS2) gut microbiota genera between the two species. Positive Log fold change values indicate higher abundance compared to the reference, negative values indicate a lower abundance. TRUE indicates a significant difference in abundance between the species, FALSE indicates no significant difference. Bold text indicates statistical significance**.** | | | | | |
| --- | --- | --- | --- | --- | --- |
|  | **Bacterial Genus** | **Intercept** | **Log fold change** | ***M.arv* - *M. gla*** | **Log fold change** |
| **A** | Rodentibacter | TRUE | 1.03 | FALSE | 0.63 |
|  | **Rs-E47_termite_group** | TRUE | 1.54 | TRUE | -1.08 |
|  | Alistipes | FALSE | 0.31 | FALSE | -0.09 |
|  | **Rikenella** | TRUE | -2.56 | TRUE | 1.01 |
|  | **Prevotella** | FALSE | -0.31 | TRUE | -2.59 |
|  | **Muribaculaceae** | TRUE | 5.63 | TRUE | -1.13 |
|  | Marinifilaceae Family | FALSE | 0.17 | FALSE | 0.36 |
|  | **Odoribacter** | TRUE | -1.41 | TRUE | 1.79 |
|  | **Burkholderiales Order** | TRUE | -1.8 | TRUE | 1.35 |
|  | Helicobacter | TRUE | -2.22 | FALSE | -0.31 |
|  | **Desulfovibrionaceae Family** | TRUE | -2.83 | TRUE | 1.16 |
|  | **Desulfovibrio** | TRUE | 4.68 | TRUE | 0.72 |
|  | Bilophila | TRUE | -1.96 | FALSE | 0.16 |
|  | **Cyanobacteriia Class** | TRUE | 1.37 | TRUE | -0.99 |
|  | **Coriobacteriales Order** | TRUE | 1.04 | TRUE | -3.48 |
|  | **Enterorhabdus** | TRUE | 3.13 | TRUE | -0.88 |
|  | **Eggerthellaceae Family** | TRUE | 3.92 | TRUE | -0.45 |
|  | Enteroscipio | TRUE | -1.44 | FALSE | -0.46 |
|  | Gordonibacter | FALSE | 0.26 | FALSE | -0.63 |
|  | Treponema | TRUE | 3.14 | FALSE | -0.44 |
|  | **Cutibacterium** | TRUE | -2.08 | TRUE | -0.89 |
|  | RF39 | TRUE | 3.02 | FALSE | 0.11 |
|  | Erysipelotrichaceae Family | FALSE | 0.43 | FALSE | -0.92 |
|  | **Streptococcus** | TRUE | 1.04 | TRUE | -1.17 |
|  | Staphylococcus | TRUE | -1.21 | FALSE | -0.32 |
|  | **Tyzzerella** | TRUE | -2.75 | TRUE | 1.4 |
|  | [Eubacterium]_brachy_group | TRUE | -0.77 | FALSE | 0.03 |
|  | Family_XIII_AD3011_group | TRUE | -1.35 | FALSE | -0.68 |
|  | Peptococcaceae Family | TRUE | -2.62 | FALSE | 0.19 |
|  | **Christensenellaceae Family** | TRUE | 3.97 | TRUE | -1.25 |
|  | **Christensenellaceae** | TRUE | 1.06 | TRUE | -0.83 |
|  | Clostridia_UCG-014 | TRUE | 1.88 | FALSE | 0.48 |
|  | Clostridia_vadinBB60_group | TRUE | 1.78 | FALSE | 0.28 |
|  | Ruminococcaceae Family | TRUE | 0.81 | FALSE | 0.07 |
|  | UBA1819 | FALSE | -0.19 | FALSE | 0.47 |
|  | **Incertae_Sedis** | TRUE | -0.51 | TRUE | -1.15 |
|  | **Ruminococcus** | TRUE | 1.92 | TRUE | -0.58 |
|  | [Eubacterium]_coprostanoligenes_group | TRUE | -1.09 | FALSE | -0.59 |
|  | [Eubacterium]_siraeum_group | TRUE | 1.06 | FALSE | -0.2 |
|  | Ruminococcaceae | TRUE | 1.22 | FALSE | -0.68 |
|  | **UCG-010** | TRUE | -1.31 | TRUE | -1.29 |
|  | NK4A214_group | TRUE | 0.54 | FALSE | 0.09 |
|  | Oscillospiraceae Family | TRUE | 3.58 | FALSE | 0 |
|  | Intestinimonas | TRUE | -1.21 | FALSE | 0.77 |
|  | Butyricicoccus | TRUE | -1.58 | FALSE | -0.42 |
|  | UCG-005 | TRUE | -1.17 | FALSE | -0.71 |
|  | **Monoglobus** | TRUE | -0.49 | TRUE | -0.96 |
|  | Colidextribacter | TRUE | 1.52 | FALSE | -0.32 |
|  | Oscillibacter | FALSE | 0.24 | FALSE | 0.11 |
|  | **Coriobacteriales_Incertae_Sedis Family** | TRUE | 1.35 | TRUE | -2.83 |
|  | **Parvibacter** | TRUE | 0.73 | TRUE | -3.1 |
|  | Adlercreutzia | TRUE | -0.78 | FALSE | -0.43 |
|  | **Lactobacillus** | TRUE | 3.43 | TRUE | 1.53 |
|  | Bartonella | FALSE | -0.1 | FALSE | -0.22 |
|  | Lachnospiraceae Family | TRUE | 3.89 | FALSE | -0.01 |
|  | Lachnospiraceae_UCG-006 | FALSE | -0.03 | FALSE | 0.31 |
|  | **Allobaculum** | TRUE | 3.79 | TRUE | -1.58 |
|  | Lachnospiraceae_NK4A136_group | TRUE | 3.42 | FALSE | -0.01 |
|  | Roseburia | TRUE | 1.34 | FALSE | -0.3 |
|  | **Lachnospiraceae_UCG-001** | FALSE | -0.54 | TRUE | 1.3 |
|  | [Eubacterium]_xylanophilum_group | TRUE | 1.51 | FALSE | -0.22 |
|  | [Eubacterium]_ruminantium_group | TRUE | -1.48 | FALSE | -0.48 |
|  | **Lachnoclostridium** | FALSE | -0.41 | TRUE | 1.14 |
|  |  |  |  |  |  |
| **B** | **Fungal Genus** | **Intercept** | **Log fold change** | ***M.arv* - *M. gla*** | **Log fold change** |
|  | **Agaricomycetes Class** | TRUE | -1.57 | TRUE | 0.79 |
|  | Laetiporus | TRUE | 1.92 | FALSE | -0.33 |
|  | Hyphodontia | TRUE | -1.17 | FALSE | 0.8 |
|  | Mycena | FALSE | -0.28 | FALSE | -0.21 |
|  | Solicoccozyma | FALSE | 0.11 | FALSE | -0.78 |
|  | **Ganoderma** | TRUE | -0.61 | TRUE | 0.9 |
|  | Heterobasidion | FALSE | -0.65 | FALSE | 0.32 |
|  | Lycoperdon | FALSE | -0.16 | FALSE | 0.85 |
|  | **Clitocybe** | TRUE | -1 | TRUE | 1.18 |
|  | **Agaricales Order** | TRUE | -1.92 | TRUE | 1.15 |
|  | Coprinellus | TRUE | 0.95 | FALSE | 0.46 |
|  | Hypholoma | FALSE | 0.49 | FALSE | 0.15 |
|  | **Coprinopsis** | FALSE | -0.58 | TRUE | -0.84 |
|  | Rigidoporus | TRUE | -1.01 | FALSE | 0.32 |
|  | **Acremonium** | TRUE | 1.43 | TRUE | -1.95 |
|  | Cordyceps | FALSE | 0.44 | FALSE | 0.31 |
|  | **Puccinia** | FALSE | 0.82 | TRUE | -2.3 |
|  | Sporobolomyces | FALSE | -0.47 | FALSE | -0.67 |
|  | **Ustilago** | TRUE | 1.09 | TRUE | -2.6 |
|  | **Protomyces** | TRUE | 1.96 | TRUE | -3.59 |
|  | **Vishniacozyma** | TRUE | 2.64 | TRUE | -1.09 |
|  | **Pilaira** | TRUE | 5.21 | TRUE | -5.08 |
|  | **Mucor** | TRUE | 4.97 | TRUE | 1.72 |
|  | Mucoraceae Family | TRUE | -0.69 | FALSE | 0.09 |
|  | **Mucoraceae_gen_Incertae_sedis** | FALSE | 0.05 | TRUE | 1.63 |
|  | Ascomycota Phylum | TRUE | 0.73 | FALSE | 0.21 |
|  | Malassezia | TRUE | 2.1 | FALSE | -0.93 |
|  | **Trechispora** | TRUE | -0.98 | TRUE | 2.26 |
|  | **Buckleyzyma** | TRUE | -1.63 | TRUE | 1.85 |
|  | **Cystobasidiomycetes_gen_Incertae_sedis** | TRUE | 1.04 | TRUE | -1.84 |
|  | Symmetrospora | FALSE | 0.12 | FALSE | -0.63 |
|  | Candida | TRUE | 2.03 | FALSE | -0.11 |
|  | **Mortierella** | TRUE | -1.02 | TRUE | 2.42 |
|  | Apiotrichum | TRUE | -1 | FALSE | 0.82 |
|  | **Chaetothyriales Order** | TRUE | -2.02 | TRUE | 2.2 |
|  | **Exophiala** | FALSE | 0.2 | TRUE | -1.32 |
|  | **Cyphellophora** | FALSE | 0.09 | TRUE | -1.04 |
|  | Sordariales Order | FALSE | 0.68 | FALSE | 0.02 |
|  | Sordariomycetes Class | FALSE | -0.15 | FALSE | -0.43 |
|  | **Cyphellophoraceae_gen_Incertae_sedis** | TRUE | 0.7 | TRUE | -2.56 |
|  | **Pseudeurotiaceae Family** | TRUE | 0.88 | TRUE | 2.19 |
|  | **Leotiomycetes Class** | TRUE | 0.92 | TRUE | 3.04 |
|  | **Pleosporales Order** | TRUE | 4.73 | TRUE | -3.24 |
|  | **Preussia** | TRUE | 3.06 | TRUE | -3.15 |
|  | **Sporormiella** | FALSE | 0.3 | TRUE | -1.74 |
|  | Dothideomycetes Class | FALSE | -0.42 | FALSE | -0.16 |
|  | **Aureobasidium** | FALSE | 0.31 | TRUE | 1.82 |
|  | **Periconia** | TRUE | 2.79 | TRUE | -2.54 |
|  | **Pseudopithomyces** | TRUE | 1.92 | TRUE | -2.3 |
|  | **Didymosphaeriaceae Family** | FALSE | 0.32 | TRUE | -1.41 |
|  | Keissleriella | TRUE | 1.02 | FALSE | 0 |
|  | **Aspergillus** | TRUE | 3.21 | TRUE | -0.9 |
|  | **Pyrenochaetopsis** | TRUE | 2.66 | TRUE | -3.05 |
|  | **Pleosporales_gen_Incertae_sedis** | TRUE | 1.41 | TRUE | -2.74 |
|  | Pleosporaceae Family | FALSE | 0.22 | FALSE | -1.03 |
|  | **Paraphaeosphaeria** | TRUE | 3.26 | TRUE | -4.26 |
|  | Botrytis | FALSE | -0.36 | FALSE | -0.1 |
|  | **Phaeosphaeriaceae Family** | TRUE | 0.72 | TRUE | -2.34 |
|  | Alternaria | TRUE | -0.57 | FALSE | -0.18 |
|  | Boeremia | TRUE | 2.4 | FALSE | -0.16 |
|  | **Didymellaceae Family** | TRUE | 1.45 | TRUE | -0.88 |
|  | **Neosetophoma** | TRUE | 1.65 | TRUE | -1.59 |
|  | Stagonospora | FALSE | 0.16 | FALSE | -0.63 |
|  | **Filobasidium** | FALSE | -0.04 | TRUE | -1.03 |
|  | **Helotiales Order** | TRUE | -1.01 | TRUE | 1.18 |
|  | **Thelebolus** | TRUE | 5.07 | TRUE | -3.42 |
|  | **Cladosporium** | TRUE | 5.08 | TRUE | -1.04 |
|  | Penicillium | TRUE | 4.38 | FALSE | 0.63 |
|  | Aspergillaceae Family | FALSE | -0.18 | FALSE | 0 |
|  | **Oidiodendron** | TRUE | -1.36 | TRUE | 2.9 |
|  | **Taphrina** | TRUE | -0.96 | TRUE | 2.59 |
|  | **Triangularia** | FALSE | 0.94 | TRUE | -2.26 |
|  | **Podospora** | TRUE | 1.82 | TRUE | -3.15 |
|  | **Hypocreales Order** | TRUE | -1.89 | TRUE | 2.29 |
|  | **Keithomyces** | TRUE | -0.69 | TRUE | 1.03 |
|  | Gibellulopsis | FALSE | 0.08 | FALSE | -0.71 |
|  | **Fusarium** | TRUE | 3.14 | TRUE | -1.31 |
|  | **Microascus** | TRUE | 1.94 | TRUE | -2.42 |
|  | **Cephalotrichum** | TRUE | 0.59 | TRUE | 1.17 |
|  | **Gamsia** | TRUE | 0.79 | TRUE | -1.11 |
|  | **Trichoderma** | FALSE | -0.64 | TRUE | 2.93 |
|  | Clonostachys | FALSE | -0.4 | FALSE | -0.15 |
|  | Nectriaceae Family | TRUE | -1.03 | FALSE | 0.12 |
|  | Myrmecridium | FALSE | -0.26 | FALSE | -0.78 |
|  | **Neoschizothecium** | TRUE | 3.85 | TRUE | -3.82 |
|  | **Metapochonia** | TRUE | -1.72 | TRUE | 1.83 |
|  | **Cordycipitaceae Family** | TRUE | -0.82 | TRUE | 0.84 |
|  | **Sarocladium** | TRUE | 1.21 | TRUE | -1.93 |
|  | **Myxospora** | FALSE | 0.52 | TRUE | -2.16 |
|  | Gibberella | FALSE | 0.55 | FALSE | -0.9 |

|  | **Table S13:** ANCOM-BC results for differentially abundant (A) bacterial (V4-5) and (B) fungal (ITS2) gut microbiota in *Microtus arvalis* between land-use intensities and sesons. Positive Log fold change values indicate higher abundance compared to the reference, negative values indicate a lower abundance. TRUE indicates a significant difference in abundance between the species, FALSE indicates no significant difference. Bold text indicates statistical significance. | | | | | | | | | | | | | | | | | | |
| --- | --- | --- | --- | --- | --- | --- | --- | --- | --- | --- | --- | --- | --- | --- | --- | --- | --- | --- | --- |
|  | Bacterial Genus | **Season** | | | | | | **Land-use intensity** | | | | | | **Land-use type** | | | | | |
| **A** |  | **Inter-cept** | **LFC** | **Jun - Nov** | **LFC** | **Jun - Sep** | **LFC** | **Inter-cept** | **LFC** | **high - low** | **LFC** | **high - medium** | **LFC** | **Inter-cept** | **LFC** | **MP - P** | **LFC** | **MP - M** | **LFC** |
|  | Rodentibacter | FALSE | -0.08 | FALSE | 1.47 | TRUE | 2.51 | TRUE | 2.08 | FALSE | -0.76 | FALSE | 0.69 | TRUE | 1.1 | FALSE | -0.22 | FALSE | 1.09 |
|  | Gammaproteobacteria Class | TRUE | -2.43 | FALSE | 0.96 | FALSE | 1.39 | FALSE | -0.89 | FALSE | -0.24 | FALSE | 0.16 | TRUE | -1.63 | FALSE | -0.33 | FALSE | 0.19 |
|  | Rs-E47_termite_group | TRUE | 1.8 | FALSE | -0.3 | FALSE | 0.35 | TRUE | 2.67 | FALSE | -0.23 | FALSE | -0.32 | TRUE | 1.82 | FALSE | -0.29 | FALSE | 0.01 |
|  | Alistipes | FALSE | 0.78 | FALSE | -0.46 | FALSE | -0.06 | TRUE | 0.95 | FALSE | 0.37 | FALSE | 0.66 | FALSE | 0.35 | FALSE | -0.04 | FALSE | 0.76 |
|  | Prevotella | FALSE | -0.48 | FALSE | 0.65 | FALSE | 0.67 | FALSE | 0.61 | FALSE | 0.3 | FALSE | -0.27 | FALSE | 0.2 | FALSE | -0.39 | FALSE | -1.01 |
|  | Prevotellaceae_UCG-001 | TRUE | -1.81 | FALSE | 1.68 | FALSE | 0.88 | FALSE | 0.3 | FALSE | -1.43 | FALSE | -0.11 | FALSE | -0.33 | FALSE | -1.62 | FALSE | -0.09 |
|  | Prevotellaceae_UCG-003 | TRUE | -2.21 | FALSE | 0.88 | FALSE | 0.33 | TRUE | -0.97 | FALSE | -0.27 | FALSE | -0.21 | TRUE | -1.73 | FALSE | -0.23 | FALSE | -0.56 |
|  | **Muribaculaceae** | TRUE | 5.78 | FALSE | 0.04 | FALSE | 0.37 | TRUE | 6.81 | FALSE | -0.57 | FALSE | -0.02 | TRUE | 6.08 | TRUE | -0.69 | FALSE | 0.1 |
|  | Marinifilaceae Family | FALSE | 0.68 | FALSE | -0.6 | FALSE | -0.07 | TRUE | 0.97 | FALSE | 0.14 | FALSE | 0.38 | FALSE | 0.34 | FALSE | -0.05 | FALSE | 0.04 |
|  | Odoribacter | TRUE | -1.27 | FALSE | 0.35 | FALSE | 0.09 | FALSE | -0.24 | FALSE | -0.65 | FALSE | 0.1 | TRUE | -1.18 | FALSE | -0.42 | FALSE | 0.61 |
|  | Burkholderiales Order | TRUE | -1.19 | FALSE | -0.39 | FALSE | -0.53 | TRUE | -0.99 | FALSE | 0.62 | FALSE | -0.29 | TRUE | -1.92 | FALSE | 0.57 | FALSE | 0.1 |
|  | Helicobacter | TRUE | -1.6 | FALSE | -0.69 | FALSE | -0.3 | FALSE | -0.41 | FALSE | -1.21 | FALSE | -1.46 | TRUE | -1.83 | FALSE | -0.27 | FALSE | -0.62 |
|  | Desulfovibrio | TRUE | 5.05 | FALSE | -0.38 | FALSE | 0.13 | TRUE | 5.68 | FALSE | -0.04 | FALSE | -0.1 | TRUE | 4.94 | FALSE | -0.14 | FALSE | -0.23 |
|  | Bilophila | TRUE | -1.81 | FALSE | -0.03 | FALSE | 0.41 | TRUE | -1.01 | FALSE | -0.11 | FALSE | 0.21 | TRUE | -1.9 | FALSE | 0.03 | FALSE | 0.49 |
|  | Cyanobacteriia Class | TRUE | 1.34 | FALSE | -0.05 | FALSE | 0.92 | TRUE | 2.03 | FALSE | 0.67 | FALSE | 0.2 | TRUE | 1.06 | FALSE | 0.98 | FALSE | 0.2 |
|  | **Gastranaerophilales** | TRUE | -2.8 | TRUE | 2.57 | TRUE | 2.68 | FALSE | -0.8 | FALSE | 0.56 | FALSE | 1.03 | TRUE | -1.31 | FALSE | -0.03 | FALSE | 1.23 |
|  | Coriobacteriales Order | TRUE | 1.16 | FALSE | 0.25 | FALSE | 0.24 | TRUE | 1.88 | FALSE | 0.17 | FALSE | 0.2 | TRUE | 1.18 | FALSE | -0.06 | FALSE | 0.25 |
|  | Enterorhabdus | TRUE | 3.6 | FALSE | -0.31 | FALSE | -0.22 | TRUE | 4.18 | FALSE | -0.07 | FALSE | -0.25 | TRUE | 3.44 | FALSE | -0.26 | FALSE | -0.22 |
|  | Eggerthellaceae Family | TRUE | 4.21 | FALSE | -0.06 | FALSE | 0.04 | TRUE | 4.85 | FALSE | 0.14 | FALSE | -0.1 | TRUE | 4.05 | FALSE | 0.08 | FALSE | -0.04 |
|  | Enteroscipio | TRUE | -1.37 | FALSE | -0.05 | FALSE | 0.63 | FALSE | -0.49 | FALSE | 0.55 | FALSE | -0.72 | TRUE | -1.44 | FALSE | 0.62 | FALSE | -0.67 |
|  | Gordonibacter | FALSE | 0.16 | FALSE | 0.25 | FALSE | 0.85 | TRUE | 1.23 | FALSE | 0.17 | FALSE | -0.22 | FALSE | 0.25 | FALSE | 0.3 | FALSE | 0.25 |
|  | Treponema | TRUE | 3.8 | FALSE | -0.77 | FALSE | -0.32 | TRUE | 4.18 | FALSE | -0.21 | FALSE | -0.01 | TRUE | 3.63 | FALSE | -0.62 | FALSE | -0.35 |
|  | Cutibacterium | TRUE | -1.91 | FALSE | -0.16 | FALSE | 0.46 | TRUE | -1.04 | FALSE | -0.28 | FALSE | 0.08 | TRUE | -2.17 | FALSE | 0.15 | FALSE | 1.05 |
|  | RF39 | TRUE | 3.27 | FALSE | -0.08 | FALSE | 0.18 | TRUE | 3.98 | FALSE | -0.06 | FALSE | 0.1 | TRUE | 3.07 | FALSE | 0.01 | FALSE | 0.59 |
|  | **Erysipelotrichaceae Family** | FALSE | -0.31 | TRUE | 2.51 | FALSE | 0.56 | TRUE | 2.07 | FALSE | -1.15 | FALSE | -0.94 | TRUE | 1.32 | FALSE | -1.34 | FALSE | -0.79 |
|  | Streptococcus | TRUE | 0.92 | FALSE | 0.66 | FALSE | 0.53 | TRUE | 2.35 | FALSE | -0.56 | FALSE | -0.53 | TRUE | 1.23 | FALSE | -0.03 | FALSE | -0.11 |
|  | Staphylococcus | FALSE | -1.04 | FALSE | -0.52 | FALSE | 0.81 | FALSE | 0.09 | FALSE | -0.47 | FALSE | -0.58 | TRUE | -0.91 | FALSE | -0.38 | FALSE | 0.15 |
|  | **[Eubacterium]_brachy_group** | FALSE | -0.78 | TRUE | 1.62 | FALSE | -0.62 | FALSE | 0.08 | FALSE | -0.35 | FALSE | 0.85 | FALSE | -0.57 | FALSE | -0.57 | FALSE | 1.22 |
|  | Family_XIII_AD3011_group | TRUE | -1.05 | FALSE | 0 | FALSE | -0.04 | TRUE | -0.9 | FALSE | 0.82 | FALSE | 0.73 | TRUE | -1.33 | FALSE | 0.08 | FALSE | 0.56 |
|  | Peptococcaceae Family | TRUE | -1.9 | FALSE | -0.56 | FALSE | -0.64 | TRUE | -1.66 | FALSE | 0.08 | FALSE | -0.08 | TRUE | -2.59 | FALSE | 0.33 | FALSE | -0.1 |
|  | **Christensenellaceae Family** | TRUE | 3.3 | TRUE | 1.85 | FALSE | 0.98 | TRUE | 5.57 | FALSE | -1.12 | FALSE | -0.82 | TRUE | 4.46 | FALSE | -0.61 | FALSE | -0.34 |
|  | Christensenellaceae | TRUE | 1.22 | FALSE | 0.46 | FALSE | -0.04 | TRUE | 2.08 | FALSE | -0.14 | FALSE | -0.03 | TRUE | 1.46 | FALSE | -0.46 | FALSE | -0.21 |
|  | Christensenella | TRUE | -2.04 | FALSE | -0.09 | FALSE | 0.3 | TRUE | -1.2 | FALSE | -0.37 | FALSE | 0.17 | TRUE | -1.63 | FALSE | -0.87 | FALSE | -0.45 |
|  | Clostridia_UCG-014 | TRUE | 2.01 | FALSE | 0.23 | FALSE | 0.23 | TRUE | 2.72 | FALSE | 0.18 | FALSE | 0.19 | TRUE | 1.97 | FALSE | -0.02 | FALSE | 0.42 |
|  | Clostridia_vadinBB60_group | TRUE | 1.9 | FALSE | 0.43 | FALSE | 0.08 | TRUE | 2.82 | FALSE | -0.22 | FALSE | 0.03 | TRUE | 2.04 | FALSE | -0.24 | FALSE | 0.04 |
|  | Ruminococcaceae Family | TRUE | 0.97 | FALSE | 0.5 | FALSE | -0.11 | TRUE | 1.42 | FALSE | 0.31 | FALSE | 0.85 | TRUE | 1.06 | FALSE | -0.33 | FALSE | 0.29 |
|  | UBA1819 | FALSE | 0.71 | FALSE | -0.97 | FALSE | -0.81 | FALSE | 0.31 | FALSE | 0.92 | FALSE | 0.43 | FALSE | -0.26 | FALSE | 0.48 | FALSE | 0.06 |
|  | Incertae_Sedis | FALSE | -0.11 | FALSE | 0.17 | FALSE | -0.44 | TRUE | 0.71 | FALSE | -0.51 | FALSE | -0.29 | FALSE | -0.1 | FALSE | -0.35 | FALSE | -0.54 |
|  | **Ruminococcus** | TRUE | 1.57 | TRUE | 1.08 | TRUE | 0.78 | TRUE | 2.96 | FALSE | -0.16 | FALSE | -0.06 | TRUE | 1.93 | FALSE | 0.17 | FALSE | 0.41 |
|  | [Eubacterium]_coprostanoligenes_group | FALSE | -1.06 | FALSE | 0.66 | FALSE | 0.12 | FALSE | -0.15 | FALSE | -0.13 | FALSE | 0.25 | TRUE | -1.05 | FALSE | -0.11 | FALSE | 0.97 |
|  | [Eubacterium]_siraeum_group | FALSE | 0.91 | FALSE | 0.35 | FALSE | 0.9 | TRUE | 1.96 | FALSE | -0.09 | FALSE | 0.33 | TRUE | 1.3 | FALSE | -0.3 | FALSE | 0.3 |
|  | Ruminococcaceae | TRUE | 1.36 | FALSE | -0.15 | FALSE | 0.54 | TRUE | 2.38 | FALSE | -0.42 | FALSE | -0.17 | TRUE | 1.81 | FALSE | -0.71 | FALSE | -0.65 |
|  | UCG-010 | TRUE | -1.73 | FALSE | 1.21 | FALSE | 0.85 | FALSE | -0.09 | FALSE | -0.72 | FALSE | -0.01 | FALSE | -0.98 | FALSE | -0.68 | FALSE | 0.74 |
|  | NK4A214_group | FALSE | 0.55 | FALSE | 0.5 | FALSE | 0.3 | TRUE | 1.25 | FALSE | 0.45 | FALSE | 0.29 | FALSE | 0.56 | FALSE | 0.22 | FALSE | 0.21 |
|  | Oscillospiraceae Family | TRUE | 3.95 | FALSE | -0.01 | FALSE | -0.19 | TRUE | 4.46 | FALSE | 0.14 | FALSE | 0.13 | TRUE | 3.71 | FALSE | 0.05 | FALSE | 0.06 |
|  | Intestinimonas | FALSE | -0.67 | FALSE | -0.23 | FALSE | -0.49 | FALSE | -0.65 | FALSE | 0.87 | FALSE | 0.28 | TRUE | -1.2 | FALSE | 0.41 | FALSE | -0.2 |
|  | **Butyricicoccus** | FALSE | -0.65 | TRUE | -2.06 | FALSE | 0.1 | FALSE | -0.6 | FALSE | 0.44 | FALSE | -0.64 | TRUE | -1.84 | FALSE | 1 | FALSE | -0.15 |
|  | UCG-005 | TRUE | -1.13 | FALSE | 0.99 | FALSE | -0.2 | FALSE | 0.02 | FALSE | -0.58 | FALSE | -0.06 | FALSE | -0.49 | FALSE | -0.92 | FALSE | -0.64 |
|  | Monoglobus | FALSE | -0.53 | FALSE | 0.43 | FALSE | 0.52 | FALSE | 0.17 | FALSE | 0.37 | FALSE | 0.57 | FALSE | -0.46 | FALSE | 0.1 | FALSE | 0.46 |
|  | Colidextribacter | TRUE | 1.91 | FALSE | -0.29 | FALSE | 0 | TRUE | 2.58 | FALSE | -0.03 | FALSE | -0.32 | TRUE | 1.69 | FALSE | 0.06 | FALSE | -0.21 |
|  | Oscillibacter | FALSE | 0.63 | FALSE | 0.16 | FALSE | -0.4 | TRUE | 1 | FALSE | 0.5 | FALSE | 0.07 | FALSE | 0.43 | FALSE | 0.06 | FALSE | -0.31 |
|  | **Coriobacteriales_Incertae_Sedis Family** | TRUE | 1.14 | FALSE | 0.65 | TRUE | 0.79 | TRUE | 2.39 | FALSE | -0.09 | FALSE | -0.14 | TRUE | 1.7 | FALSE | -0.34 | FALSE | -0.2 |
|  | Parvibacter | TRUE | 1.15 | FALSE | -0.21 | FALSE | -0.16 | TRUE | 1.82 | FALSE | -0.04 | FALSE | -0.4 | TRUE | 0.94 | FALSE | -0.07 | FALSE | -0.09 |
|  | Adlercreutzia | FALSE | -0.74 | FALSE | 0.33 | FALSE | 0.37 | FALSE | 0.03 | FALSE | 0.32 | FALSE | 0.1 | FALSE | -0.68 | FALSE | 0.08 | FALSE | 0.11 |
|  | Lactobacillus | TRUE | 3.43 | FALSE | 0.78 | FALSE | 0.1 | TRUE | 4.79 | FALSE | -1.1 | FALSE | 0.01 | TRUE | 3.8 | FALSE | -0.79 | FALSE | 0.73 |
|  | Enterococcus | FALSE | -1.37 | FALSE | -0.44 | FALSE | 0.9 | FALSE | -0.33 | FALSE | -0.41 | FALSE | -0.13 | TRUE | -1.58 | FALSE | 0.03 | FALSE | 1.42 |
|  | **Bartonella** | TRUE | 1.36 | TRUE | -2.95 | FALSE | -0.54 | FALSE | 1.21 | FALSE | -0.35 | FALSE | -0.79 | FALSE | 0.27 | FALSE | -0.12 | FALSE | -0.9 |
|  | Lachnospiraceae Family | TRUE | 4.15 | FALSE | 0.19 | FALSE | -0.09 | TRUE | 4.76 | FALSE | 0.04 | FALSE | 0.26 | TRUE | 4.16 | FALSE | -0.29 | FALSE | 0.07 |
|  | Lachnospiraceae_UCG-006 | TRUE | 1.06 | FALSE | -0.92 | FALSE | -1.35 | FALSE | 0.66 | FALSE | 0.11 | FALSE | 0.82 | FALSE | 0.37 | FALSE | -0.72 | FALSE | 0.47 |
|  | **Allobaculum** | TRUE | 2.46 | TRUE | 3.22 | FALSE | 1.53 | TRUE | 5.19 | FALSE | -1.15 | FALSE | -0.07 | TRUE | 4.41 | FALSE | -1.02 | FALSE | -0.08 |
|  | Lachnospiraceae_NK4A136_group | TRUE | 3.59 | FALSE | 0.43 | FALSE | -0.07 | TRUE | 4.44 | FALSE | -0.23 | FALSE | 0.09 | TRUE | 3.77 | FALSE | -0.37 | FALSE | -0.13 |
|  | Roseburia | TRUE | 1.57 | FALSE | 0.09 | FALSE | 0.11 | TRUE | 2.03 | FALSE | 0.34 | FALSE | 0.53 | TRUE | 1.44 | FALSE | 0.02 | FALSE | 0.29 |
|  | **Lachnospiraceae_UCG-001** | TRUE | 0.92 | TRUE | -2.55 | FALSE | -0.91 | FALSE | 0.27 | FALSE | 1 | FALSE | -0.77 | FALSE | -0.77 | FALSE | 0.99 | FALSE | -0.29 |
|  | **Lachnospiraceae_NK4B4_group** | TRUE | -2.19 | TRUE | 2.51 | FALSE | 1.38 | FALSE | -0.15 | FALSE | -0.08 | FALSE | -0.04 | TRUE | -1.36 | FALSE | 0.36 | FALSE | 1.18 |
|  | [Eubacterium]_xylanophilum_group | TRUE | 1.48 | FALSE | 0.77 | FALSE | 0.18 | TRUE | 2.78 | FALSE | -0.53 | FALSE | -0.43 | TRUE | 1.88 | FALSE | -0.45 | FALSE | -0.08 |
|  | **[Eubacterium]_ruminantium_group** | TRUE | -1.37 | FALSE | 0.86 | FALSE | -0.28 | FALSE | 0.37 | FALSE | -0.9 | TRUE | -2.04 | FALSE | -0.91 | FALSE | -0.45 | FALSE | -1.22 |
|  | Lachnoclostridium | FALSE | 0.27 | FALSE | -0.49 | FALSE | -0.61 | FALSE | 0.85 | FALSE | -0.76 | FALSE | -0.07 | FALSE | 0.27 | FALSE | -1.02 | FALSE | -0.36 |

|  | **Table S14:** ANCOM-BC results for differentially abundant (A) bacterial (V4-5) and (B) fungal (ITS2) gut microbiota in *Myodes glareolus* between land-use intensities and sesons. Positive Log fold change values indicate higher abundance compared to the reference, negative values indicate a lower abundance. TRUE indicates a significant difference in abundance between the species, FALSE indicates no significant difference. Bold text indicates statistical significance. | | | | | | | | | | | | | | | | | | | | |
| --- | --- | --- | --- | --- | --- | --- | --- | --- | --- | --- | --- | --- | --- | --- | --- | --- | --- | --- | --- | --- | --- |
|  | Bacterial Genus | **Season** | | | | | | **Land-use intensity** | | | | | | **Land-use type** | | | | | | | |
| **A** |  | **Inter-cept** | **LFC** | **Jun - Nov** | **LFC** | **Jun - Sep** | **LFC** | **Inter-cept** | **LFC** | **high - low** | **LFC** | **high - medium** | **LFC** | **Inter-cept** | **LFC** | **C - Old-MB** | **LFC** | **C - Un-MB** | **LFC** | **C - Young-MB** |  |
|  | Rodentibacter | FALSE | 1.03 | FALSE | 0.96 | FALSE | 0.97 | FALSE | 0.37 | FALSE | 0.7 | FALSE | 0.61 | FALSE | 0.49 | FALSE | 1.11 | FALSE | -0.48 | FALSE | 1.04 |
|  | Rs-E47_termite_group | FALSE | 0.55 | FALSE | -0.61 | FALSE | 0.46 | FALSE | -0.38 | FALSE | -0.57 | FALSE | 0.48 | FALSE | 0.01 | FALSE | -0.15 | FALSE | -1.29 | FALSE | 0.37 |
|  | Rikenella | TRUE | -1.37 | FALSE | -0.38 | FALSE | -0.15 | TRUE | -1.66 | FALSE | -0.97 | FALSE | -0.95 | TRUE | -1.46 | FALSE | -0.78 | FALSE | -1.52 | FALSE | -0.73 |
|  | **Alistipes** | TRUE | 0.97 | TRUE | -1.43 | FALSE | -0.73 | FALSE | -0.37 | FALSE | -0.53 | FALSE | -0.15 | FALSE | 0.06 | FALSE | -0.82 | FALSE | -0.69 | FALSE | -0.33 |
|  | Muribaculaceae | TRUE | 4.75 | FALSE | -0.91 | FALSE | 0.31 | TRUE | 3.64 | FALSE | -0.25 | FALSE | 0.3 | TRUE | 3.82 | FALSE | -0.23 | FALSE | -0.11 | FALSE | 0.47 |
|  | Marinifilaceae Family | FALSE | 0.43 | FALSE | -0.08 | FALSE | 0.45 | FALSE | -0.04 | FALSE | -0.26 | FALSE | -0.4 | FALSE | 0.21 | FALSE | -0.41 | FALSE | -0.43 | FALSE | -0.43 |
|  | Odoribacter | FALSE | 0.06 | FALSE | 0.51 | FALSE | 0.45 | FALSE | -0.57 | FALSE | 0.24 | FALSE | 0.14 | FALSE | -0.47 | FALSE | 0.6 | FALSE | -0.2 | FALSE | 0.2 |
|  | Oxalobacter | TRUE | -2.42 | FALSE | 0.79 | FALSE | 0 | TRUE | -3.22 | FALSE | 0.83 | FALSE | 0.05 | TRUE | -2.84 | FALSE | 0.29 | FALSE | 0.18 | FALSE | -0.21 |
|  | **Burkholderiales Order** | FALSE | -1.4 | FALSE | 1.25 | TRUE | 1.69 | FALSE | -1.38 | FALSE | 0.58 | FALSE | -0.17 | FALSE | -1.18 | FALSE | 0.31 | FALSE | -0.19 | FALSE | 0.09 |
|  | Helicobacter | TRUE | -2.69 | FALSE | 0.15 | FALSE | 0.34 | TRUE | -4.04 | FALSE | 0.87 | FALSE | 0.99 | TRUE | -3.71 | FALSE | 1.04 | FALSE | 0.65 | FALSE | 0.29 |
|  | **Desulfovibrionaceae Family** | TRUE | -2.82 | TRUE | 2.29 | FALSE | 1.07 | TRUE | -3.15 | FALSE | 0.55 | FALSE | 1.2 | TRUE | -2.98 | FALSE | 0.82 | FALSE | 0.75 | FALSE | 0.99 |
|  | Desulfovibrio | TRUE | 5.96 | FALSE | -0.74 | FALSE | -1.04 | TRUE | 4.68 | FALSE | -0.31 | FALSE | 0 | TRUE | 4.9 | FALSE | -0.36 | FALSE | 0.17 | FALSE | -0.26 |
|  | Bilophila | TRUE | -2.31 | FALSE | 0.87 | FALSE | 0.67 | TRUE | -3.09 | FALSE | 1.31 | FALSE | 0.14 | TRUE | -2.72 | FALSE | 1.13 | FALSE | -0.18 | FALSE | -0.15 |
|  | Cyanobacteriia Class | FALSE | 0.83 | FALSE | -0.58 | FALSE | -0.86 | FALSE | 0.02 | FALSE | -0.73 | FALSE | -0.55 | FALSE | 0.23 | FALSE | -0.28 | FALSE | -1.58 | FALSE | -0.4 |
|  | **Enterorhabdus** | TRUE | 2.76 | FALSE | -0.56 | TRUE | -1.1 | TRUE | 1.63 | FALSE | -0.28 | FALSE | -0.27 | TRUE | 1.84 | FALSE | -0.3 | FALSE | -0.4 | FALSE | -0.24 |
|  | **Eggerthellaceae Family** | TRUE | 3.95 | FALSE | -0.44 | TRUE | -1.14 | TRUE | 2.78 | FALSE | 0.01 | FALSE | -0.31 | TRUE | 3.05 | FALSE | -0.11 | FALSE | -0.39 | FALSE | -0.41 |
|  | **Gordonibacter** | TRUE | 0.76 | TRUE | -1.67 | TRUE | -1.76 | FALSE | -0.49 | FALSE | -0.91 | FALSE | -0.97 | FALSE | -0.35 | FALSE | -0.69 | FALSE | -1.32 | FALSE | -0.69 |
|  | **Treponema** | TRUE | 3.69 | TRUE | -1.98 | FALSE | -0.88 | TRUE | 2.3 | FALSE | -1.63 | FALSE | 0.24 | TRUE | 2.58 | FALSE | -1.6 | FALSE | -0.53 | FALSE | 0.32 |
|  | Borreliella | TRUE | -2.65 | FALSE | 0.07 | FALSE | -0.62 | TRUE | -3.94 | FALSE | 0.35 | FALSE | 0.53 | TRUE | -3.71 | FALSE | 0.33 | FALSE | 0.36 | FALSE | 0.37 |
|  | RF39 | TRUE | 3.4 | FALSE | -0.42 | FALSE | -0.38 | TRUE | 2.29 | FALSE | 0.41 | FALSE | -0.24 | TRUE | 2.45 | FALSE | 0.08 | FALSE | 0.17 | FALSE | -0.07 |
|  | Erysipelotrichaceae Family | FALSE | -0.98 | FALSE | 1.23 | FALSE | 0.1 | FALSE | -0.88 | FALSE | 0.08 | FALSE | -1.08 | FALSE | -0.6 | FALSE | -0.91 | FALSE | -0.08 | FALSE | -0.86 |
|  | Streptococcus | FALSE | -0.44 | FALSE | 0.66 | FALSE | 0.22 | FALSE | -0.97 | FALSE | -0.02 | FALSE | 0.08 | FALSE | -0.67 | FALSE | -0.22 | FALSE | -0.25 | FALSE | 0.08 |
|  | Staphylococcus | TRUE | -1.51 | FALSE | -0.37 | FALSE | 0.42 | TRUE | -1.45 | FALSE | -1.28 | FALSE | -1.18 | TRUE | -1.26 | FALSE | -1.66 | FALSE | -1.05 | FALSE | -0.69 |
|  | Tyzzerella | TRUE | -1.84 | FALSE | 1.5 | FALSE | -0.22 | TRUE | -2.92 | FALSE | 0.87 | FALSE | 1.16 | TRUE | -2.48 | FALSE | -0.12 | FALSE | 1.24 | FALSE | 1.08 |
|  | **[Eubacterium]_brachy_group** | TRUE | -1.42 | FALSE | 0.48 | TRUE | 1.78 | TRUE | -1.53 | FALSE | 0.59 | FALSE | -0.49 | TRUE | -1.53 | FALSE | -0.27 | FALSE | 0.35 | FALSE | 0.64 |
|  | Family_XIII_AD3011_group | TRUE | -2.45 | FALSE | 0.61 | FALSE | 0.68 | TRUE | -2.48 | FALSE | -0.08 | FALSE | -0.81 | TRUE | -2.21 | FALSE | -0.51 | FALSE | -0.29 | FALSE | -0.89 |
|  | Peptococcaceae Family | TRUE | -2.52 | FALSE | 0.26 | FALSE | -0.01 | TRUE | -3.43 | FALSE | 0.23 | FALSE | 0.26 | TRUE | -3.39 | FALSE | 0.22 | FALSE | 0.53 | FALSE | 0.53 |
|  | **Christensenellaceae Family** | TRUE | 1.82 | TRUE | 2.22 | FALSE | 0.26 | TRUE | 1.81 | FALSE | -0.01 | FALSE | 0.24 | TRUE | 2.21 | FALSE | -0.77 | FALSE | 0.03 | FALSE | 0.39 |
|  | Christensenellaceae | FALSE | -0.08 | FALSE | 0.38 | FALSE | 0.6 | FALSE | -0.86 | FALSE | 0.12 | FALSE | 0.56 | FALSE | -0.7 | FALSE | 0.07 | FALSE | 0.29 | FALSE | 0.79 |
|  | Clostridia_UCG-014 | TRUE | 2.32 | FALSE | 0.27 | FALSE | -0.22 | TRUE | 1.47 | FALSE | 0.37 | FALSE | -0.11 | TRUE | 1.68 | FALSE | 0.24 | FALSE | 0.16 | FALSE | -0.25 |
|  | Clostridia_vadinBB60_group | TRUE | 2.27 | FALSE | -0.26 | FALSE | -0.4 | TRUE | 1.24 | FALSE | 0.27 | FALSE | -0.19 | TRUE | 1.66 | FALSE | -0.14 | FALSE | -0.29 | FALSE | -0.53 |
|  | Ruminococcaceae Family | FALSE | 0.63 | FALSE | 0.33 | FALSE | 0.44 | FALSE | 0.29 | FALSE | 0.39 | FALSE | -0.83 | TRUE | 0.78 | FALSE | -0.68 | FALSE | -0.33 | FALSE | -1.01 |
|  | UBA1819 | FALSE | 0.35 | FALSE | -0.3 | FALSE | 0.13 | FALSE | -0.99 | FALSE | 0.7 | FALSE | 0.55 | FALSE | -0.41 | FALSE | -0.29 | FALSE | 1 | FALSE | -0.2 |
|  | Incertae_Sedis | TRUE | -1.59 | FALSE | 0.1 | FALSE | -0.38 | TRUE | -2.61 | FALSE | 0.25 | FALSE | 0.13 | TRUE | -2.4 | FALSE | 0.4 | FALSE | -0.25 | FALSE | 0.09 |
|  | Ruminococcus | TRUE | 1.43 | FALSE | -0.19 | FALSE | -0.05 | TRUE | 0.64 | FALSE | 0.02 | FALSE | -0.3 | TRUE | 1.07 | FALSE | -0.48 | FALSE | -0.17 | FALSE | -0.7 |
|  | [Eubacterium]_coprostanoligenes_group | TRUE | -1.37 | FALSE | -0.69 | FALSE | -0.18 | TRUE | -2.56 | FALSE | 0.44 | FALSE | -0.19 | TRUE | -2.45 | FALSE | 0.1 | FALSE | 0.22 | FALSE | 0.2 |
|  | [Eubacterium]_siraeum_group | TRUE | 1.31 | FALSE | -0.94 | FALSE | -0.36 | FALSE | 0.07 | FALSE | -0.27 | FALSE | 0.14 | FALSE | 0.39 | FALSE | -0.02 | FALSE | -0.57 | FALSE | -0.19 |
|  | Ruminococcaceae | FALSE | 0.71 | FALSE | -0.39 | FALSE | -0.08 | FALSE | -0.45 | FALSE | -0.14 | FALSE | 0.52 | FALSE | -0.26 | FALSE | -0.07 | FALSE | 0.49 | FALSE | 0.33 |
|  | NK4A214_group | FALSE | 0.25 | FALSE | 0.32 | FALSE | 0.88 | FALSE | -0.38 | FALSE | 0.45 | FALSE | 0.11 | FALSE | -0.04 | FALSE | 0.3 | FALSE | 0.11 | FALSE | -0.34 |
|  | Oscillospiraceae Family | TRUE | 3.64 | FALSE | -0.1 | FALSE | -0.07 | TRUE | 2.6 | FALSE | 0.47 | FALSE | 0.04 | TRUE | 2.83 | FALSE | 0.26 | FALSE | 0.07 | FALSE | 0.06 |
|  | **Intestinimonas** | FALSE | 0.21 | FALSE | -0.87 | FALSE | -1.17 | FALSE | -0.18 | FALSE | -0.97 | TRUE | -1.86 | FALSE | -0.11 | FALSE | -1.32 | FALSE | -1.06 | FALSE | -1.37 |
|  | **Butyricicoccus** | TRUE | -1.23 | FALSE | -1.16 | FALSE | -1.21 | TRUE | -1.67 | TRUE | -1.85 | FALSE | -1.36 | TRUE | -1.66 | FALSE | -1.27 | TRUE | -2.36 | FALSE | -0.5 |
|  | **UCG-005** | TRUE | -1.42 | TRUE | -1.32 | FALSE | 0.1 | TRUE | -2.83 | FALSE | 0.26 | FALSE | 0.13 | TRUE | -2.59 | FALSE | 0.36 | FALSE | 0.01 | FALSE | -0.17 |
|  | Monoglobus | TRUE | -1.59 | FALSE | -0.23 | FALSE | 0.78 | TRUE | -2.49 | FALSE | 0.51 | FALSE | 0.15 | TRUE | -2.35 | FALSE | 0.24 | FALSE | 0.16 | FALSE | 0.57 |
|  | Colidextribacter | TRUE | 1.2 | FALSE | 0.06 | FALSE | -0.09 | FALSE | 0.18 | FALSE | 0.49 | FALSE | 0.13 | FALSE | 0.37 | FALSE | 0.13 | FALSE | 0.29 | FALSE | 0.34 |
|  | Oscillibacter | FALSE | 0.37 | FALSE | 0.07 | FALSE | -0.12 | FALSE | -0.51 | FALSE | 0.85 | FALSE | -0.52 | FALSE | -0.3 | FALSE | 0.22 | FALSE | 0.18 | FALSE | -0.33 |
|  | **Coriobacteriales_Incertae_Sedis Family** | FALSE | -0.25 | TRUE | -1.86 | TRUE | -1.88 | TRUE | -1.38 | TRUE | -2.17 | FALSE | -0.55 | FALSE | -1.16 | FALSE | -1.75 | FALSE | -1.59 | FALSE | -0.39 |
|  | Adlercreutzia | FALSE | -0.63 | FALSE | -0.39 | FALSE | -1.53 | TRUE | -1.81 | FALSE | -0.6 | FALSE | -0.07 | FALSE | -1.46 | FALSE | -0.6 | FALSE | -0.85 | FALSE | -0.17 |
|  | Lactobacillus | TRUE | 4.09 | FALSE | 1.62 | FALSE | 0.93 | TRUE | 3.09 | FALSE | 1.32 | FALSE | 1.52 | TRUE | 3.23 | FALSE | 1.42 | FALSE | 1.54 | FALSE | 1.27 |
|  | **Bartonella** | TRUE | 1.14 | TRUE | -2.67 | FALSE | -1.67 | TRUE | -1.36 | FALSE | 0.13 | FALSE | 0.44 | FALSE | -1.26 | FALSE | 0.03 | FALSE | 0.83 | FALSE | 0.46 |
|  | **Lachnospiraceae Family** | TRUE | 4.23 | FALSE | -0.46 | TRUE | -0.64 | TRUE | 2.89 | FALSE | 0.24 | FALSE | 0.21 | TRUE | 3.21 | FALSE | 0.02 | FALSE | 0.13 | FALSE | -0.04 |
|  | Lachnospiraceae_UCG-006 | TRUE | 0.64 | FALSE | -1.03 | FALSE | 0.09 | FALSE | -0.53 | FALSE | 0.31 | FALSE | -0.24 | FALSE | -0.37 | FALSE | -0.36 | FALSE | 0.59 | FALSE | 0.01 |
|  | Allobaculum | TRUE | 1.84 | FALSE | 0.44 | FALSE | 0.71 | TRUE | 1.31 | FALSE | -0.04 | FALSE | 0.24 | TRUE | 1.83 | FALSE | -0.29 | FALSE | -0.63 | FALSE | -0.2 |
|  | Lachnospiraceae_NK4A136_group | TRUE | 3.46 | FALSE | -0.08 | FALSE | -0.06 | TRUE | 2.59 | FALSE | -0.03 | FALSE | 0.03 | TRUE | 2.76 | FALSE | -0.07 | FALSE | 0 | FALSE | 0.1 |
|  | Roseburia | TRUE | 1.36 | FALSE | -0.73 | FALSE | -0.14 | FALSE | 0.35 | FALSE | 0.32 | FALSE | -0.54 | FALSE | 0.44 | FALSE | -0.42 | FALSE | 0.53 | FALSE | -0.05 |
|  | Lachnospiraceae_UCG-001 | TRUE | 1.44 | FALSE | -1.12 | FALSE | -0.94 | FALSE | -0.12 | FALSE | 0.61 | FALSE | -0.31 | FALSE | 0.03 | FALSE | 0.46 | FALSE | -0.79 | FALSE | 0.37 |
|  | [Eubacterium]_xylanophilum_group | TRUE | 1.67 | FALSE | -0.86 | FALSE | -0.22 | FALSE | 0.79 | FALSE | -0.54 | FALSE | -0.36 | FALSE | 0.94 | FALSE | -0.42 | FALSE | -0.46 | FALSE | -0.25 |
|  | Lachnoclostridium | TRUE | 1.04 | FALSE | -1.15 | FALSE | 0.41 | FALSE | 0.12 | FALSE | 0.08 | FALSE | -0.55 | FALSE | 0.16 | FALSE | -0.17 | FALSE | -0.05 | FALSE | -0.03 |
|  | **Blautia** | TRUE | -2.68 | TRUE | 1.71 | FALSE | 0.37 | TRUE | -2.97 | FALSE | 0.18 | FALSE | 0.37 | TRUE | -2.63 | FALSE | 0.05 | FALSE | -0.14 | FALSE | 0.22 |
|  |  |  |  |  |  |  |  |  |  |  |  |  |  |  |  |  |  |  |  |  |  |
| **B** | Agaricomycetes Class | TRUE | -0.99 | FALSE | 0.18 | FALSE | -0.18 | TRUE | -1.26 | FALSE | 0.21 | FALSE | 0.56 | TRUE | -1.25 | FALSE | 0.68 | FALSE | 0.24 | FALSE | 0.56 |
|  | Laetiporus | TRUE | 1.01 | FALSE | 0.9 | FALSE | -0.04 | TRUE | 1.68 | FALSE | -0.41 | FALSE | -0.37 | TRUE | 2.07 | FALSE | -0.76 | FALSE | 0.01 | FALSE | -1.09 |
|  | Botryobasidium | FALSE | 0.02 | FALSE | -0.7 | FALSE | 0.74 | FALSE | -0.46 | FALSE | 0.89 | FALSE | 0.3 | FALSE | -0.94 | FALSE | 1.23 | FALSE | 0.61 | FALSE | 1.71 |
|  | Hyphodontia | TRUE | -1 | FALSE | 0.94 | FALSE | 0.1 | FALSE | -0.01 | FALSE | -0.33 | FALSE | -1.01 | FALSE | 0.05 | FALSE | -0.38 | FALSE | -0.61 | FALSE | -0.87 |
|  | Mycena | FALSE | -0.42 | FALSE | 0.14 | FALSE | -1.1 | FALSE | -0.54 | FALSE | -0.1 | FALSE | -0.23 | FALSE | -0.31 | FALSE | 0.53 | FALSE | -0.68 | FALSE | -0.93 |
|  | Solicoccozyma | FALSE | -0.02 | FALSE | -1.19 | FALSE | -1.09 | TRUE | -1.3 | FALSE | 0.57 | FALSE | 0.66 | TRUE | -1.32 | FALSE | 0.64 | FALSE | 0.66 | FALSE | 0.95 |
|  | Ganoderma | FALSE | -0.15 | FALSE | 0.25 | FALSE | 0.5 | FALSE | 0.18 | FALSE | 0.53 | FALSE | -0.51 | FALSE | -0.26 | FALSE | 0.97 | FALSE | 0.49 | FALSE | 0.38 |
|  | **Heterobasidion** | FALSE | -0.52 | FALSE | 0.27 | FALSE | -0.41 | FALSE | 0.94 | TRUE | -1.95 | TRUE | -2.01 | FALSE | 1.52 | TRUE | -2.05 | TRUE | -2.97 | TRUE | -2.52 |
|  | Lycoperdon | FALSE | 0.12 | FALSE | 1.33 | FALSE | -0.72 | FALSE | -0.67 | FALSE | 1.39 | FALSE | 1.75 | FALSE | -0.31 | FALSE | 1.52 | FALSE | 1 | FALSE | 1.01 |
|  | **Clitocybe** | TRUE | -0.9 | TRUE | 2.87 | TRUE | -1.31 | FALSE | -0.06 | FALSE | 0.15 | FALSE | 0.04 | FALSE | 0.28 | FALSE | 0.77 | FALSE | -0.78 | FALSE | -0.85 |
|  | Agaricales Order | FALSE | 0.37 | FALSE | -1.49 | FALSE | -2.36 | TRUE | -1.22 | FALSE | -0.28 | FALSE | 0.77 | FALSE | -1.23 | FALSE | 0.62 | FALSE | -0.54 | FALSE | 1.08 |
|  | Coprinellus | FALSE | 1.21 | FALSE | 0.66 | FALSE | -0.98 | FALSE | 0.95 | FALSE | 0.19 | FALSE | 0.49 | FALSE | 0.43 | FALSE | 0.88 | FALSE | 0.99 | FALSE | 1.6 |
|  | **Hypholoma** | FALSE | -0.4 | TRUE | 2.37 | FALSE | -0.67 | FALSE | 0.16 | FALSE | 0.51 | FALSE | 0.34 | FALSE | 0.51 | FALSE | 0.59 | FALSE | -0.1 | FALSE | -0.29 |
|  | **Rigidoporus** | TRUE | -0.99 | FALSE | 0.32 | FALSE | -0.06 | FALSE | -0.34 | FALSE | -1.42 | FALSE | -0.26 | FALSE | -0.01 | FALSE | -0.73 | TRUE | -1.99 | FALSE | -0.49 |
|  | Melanogaster | FALSE | 0.35 | FALSE | -0.47 | FALSE | 1.62 | FALSE | 0.32 | FALSE | 0.13 | FALSE | 0.59 | FALSE | -0.62 | FALSE | 1.88 | FALSE | -0.63 | FALSE | 3.11 |
|  | Acremonium | FALSE | -0.53 | FALSE | -0.12 | FALSE | -0.44 | TRUE | -1.16 | FALSE | 0.79 | FALSE | 0.51 | FALSE | -1.13 | FALSE | 0.52 | FALSE | 0.32 | FALSE | 1.16 |
|  | Phallus | FALSE | -0.11 | FALSE | -0.41 | FALSE | 0.46 | FALSE | -0.47 | FALSE | 0.18 | FALSE | 0.61 | FALSE | -1 | FALSE | 1.43 | FALSE | 0.12 | FALSE | 1.8 |
|  | **Cordyceps** | FALSE | -0.48 | FALSE | 1.05 | TRUE | 2 | FALSE | 0.75 | FALSE | 0.02 | FALSE | -0.43 | FALSE | 0.82 | FALSE | 0.05 | FALSE | 0.08 | FALSE | -0.54 |
|  | **Vishniacozyma** | FALSE | 1.91 | FALSE | 0.3 | TRUE | -2.34 | FALSE | 1.56 | FALSE | -0.77 | FALSE | 0.06 | FALSE | 1.49 | FALSE | -0.28 | FALSE | 0.67 | FALSE | -0.15 |
|  | Mucor | TRUE | 7.46 | FALSE | -1.77 | FALSE | -0.59 | TRUE | 6.52 | FALSE | 0.23 | FALSE | -0.17 | TRUE | 6.85 | FALSE | 0.54 | FALSE | -0.48 | FALSE | -1.03 |
|  | Mucoraceae Family | FALSE | -0.4 | FALSE | -1.18 | FALSE | 0.44 | FALSE | -0.99 | FALSE | 0.59 | FALSE | 0.07 | FALSE | -0.86 | FALSE | 0.26 | FALSE | 1.12 | FALSE | -0.33 |
|  | Mucoraceae_gen_Incertae_sedis | TRUE | 1.77 | FALSE | -1.36 | FALSE | 1.07 | FALSE | 1.36 | FALSE | 0.17 | FALSE | 0.18 | FALSE | 0.98 | FALSE | 0.79 | FALSE | 0.47 | FALSE | 1.05 |
|  | **Pilaira** | FALSE | 0.65 | TRUE | -1.31 | FALSE | -0.41 | FALSE | 0.22 | FALSE | -0.33 | FALSE | -0.39 | FALSE | 0.15 | FALSE | 0 | FALSE | -0.43 | FALSE | 0.02 |
|  | **Ascomycota Phylum** | FALSE | 0.38 | FALSE | 0.87 | FALSE | -0.05 | TRUE | 1.54 | FALSE | -0.8 | FALSE | -1.26 | TRUE | 1.83 | FALSE | -0.96 | TRUE | -1.77 | FALSE | -1.13 |
|  | Malassezia | FALSE | 0.72 | FALSE | 0.38 | FALSE | 0.33 | FALSE | 1.05 | FALSE | -0.18 | FALSE | -0.02 | FALSE | 1.36 | FALSE | 0.09 | FALSE | -0.01 | FALSE | -0.96 |
|  | **Trechispora** | FALSE | 0.58 | FALSE | 0.5 | FALSE | 1.05 | FALSE | 0.24 | FALSE | 1.19 | FALSE | 1.2 | FALSE | -0.04 | TRUE | 1.96 | FALSE | 1.57 | FALSE | 1.31 |
|  | **Buckleyzyma** | FALSE | 0.65 | FALSE | -0.3 | TRUE | -1.67 | FALSE | -0.34 | FALSE | 0.19 | FALSE | 0.74 | FALSE | -0.36 | FALSE | 0.81 | FALSE | 0.64 | FALSE | 0.54 |
|  | Erythrobasidium | FALSE | -0.27 | FALSE | 1.15 | FALSE | -1.41 | FALSE | 0.31 | FALSE | -0.64 | FALSE | -0.67 | FALSE | 0.48 | FALSE | -0.56 | FALSE | -0.25 | FALSE | -1.04 |
|  | Cystobasidium | FALSE | 0.01 | FALSE | -0.36 | TRUE | -1.96 | FALSE | -0.92 | FALSE | 0.05 | FALSE | 0.44 | FALSE | -0.75 | FALSE | 0.58 | FALSE | 0.72 | FALSE | -0.54 |
|  | Symmetrospora | FALSE | -0.38 | FALSE | -0.02 | FALSE | -1.03 | FALSE | -0.48 | FALSE | -0.56 | FALSE | -0.11 | FALSE | -0.42 | FALSE | 0.08 | FALSE | -0.71 | FALSE | -0.11 |
|  | Candida | FALSE | 0.9 | FALSE | 1.6 | FALSE | 0.43 | FALSE | 1.68 | FALSE | 0.02 | FALSE | 0.12 | TRUE | 2.09 | FALSE | -0.26 | FALSE | 0.37 | FALSE | -0.77 |
|  | **Cystobasidiomycetes_gen_Incertae_sedis** | FALSE | -0.5 | FALSE | -0.24 | TRUE | -1.36 | FALSE | -0.63 | FALSE | -0.53 | FALSE | -0.47 | FALSE | -0.77 | FALSE | 0.06 | FALSE | -0.09 | FALSE | -0.34 |
|  | **Mortierella** | FALSE | 0 | TRUE | 1.94 | FALSE | 1.21 | FALSE | 0.29 | FALSE | 1.74 | FALSE | 0.96 | FALSE | 0.52 | FALSE | 1.33 | FALSE | 1.57 | FALSE | 0.4 |
|  | Apiotrichum | FALSE | -0.19 | FALSE | 0.06 | FALSE | -0.72 | FALSE | -0.38 | FALSE | 0.04 | FALSE | 0.01 | FALSE | -0.44 | FALSE | 0.02 | FALSE | 0.67 | FALSE | 0.24 |
|  | Capnodiales_gen_Incertae_sedis | FALSE | -0.16 | FALSE | -0.25 | FALSE | -1.29 | FALSE | -0.49 | FALSE | -0.42 | FALSE | -0.09 | FALSE | -0.16 | FALSE | -0.18 | FALSE | -0.7 | FALSE | -0.6 |
|  | **Chaetothyriales Order** | FALSE | 0.32 | FALSE | 0.04 | FALSE | -1.2 | FALSE | 0.99 | FALSE | -1.34 | FALSE | -1.37 | TRUE | 1.51 | FALSE | -1.43 | TRUE | -2.33 | TRUE | -1.8 |
|  | Cladophialophora | FALSE | 0.48 | FALSE | -0.74 | FALSE | -0.21 | FALSE | 0.28 | FALSE | -0.33 | FALSE | -0.2 | FALSE | 0.13 | FALSE | -0.01 | FALSE | -0.27 | FALSE | 0.49 |
|  | **Cyphellophoraceae Family** | FALSE | 0.8 | FALSE | 0.03 | TRUE | -2 | FALSE | 0.56 | FALSE | -0.66 | FALSE | -0.31 | FALSE | 0.77 | FALSE | -0.48 | FALSE | -0.25 | FALSE | -0.76 |
|  | **Neophaeococcomyces** | FALSE | -0.24 | FALSE | -0.48 | TRUE | -1.69 | FALSE | -0.73 | FALSE | -0.56 | FALSE | -0.12 | FALSE | -0.76 | FALSE | 0.37 | FALSE | -0.92 | FALSE | 0.03 |
|  | Sordariales Order | FALSE | 0.06 | FALSE | 0.37 | FALSE | 1.01 | FALSE | -0.09 | FALSE | 1.26 | FALSE | 0.54 | FALSE | -0.18 | FALSE | 0.74 | FALSE | 2.27 | FALSE | 0.58 |
|  | Sordariomycetes Class | TRUE | -0.7 | FALSE | 0.09 | FALSE | -0.37 | TRUE | -0.81 | FALSE | -0.15 | FALSE | 0.2 | FALSE | -0.88 | FALSE | 0.06 | FALSE | 0.49 | FALSE | 0.43 |
|  | **Cytospora** | FALSE | 0.83 | FALSE | 0.79 | TRUE | -2.77 | FALSE | -0.19 | FALSE | 0.93 | FALSE | 0.7 | FALSE | -0.23 | FALSE | 0.58 | FALSE | 1.82 | FALSE | 0.79 |
|  | Pseudeurotiaceae Family | TRUE | 2.61 | FALSE | 0.34 | FALSE | 0.44 | TRUE | 2.46 | FALSE | 0.58 | FALSE | 0.61 | TRUE | 2.19 | FALSE | 0.87 | FALSE | 1.06 | FALSE | 1.29 |
|  | Pleosporales Order | TRUE | 1.29 | FALSE | 0.72 | FALSE | -1.06 | FALSE | 1.27 | FALSE | 0.35 | FALSE | -0.16 | FALSE | 1.41 | FALSE | 0.09 | FALSE | 0.1 | FALSE | -0.12 |
|  | Preussia | FALSE | -0.28 | FALSE | -0.04 | FALSE | 0.07 | FALSE | -0.34 | FALSE | 0.36 | FALSE | -0.1 | FALSE | -0.49 | FALSE | 0.8 | FALSE | -0.1 | FALSE | 0.39 |
|  | Dothideomycetes Class | FALSE | -0.6 | FALSE | 0.12 | FALSE | -0.77 | FALSE | -0.08 | FALSE | -1.15 | FALSE | -0.79 | FALSE | 0 | FALSE | -0.62 | FALSE | -1.41 | FALSE | -0.63 |
|  | **Aureobasidium** | TRUE | 3.24 | FALSE | -0.74 | TRUE | -3.35 | TRUE | 1.91 | FALSE | -0.83 | FALSE | 0.63 | FALSE | 1.61 | FALSE | 0.09 | FALSE | 1.03 | FALSE | 0.83 |
|  | **Periconia** | FALSE | 0.46 | FALSE | -1.05 | FALSE | 0.22 | FALSE | 1.17 | FALSE | -1.25 | FALSE | -1.7 | FALSE | 1.21 | FALSE | -0.62 | TRUE | -2.35 | FALSE | -1.37 |
|  | **Pseudopithomyces** | FALSE | -0.34 | FALSE | -0.75 | FALSE | 0.36 | FALSE | 0.41 | FALSE | -0.99 | TRUE | -1.57 | FALSE | 0.34 | FALSE | -0.66 | FALSE | -1.62 | FALSE | -0.97 |
|  | **Nigrograna** | FALSE | -0.11 | FALSE | 0.53 | FALSE | -0.9 | TRUE | -1.23 | FALSE | 1.17 | TRUE | 1.73 | FALSE | -1.23 | FALSE | 1.04 | FALSE | 1.4 | TRUE | 2.26 |
|  | Keissleriella | FALSE | 0.68 | FALSE | 0.77 | FALSE | -0.65 | FALSE | 0.7 | FALSE | 0.4 | FALSE | 0.05 | FALSE | 0.97 | FALSE | 0.13 | FALSE | -0.93 | FALSE | 0.44 |
|  | **Hypocreales_gen_Incertae_sedis** | FALSE | -0.32 | FALSE | -0.38 | FALSE | -1.29 | TRUE | -1.54 | FALSE | 0.96 | FALSE | 0.94 | TRUE | -1.71 | FALSE | 1.04 | TRUE | 1.85 | FALSE | 1.12 |
|  | Arthroderma | FALSE | -0.26 | FALSE | -0.03 | FALSE | 0.92 | FALSE | 0.1 | FALSE | -0.44 | FALSE | 0.04 | FALSE | -0.09 | FALSE | 0.2 | FALSE | 0.31 | FALSE | 0.3 |
|  | Aspergillus | TRUE | 1.57 | FALSE | 1.13 | FALSE | 0.17 | TRUE | 2.17 | FALSE | -0.06 | FALSE | -0.08 | TRUE | 2.35 | FALSE | -0.52 | FALSE | 0.6 | FALSE | -0.2 |
|  | **Pyrenochaetopsis** | FALSE | -0.51 | FALSE | 0.05 | FALSE | -0.32 | FALSE | 0.24 | FALSE | -0.57 | TRUE | -1.48 | FALSE | 0.47 | FALSE | -0.78 | TRUE | -1.79 | FALSE | -1.2 |
|  | Paraphaeosphaeria | TRUE | -0.78 | FALSE | -0.46 | FALSE | -0.7 | TRUE | -0.99 | FALSE | -0.08 | FALSE | -0.36 | TRUE | -1.13 | FALSE | 0.41 | FALSE | -0.47 | FALSE | 0.14 |
|  | **Botrytis** | FALSE | 0.59 | TRUE | -2.05 | FALSE | -1.16 | FALSE | 0.3 | FALSE | -1.43 | FALSE | -1.21 | FALSE | 0.32 | FALSE | -1.06 | FALSE | -1.18 | FALSE | -1.05 |
|  | Boeremia | TRUE | 2.66 | FALSE | -0.32 | FALSE | -1.59 | TRUE | 2.75 | FALSE | -1.42 | FALSE | -0.63 | TRUE | 2.83 | FALSE | -1.03 | FALSE | -0.82 | FALSE | -0.62 |
|  | Didymellaceae Family | FALSE | 0.55 | FALSE | -0.19 | FALSE | -0.27 | FALSE | 0.88 | FALSE | -0.71 | FALSE | -0.65 | FALSE | 0.94 | FALSE | -0.83 | FALSE | -0.18 | FALSE | -0.51 |
|  | **Neosetophoma** | FALSE | 0.04 | FALSE | 0.67 | TRUE | -1.61 | FALSE | 0.1 | FALSE | 0.23 | FALSE | -0.67 | FALSE | 0.2 | FALSE | 0.44 | FALSE | -0.9 | FALSE | -0.6 |
|  | **Stagonospora** | FALSE | -0.74 | FALSE | 1.2 | TRUE | -1.57 | TRUE | -1.14 | FALSE | 0.3 | FALSE | 0.91 | FALSE | -0.9 | FALSE | 0.47 | FALSE | 0.19 | FALSE | 0.65 |
|  | Helotiales Order | FALSE | 0.02 | FALSE | 0.04 | FALSE | -0.18 | FALSE | 0.67 | FALSE | -1.1 | FALSE | -0.84 | FALSE | 0.68 | FALSE | -0.76 | FALSE | -1.06 | FALSE | -0.5 |
|  | Thelebolus | TRUE | 2.25 | FALSE | -0.46 | FALSE | -2.03 | FALSE | 1.26 | FALSE | 0.21 | FALSE | 0.33 | FALSE | 0.99 | FALSE | 1.1 | FALSE | 0.35 | FALSE | 0.68 |
|  | **Leotiomycetes Class** | TRUE | 5.36 | FALSE | -1.81 | TRUE | -2.74 | TRUE | 3.3 | FALSE | 0.18 | FALSE | 0.97 | TRUE | 3.21 | FALSE | 0.93 | FALSE | 0.35 | FALSE | 1.17 |
|  | Penicillium | TRUE | 4.88 | FALSE | -0.01 | FALSE | -0.16 | TRUE | 4.94 | FALSE | 0.3 | FALSE | -0.45 | TRUE | 5.14 | FALSE | -0.11 | FALSE | -0.54 | FALSE | -0.18 |
|  | Cladosporium | TRUE | 3.5 | FALSE | 0.71 | FALSE | 0.14 | TRUE | 4.66 | FALSE | -1.1 | FALSE | -1.11 | TRUE | 4.6 | FALSE | -0.97 | FALSE | -1.04 | FALSE | -0.45 |
|  | Aspergillaceae Family | FALSE | -0.32 | FALSE | 0.08 | FALSE | -0.3 | FALSE | -0.28 | FALSE | 0.2 | FALSE | -0.33 | FALSE | -0.12 | FALSE | 0.13 | FALSE | -0.44 | FALSE | -0.27 |
|  | Oidiodendron | FALSE | 0.87 | FALSE | 0.66 | FALSE | 0.67 | FALSE | 1.25 | FALSE | 0.5 | FALSE | -0.08 | FALSE | 0.99 | FALSE | 0.43 | FALSE | 0.25 | FALSE | 1.1 |
|  | Taphrina | FALSE | 1.34 | FALSE | 1.07 | FALSE | -1.27 | FALSE | 1.32 | FALSE | -0.29 | FALSE | 0.49 | FALSE | 1.1 | FALSE | 0.73 | FALSE | 0.27 | FALSE | 0.71 |
|  | **Podospora** | TRUE | -0.93 | FALSE | -0.56 | TRUE | -1.17 | TRUE | -1.83 | FALSE | 0.6 | FALSE | 0.32 | TRUE | -1.95 | FALSE | 0.84 | FALSE | 0.72 | FALSE | 0.59 |
|  | Phialemonium | FALSE | -0.08 | FALSE | -0.03 | FALSE | 0.17 | FALSE | 0.2 | FALSE | -0.16 | FALSE | -0.45 | FALSE | -0.03 | FALSE | -0.32 | FALSE | 0.22 | FALSE | 0.57 |
|  | **Hypocreales Order** | FALSE | 0.71 | FALSE | -0.61 | FALSE | -0.82 | FALSE | -0.95 | TRUE | 1.65 | FALSE | 1.57 | FALSE | -0.84 | FALSE | 1.71 | TRUE | 1.79 | FALSE | 1.14 |
|  | Keithomyces | FALSE | -0.04 | FALSE | 0.39 | FALSE | 0.08 | FALSE | -0.08 | FALSE | 0.38 | FALSE | 0.28 | FALSE | -0.38 | FALSE | 0.53 | FALSE | 1.12 | FALSE | 0.99 |
|  | Gibellulopsis | FALSE | -0.8 | FALSE | 0.06 | FALSE | -0.16 | FALSE | -0.13 | FALSE | -0.44 | FALSE | -1.27 | FALSE | 0.13 | FALSE | -0.7 | FALSE | -1.4 | FALSE | -1.21 |
|  | Samsoniella | TRUE | -0.87 | FALSE | -0.04 | FALSE | 0.51 | FALSE | -0.86 | FALSE | 0.1 | FALSE | 0.21 | FALSE | -0.49 | FALSE | -0.26 | FALSE | 0.34 | FALSE | -0.35 |
|  | Microascus | FALSE | -0.62 | FALSE | -0.14 | FALSE | 0 | FALSE | -0.67 | FALSE | -0.32 | FALSE | 0.21 | FALSE | -0.4 | FALSE | -0.28 | FALSE | -0.07 | FALSE | -0.17 |
|  | Cephalotrichum | TRUE | 1.45 | FALSE | -0.13 | FALSE | 0.59 | TRUE | 1.5 | FALSE | 0.09 | FALSE | 0.11 | FALSE | 1.32 | FALSE | 0 | FALSE | 0.93 | FALSE | 0.68 |
|  | Gamsia | FALSE | -0.11 | FALSE | -0.76 | FALSE | -0.24 | FALSE | -0.6 | FALSE | 0.03 | FALSE | 0.2 | FALSE | -0.82 | FALSE | 0.23 | FALSE | 0.8 | FALSE | 0.76 |
|  | Trichoderma | TRUE | 1.55 | FALSE | 0.28 | FALSE | 1.5 | TRUE | 1.6 | FALSE | 0.42 | FALSE | 0.88 | FALSE | 1.64 | FALSE | 0.51 | FALSE | 0.13 | FALSE | 1.43 |
|  | Fusarium | TRUE | 1.41 | FALSE | 0.64 | FALSE | -0.19 | TRUE | 1.94 | FALSE | -0.56 | FALSE | -0.31 | TRUE | 2.01 | FALSE | -0.35 | FALSE | 0.19 | FALSE | -0.58 |
|  | Cosmospora | TRUE | 1.24 | FALSE | -1.05 | FALSE | -0.94 | FALSE | 0.45 | FALSE | 0.45 | FALSE | -0.15 | FALSE | 0.42 | FALSE | -0.06 | FALSE | 0.37 | FALSE | 0.6 |
|  | Chaetosphaeriaceae Family | FALSE | -0.71 | FALSE | 0.5 | FALSE | 0.22 | FALSE | -1.08 | FALSE | 0.81 | FALSE | 0.96 | FALSE | -1.26 | FALSE | 1.37 | FALSE | 0.45 | FALSE | 1.59 |
|  | Chloridium | FALSE | 0.16 | FALSE | -0.6 | FALSE | 0.35 | FALSE | -0.75 | FALSE | 1.04 | FALSE | 1.02 | FALSE | -0.62 | FALSE | 1.25 | FALSE | 0.43 | FALSE | 0.97 |
|  | Neoschizothecium | FALSE | 0.26 | FALSE | -0.1 | FALSE | -1.28 | FALSE | -0.02 | FALSE | 0 | FALSE | -0.29 | FALSE | 0.3 | FALSE | -0.07 | FALSE | -0.38 | FALSE | -0.8 |
|  | Metapochonia | FALSE | 0.36 | FALSE | -0.76 | FALSE | -0.33 | FALSE | -0.1 | FALSE | 0.1 | FALSE | 0 | FALSE | -0.14 | FALSE | -0.06 | FALSE | 0.42 | FALSE | 0.47 |
|  | Volutella | FALSE | -0.47 | FALSE | -0.17 | FALSE | -1 | TRUE | -1.58 | FALSE | 1.2 | FALSE | 0.93 | TRUE | -1.57 | FALSE | 1.14 | FALSE | 1.3 | FALSE | 0.96 |
|  | Cordycipitaceae Family | FALSE | -0.18 | FALSE | -0.14 | FALSE | 0.27 | FALSE | 0.62 | FALSE | -0.9 | FALSE | -1.19 | FALSE | 0.87 | FALSE | -1.45 | FALSE | -0.38 | FALSE | -1.36 |
|  | **Sarocladium** | FALSE | -0.53 | FALSE | -0.6 | FALSE | -0.36 | FALSE | -0.26 | FALSE | -0.68 | FALSE | -0.99 | FALSE | -0.11 | FALSE | -0.19 | TRUE | -1.81 | FALSE | -0.91 |
|  | Gibberella | FALSE | -0.21 | FALSE | 0.17 | FALSE | -1.38 | FALSE | -0.45 | FALSE | -0.36 | FALSE | 0.06 | FALSE | -0.05 | FALSE | -0.65 | FALSE | 0.31 | FALSE | -0.75 |
|  | **Gymnostellatospora** | FALSE | -0.47 | FALSE | -0.25 | FALSE | -0.66 | TRUE | -1.73 | FALSE | 1.13 | TRUE | 1.45 | TRUE | -1.63 | FALSE | 0.88 | TRUE | 2.14 | FALSE | 1.09 |

## Supplementary Figures


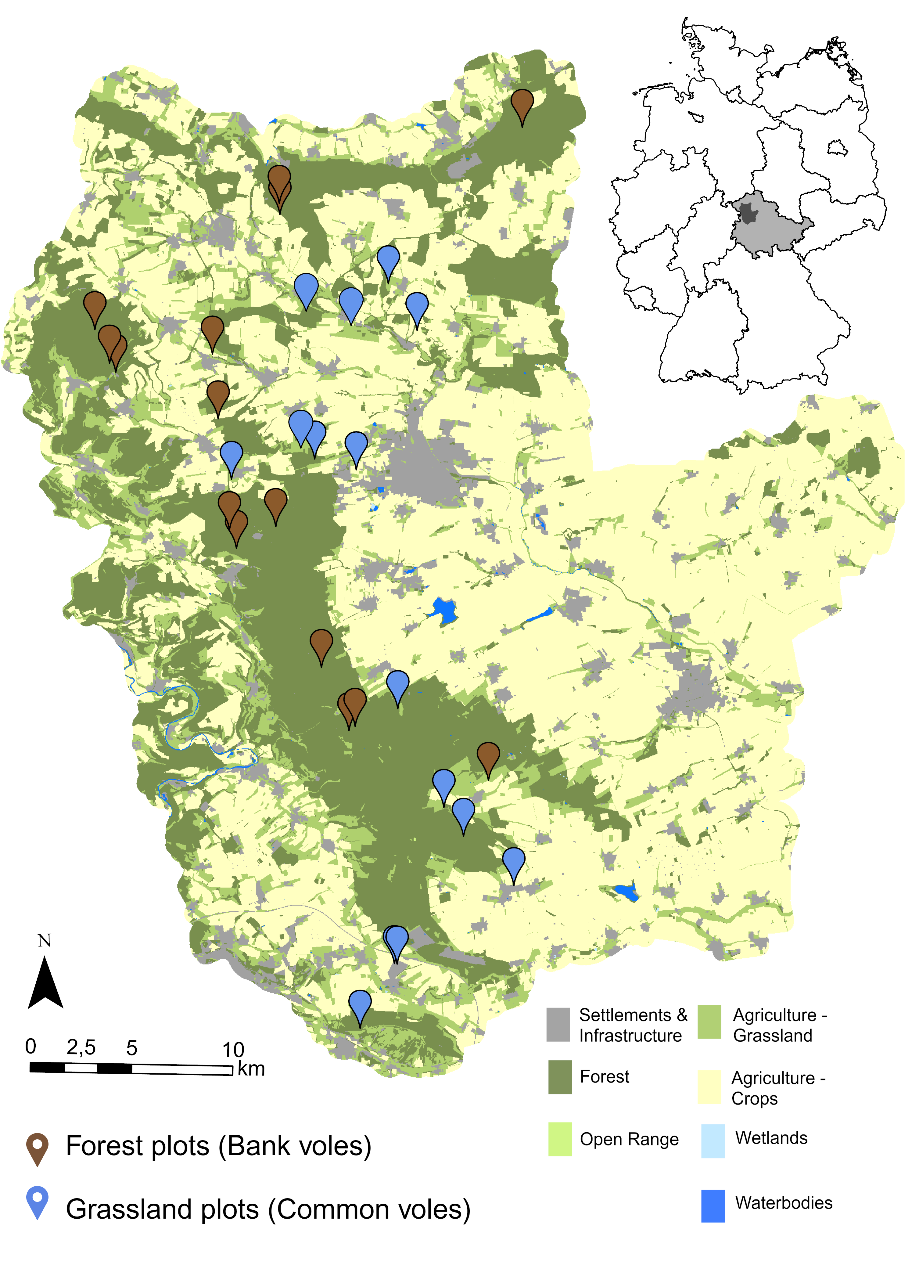


**Figure S1:** Map of the plots sampled in the Hanich-Dün exploratory in Thuringia, Germany. The map was created using ArcGIS Pro, version 3.1.1.


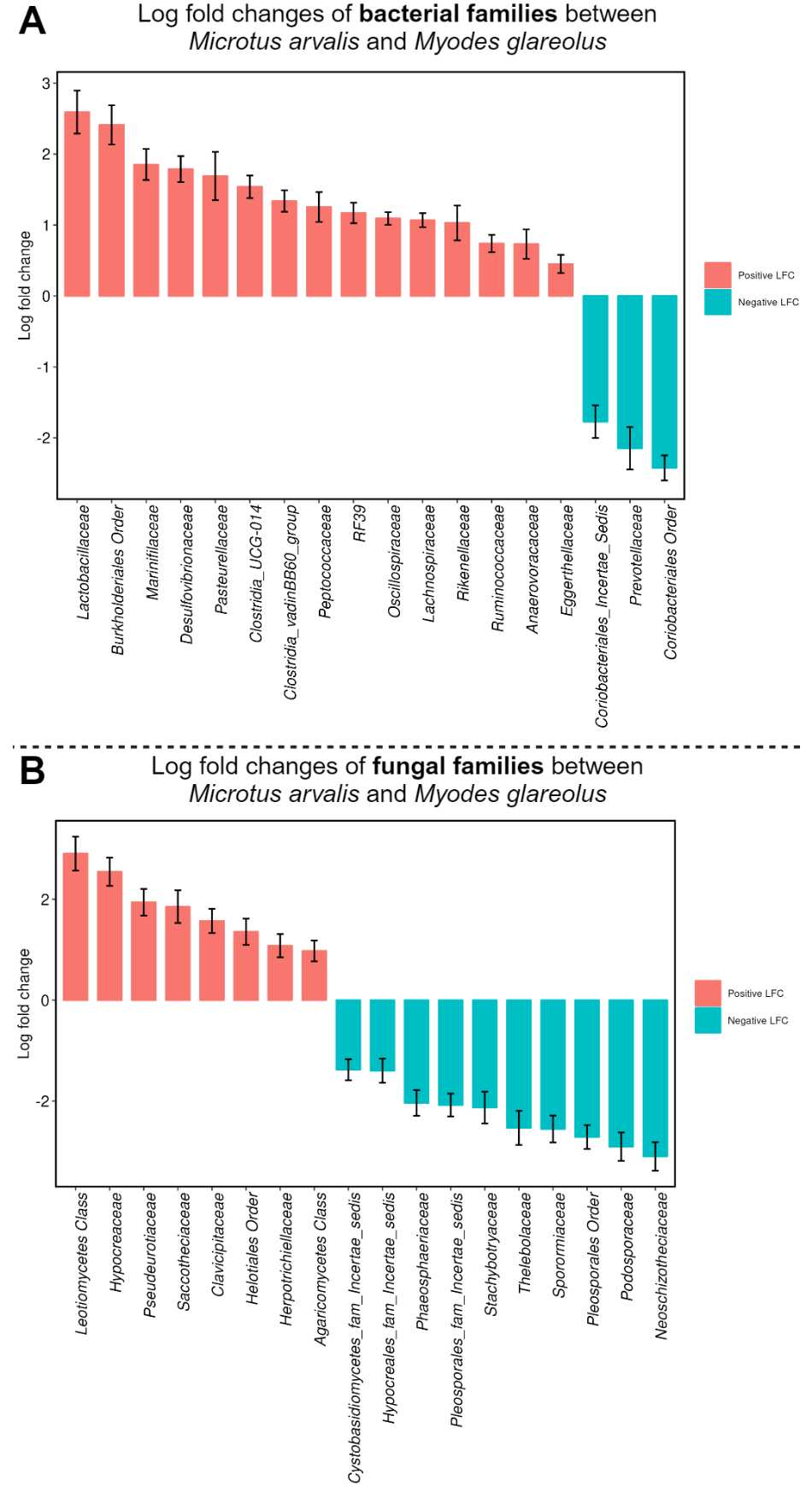


**Figure S2:** Waterfall plots of bacterial (A) and fungal (B) families which differ significantly between common voles (*M. arvalis*) and bank voles (*M. glareolus*)

**Reference**

Dore, J., Ehrlich, S.D., Levenez, F., Roume, H., Morabito, C. and IHMS Consortium. (2020). *IHMS_SOP 06 V3: Standard operating procedure for fecal samples DNA extraction, Protocol Q.*
